# Synthesis of 3(2)-phosphonylated thiazolo[3,2-*a*]oxopyrimidines

**DOI:** 10.3762/bjoc.16.161

**Published:** 2020-08-10

**Authors:** Ksenia I Kaskevich, Anastasia A Babushkina, Vladislav V Gurzhiy, Dmitrij M Egorov, Nataly I Svintsitskaya, Albina V Dogadina

**Affiliations:** 1 Department of Organic Chemistry, St. Petersburg State Institute of Technology, Moskovskii pr. 26, St. Petersburg, 190013, Russia; 2 Department of Crystallography, Institute of Earth Sciences, St. Petersburg State University, Universitetskaya emb. 7/9, St. Petersburg, 199034 Russia

**Keywords:** chloroethynylphosphonate, heterocyclization, phosphonylated thiazolopyrimidines, phosphonylation, thiazolopyrimidine, 2-thiouracil

## Abstract

A series of 3(2)-phosphonylated thiazolo[3,2-*a*]oxopyrimidines was synthesized for the first time by the reactions of chloroethynylphosphonates with unsubstituted and 5(6)-substituted 2-thiouracils. The reaction of chloroethynylphosphonates with 6-substituted 2-thiouracils bearing electron-donor groups (CH_3_, Ph) proceeded with high regioselectivity involving the cyclization through the N^3^-nitrogen atom to form new 3-phosphonylated thiazolo[3,2-*a*]-5-oxopyrimidines with good yield. In the case of unsubstituted and 5-methyl-2-thiouracils, cyclization occurred predominantly through the N^1^ atom and partially via the N^3^-nitrogen atom to form a mixture of the corresponding thiazolo[3,2-*a*]-7- and 5-oxopyrimidines. A dramatic change in the reaction regioselectivity was observed in the case of 6-trifluoromethyl-2-thiouracil that afforded 2- and 3-phosphonylated 5-oxothiazolopyrimidines in a 1:1 ratio.

## Introduction

**Figure 1:**
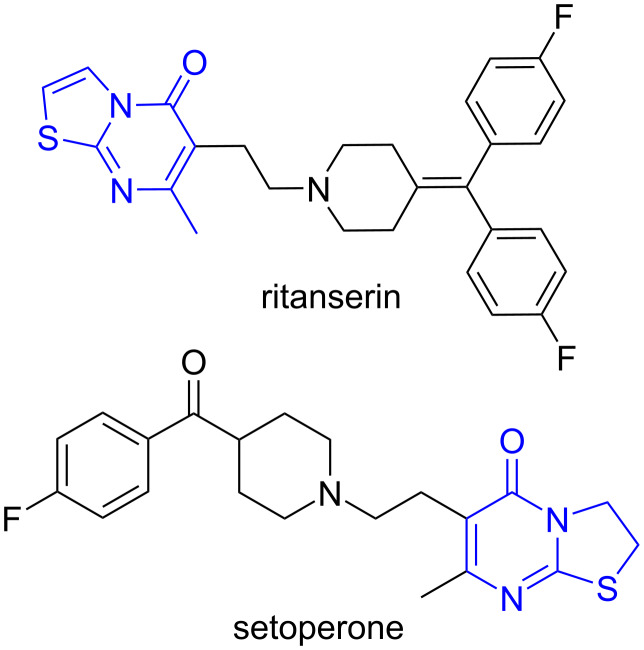
Structure of ritanserin and setoperone drugs.

Thiazolopyrimidines, whose molecules includes both thiazole and pyrimidine rings, have a structural analogy with the antipsychotic drugs ritanserin and setoperone (Figure 1) [1–3]. To date, a wide spectrum of biological activity of thiazolopyrimidines has been determined: anticancer [4–5], antimicrobial [6–7], anti-inflammatory [8–9], and antiviral [10–11].

**Scheme 1:**
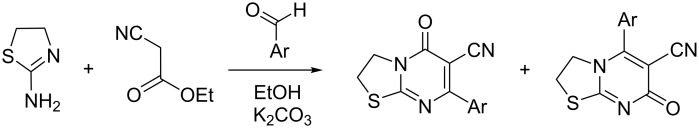
One-pot synthesis of 5(7)-oxothiazolopyrimidine-6-carbonitriles.

The best known methods for the preparation of thiazolopyrimidines are based on condensation reactions. The most commonly used synthesis is the three-component condensation of 2-aminothiazoline, aromatic aldehyde, and ethyl cyanoacetate, which leads to the formation of 5- and 7-oxothiazolopyrimidine-6-carbonitriles (Scheme 1) [12–13].

**Scheme 2:**
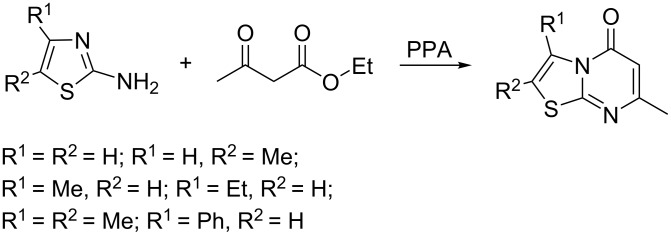
Synthesis of thiazolopyrimidine-5-ones through the reaction of 2-aminothiazoles with ethyl acetoacetate.

The synthesis of thiazolopyrimidines through the reaction of 2-aminothiazoles with 1,3-ketoesters in the presence of acids, bases or condensing agents (Scheme 2) has been studied fairly well [6,8,14–16].

**Scheme 3:**
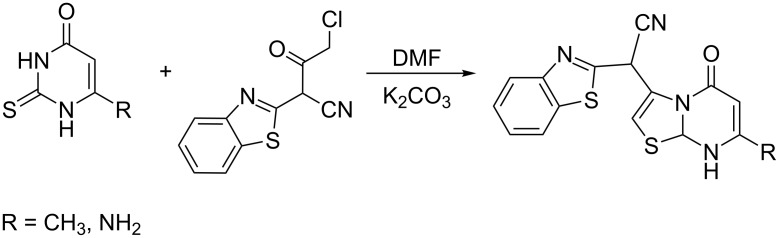
Synthesis of 2-(benzo[*d*]thiazol-2-yl)-2-(7-R-5-oxo-5*H*-thiazolo[3,2-*a*]pyrimidin-3-yl)acetonitriles.

The most accessible approach to the synthesis of 5*H*-thiazolo[3,2-*a*]pyrimidine-5(7)-ones is the reaction of 2-thiouracil derivatives with α-halo ketones and α-halo acids, involving successive alkylation and condensation steps (Scheme 3) [17–21].

**Scheme 4:**
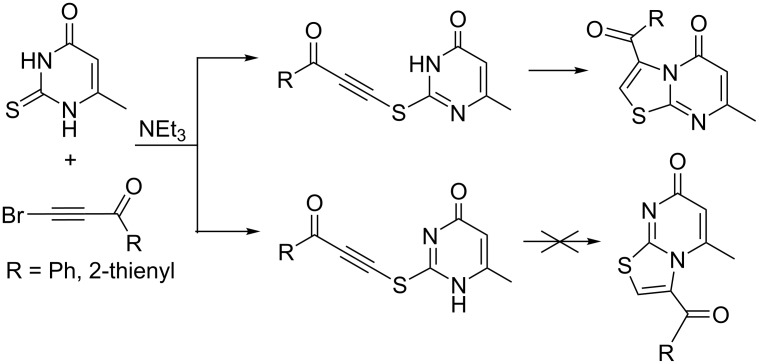
Synthesis of 3-acyl-7-methyl-5*H*-thiazolo[3,2-*a*]pyrimidin-5-ones.

A convenient one-step synthesis of thiazolopyrimidine-5-ones by reacting 6-methyl-2-thiouracils with bromoethynylketones has been reported by Shishkin and co-workers (Scheme 4) [20]. The authors for the first time proved the structure of the 5-oxo isomer by single crystal X-ray diffraction analysis.

**Scheme 5:**
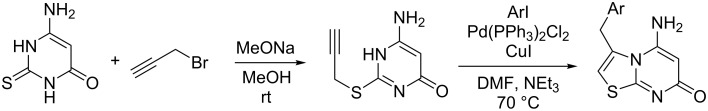
Sonogashira coupling reaction of 6-amino-2-thiouracil with propargyl bromide.

The Pd-catalyzed Sonogashira coupling reaction between 2-thiouracil and propargyl bromide yielded 5*H*-thiazolo[3,2-*a*]pyrimidine-5-one (Scheme 5) [20–24].

Despite the wide variety of thiazolopyrimidines reported to date, phosphonylated analogues of compounds of this series are unknown. Of special interest is the design of molecules containing practically significant heteroaromatic rings and a biologically active and hydrolysis-resistant phosphonate group, as it has been reported that the combination of several pharmacophore fragments in one molecule can lead to a synergistic increase in biological activity or an additional variety of the latter [22–28].

Herein, we report the synthesis of a new series of phosphonylated thiazolopyrimidines. In our studies, chloroethynylphosphonate was used as the phosphonylating agent, which allowed the formation of a thiazole ring with simultaneous phosphonylation of the latter. As the second reaction component, available 2-thiouracils were chosen as the most studied objects used for creating thiazolopyrimidine systems. The main objective of the study was to determine the regioselectivity of the reaction. Literature data mainly report the formation of 5-oxopyrimidines by cyclization through the N^3^ atom of the starting 2-thiouracil [14–24]. The formation of 7-oxopyrimidines by cyclization through the N^1^ atom has been noted only in a few reports [29–32]. However, reliable data for the identification of the 5- and 7-oxo isomers are not available to date and the determination of the structures of 5- and 7-oxo isomers were mainly based on ^1^H NMR spectroscopy. The most comprehensive and convincing evidence for the formation of a 5-oxothiazolopyrimidine was provided by Shishkin and co-workers [20], who performed single crystal X-ray diffraction analysis along with ^1^H and ^13^C NMR spectral studies. Unfortunately, the majority of reports on the synthesis of thiazolopyrimidines relied on ^1^H NMR data to prove the structure of the obtained compounds [18,33–35]. There are no systematic data on ^13^C NMR spectroscopy of thiazolopyrimidines, which could be used as an additional approach to estimate the regioselectivity of the reaction. A single example of the use of ^13^C NMR spectroscopy for unambiguous establishing the structure of thiazolo[3,2-*a*]pyrimidines obtained was given by Iranian researchers [36], but only for the 5-oxo isomers. In our opinion, the presence of a phosphorus fragment in a thiazolo[3,2-*a*]pyrimidine molecule significantly facilitates the determination of the structure by means of ^13^C, ^1^H, and ^31^P NMR spectroscopy methods.

## Results and Discussion

**Scheme 6:**
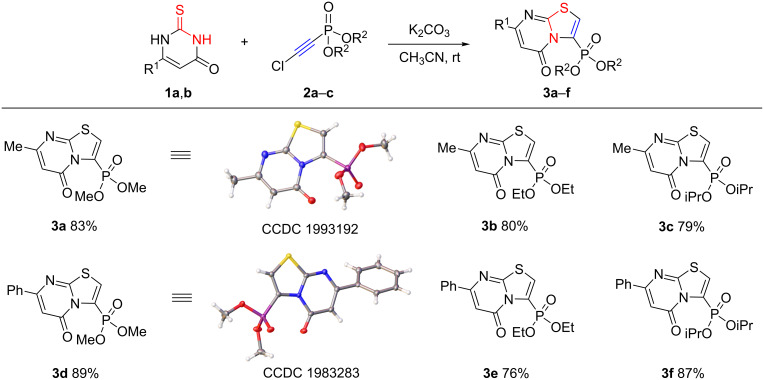
Reactions of 6-substituted 2-thiouracils **1a**,**b** with chloroethynylphosphonates **2a**–**c**.

Aiming to synthesize a new series of phosphonylated thiazolopyrimidines, we performed reactions of unsubstituted and substituted 2-thiouracils **1a**–**e** with chloroethynylphosphonates **2a**–**c**. We found that the reaction with 6-substituted 2-thiouracils bearing either methyl or phenyl groups occurred regioselectively with a N^3^ atom ring-closure to afford the 3-phosphonylated thiazolo[3,2-*a*]-5-oxopyrimidines **3a**–**f** in good yields (Scheme 6). Likely, in this case the attack by the N^3^ atom was more favorable than by the N^1^ atom due to the steric effect of the substituent in position 6. The reactions proceeded under mild conditions within 3–5 hours. Anhydrous K_2_CO_3_ was used as a base to neutralize HCl formed during the reaction. The need for the use of anhydrous solvents and reagents is caused by the possibility of the formation of byproducts, if any, due to hydrolysis as we have noted earlier in the case of the reactions of chloroethynylphosphonates with nitrogen-containing nucleophiles [37–39].

The assignment of the reaction product to the 5-oxo isomer was made based on ^13^C NMR spectral analysis: the carbon atoms of the CH=СR fragment (R=CH_3_, Ph) are represented by a strong signal at δ_C_ 101–105 ppm (СН=) and a weak signal at δ_C_ 157–158 ppm (=CR). These data coincide with those for 3-phenylthiazolo[3,2-*a*]-5-oxopyrimidine [40]. In addition, the structures of the phosphonylated thiazolopyrimidines **3a** and **3d** were unambiguously confirmed by single crystal X-ray diffraction data.

**Scheme 7:**
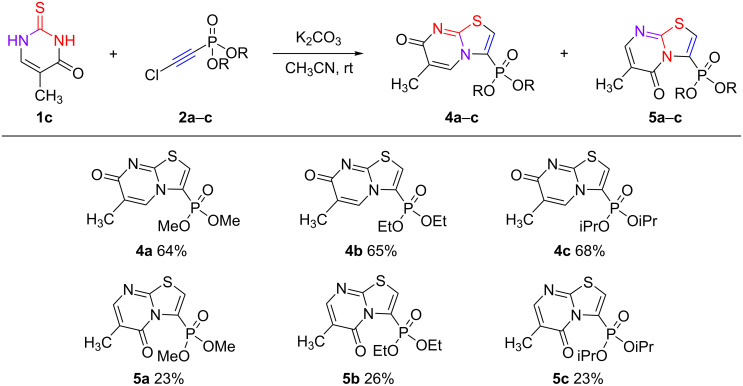
Reaction of 5-methyl-2-thiouracil (**1c**) with chloroethynylphosphonates **2a**–**c**.

The presence of the CH_3_ group at the position 5 of the thiouracil ring changes the reaction regioselectivity, as the main direction is cyclization through the N^1^ nitrogen atom with the formation of 3-phosphonylated thiazolo[3,2-*a*]-7-oxopyrimidines **4a**–**c** and 5-oxo regioisomers **5a**–**c** in a ≈1:0.1–0.3 ratio with yields of 87–91% (Scheme 7).

The structure of thiazolopyrimidines **4a**–**c** and **5a**–**с** is difficult to establish from the ^1^H and ^13^C NMR spectral data. The signals of the vinyl proton of the uracil moiety are represented by quartets (^4^*J*_HH_ = 1.4 Hz) in the δ_H_ 8.1 ppm region (quartet at δ_H_ 6 ppm for 6-methyl-5-oxo isomer). In the ^13^C NMR spectra, the signals of ethylene carbons of the uracil ring are presented at δ_C_ 131 and 122 ppm (δ_C_ 101 and 158 ppm for 5-oxopyrimidines **5a**–**c**). These data are in accordance with values of the chemical shifts of carbon atoms and protons of the pyrimidine ring in 3-methylthiazolo[3,2-*a*]pyrimidine-7-one [29], 3-phenylthiazolo[3,2-*a*]pyrimidine-7-one [40], and 5-phenylthiazolo[3,2-*a*]pyrimidine-7-one [16].

**Scheme 8:**
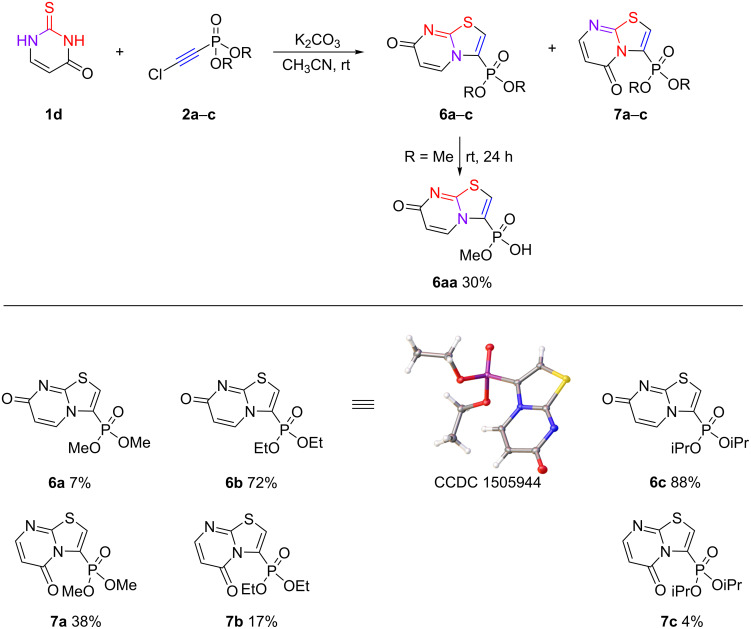
Reaction of 2-thiouracil (**1d**) with chloroethynylphosphonates **2a**–**c**.

A similar reaction outcome was observed when chloroethynylphosphonates **2a**–**c** were reacted with unsubstituted 2-thiouracil (**1d**). The cyclization reaction also proceeded predominantly through the N^1^ atom to form the corresponding 3-phosphonylated thiazolo[3,2-*a*]-7-oxopyrimidines **6a**–**c** together with a small amount of 5-oxo isomers **7a**–**c** (Scheme 8). In addition, it should be noted that the regioselectivity of the reaction was higher when using diisopropyl 2-chloroetynylphosphonate (**2c**).

The assignment of the major reaction product to the 7-oxo isomers was made by help of ^13^C NMR spectroscopy. In the ^13^C NMR spectra of the thiazolopyrimidines **6a**–**c**, the O=C–*CH=CH* fragment is observed by signals of equal intensity at δ_C_ 112–113 and 135–136 ppm. However, the unambiguous proof of the structure of thiazolopyrimidine-7-one **6b** was obtained by single crystal X-ray diffraction analysis.

It is important to note, that the reaction of dimethyl 2-chloroethynylphosphonate (**2a**) with 2-thiouracil had some features. The reaction proceeded with the formation of a mixture of 7-oxo and 5-oxo isomers in a ≈1: 0.1–0.3 ratio. However, a decrease in the signal of the 7-oxo isomer was observed in the ^31^P NMR spectrum upon standing at room temperature for 24 hours. The analysis of the formed precipitate identified product **6aa**, representing the monodealkylation product of the dimethylphosphonate group. A similar phenomenon has been reported earlier [41].

**Scheme 9:**
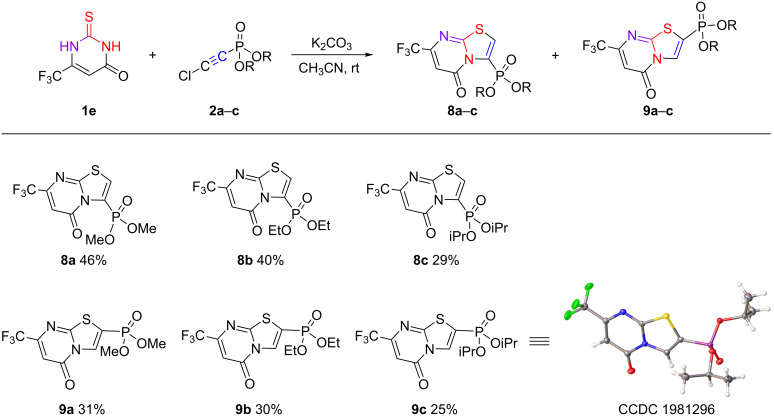
Reaction of 6-trifluoromethyl-2-thiouracil (**1e**) with chloroethynylphosphonates **2a**–**c**.

In the case of 6-trifluoromethyl-2-thiouracil (**1e**), a dramatic change in the reaction regioselectivity was observed. The cyclization took place with the formation of a mixture of the corresponding 2- and 3-phosphonylated thiazolopyrimidine-5-ones **8a**–**c** and **9a**–**c** (Scheme 9). As in the case of the 6-substituted 2-thiouracils **1a** and **1b**, the presence of a trifluoromethyl group in position 6 favored an attack by the N^3^-nitrogen atom, resulting in the formation of the 5-oxo isomer. The formation of the 2-phosphonylated thiazolopyrimidine-5-one could be explained by a primary attack of the electrophilic carbon atom bonded to the chlorine atom by the N^3^ nitrogen atom of the uracil fragment followed by cyclization.

The mixture of phosphonylated 5-oxothiazolopyrimidines **8c** and **9c** was separated by column chromatography and the ^1^Н, ^13^С, and ^31^Р NMR spectral data of the individual thiazolopyrimidine isomers did not allow convincing assignment. In the ^1^H NMR spectra, the proton of the thiazole ring of both isomers is represented by a doublet signal in the low field region at δ_H_ 7.91 (^3^*J*_HP_ = 3.2 Hz) and 8.41 ppm (^3^*J*_HP_ = 7.5 Hz), whereas the corresponding proton of the uracil ring resonated as a singlet at δ_H_ 6.61 and 6.71 ppm, respectively. Note that in the case of the reported 3-(4-bromophenyl)-7-trifluoromethyl-5*H*-[1,3]thiazolo[3,2-*a*]pyrimidine-5-one [18] the signal of the uracil proton H^6^ appeared at δ_H_ 6.67 ppm.

The ^13^C NMR spectra of the isomers **8c** and **9c** differed only in the values of the carbon signals at the phosphorus atom, i.e., doublets at 119.78 (^1^*J*_HP_ = 209.5 Hz) and 130.24 ppm (^1^*J*_HP_ = 217.5 Hz). The remaining signals were completely identical. Finally, the X-ray diffraction analysis of the 2-phosphonylated thiazolopyrimidine **9c** allowed us to uniquely determine its structure.

**Scheme 10:**
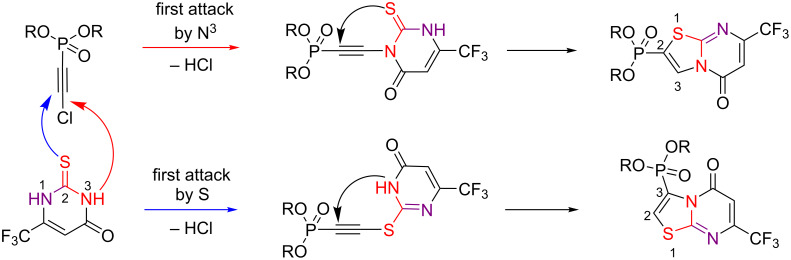
A plausible mechanism of the reaction between 6-trifluoromethyl-2-thiouracil (**1e**) and chloroethynylphosphonates.

In our opinion, the unusual formation of the 2-phosphonylated thiazolopyrimidine can be explained by the electron-withdrawing effect of the trifluoromethyl group in the starting 2-thiouracil. In contrast to 6-methyl- or 6-phenyl-2-thiouracil, where the nucleophilicity is localized on the sulfur atom, the presence of the electron-withdrawing CF_3_ group in 6-trifluoromethyl-2-thiouracil (**1e**) enhances the acidity of the N^3^H hydrogen by direct conjugation to the carbonyl moiety. As a result, 2-thiouracil **1e** acts as an ambident nucleophile. Thus, the attack of the carbon atom attached to the chlorine by the N^3^ nitrogen atom is accompanied by the elimination of hydrogen chloride (Scheme 10). A further 5-*endo-dig*-type cyclization results in the formation of the 2-phosphonylated 5-oxopyrimidines **9a**–**c**. The formation of the 3-phosphonylated 5-oxopyrimidines **8a**–**c** is due to the implementation of the usual favorable direction with the attack of chloroethynylphosphonate by the sulfur atom [33,42–46].

## Conclusion

In conclusion, a series of phosphonylated thiazolo[3,2-*a*]oxopyrimidines was first synthesized by reacting unsubstituted and substituted 2-thiouracils with chloroethynylphosphonates. The main regularities of this reaction were revealed. In the case of 6-substituted 2-thiouracil the primary attack by the most favorable nucleophilic site C=S takes place with further cyclization through the N^3^ atom of 2-thiouracil to form 5-oxopyrimidines. When using unsubstituted and 5-substituted 2-thiouracils, cyclization occurs predominantly through the N^1^ atom of the uracil ring, leading to the formation of 7-oxopyrimidines.

## Supporting Information

File 1 General experimental procedure, characterization data, and copies of NMR spectra.
