# Supplementary material for: Synthesis of 3(2)-phosphonylated thiazolo[3,2-a]oxopyrimidines
Source: Beilstein J Org Chem. 2020 Aug 10;16:1947–54. doi: 10.3762/bjoc.16.161 (PMC7431766; doi:10.3762/bjoc.16.161)
Supplement: File 1 — General experimental procedure, characterization data, and copies of NMR spectra. [file Beilstein_J_Org_Chem-16-1947-s001.pdf]

BEILSTEIN JOURNAL OF ORGANIC CHEMISTRY

Supporting Information

for

Synthesis of 3(2)-phosphonylated thiazolo[3,2-*a*]oxopyrimidines

Ksenia I. Kaskevich, Anastasia A. Babushkina, Vladislav V. Gurzhiy, Dmitrij M. Egorov, Nataly I. Svintsitskaya and Albina V. Dogadina

Beilstein J. Org. Chem. **2020,** *16,* 1947–1954. doi:10.3762/bjoc.16.161

General experimental procedure, characterization data, and copies of NMR spectra

License and Terms: This is a supporting information file under the terms of the Creative Commons Attribution License (http://creativecommons.org/licenses/by/4.0). Please note that the reuse, redistribution and reproduction in particular requires that the authors and source are credited.
The license is subject to the *Beilstein Journal of Organic Chemistry* terms and conditions: (https://www.beilstein-journals.org/bjoc)

Table of contents

A. General methods S2

B. Procedure for the preparation of 3–9 .... S2

C. Analytical data S3

D. NMR spectra S9

A. **General Methods**

All reactions were carried out under an inert atmosphere of argon in oven-dried glassware with magnetic stirring, unless otherwise noted. All chemicals were purchased from commercial suppliers and used without further purification. Column chromatography purifications were performed using Merck silica gel 60. Commercial grade solvents and reagents were used without further purification. Analytical thin layer chromatography (TLC) was performed using Merck 60 F254 silica gel plates. Subsequent to elution, plates were visualized using UV irradiation (254 nm). Melting points are uncorrected and were recorded on a Kofler hot-stage (VEB Wägetechnik Rapido, PHMK 81/2969). ^{1}H, ^{13}C, ^{19}F, ^{15}N and ^{31}P NMR spectra were registered at 400, 101, 376, 41, and 162 MHz, respectively, using a Bruker Avance 400 spectrometer. Residual solvent peaks were used as reference. IR spectra were registered on an IRPrestige-21 (Shimadzu) instrument from KBr pellets. HRMS (ESI) analyses were performed on a Bruker micrOTOF mass spectrometer. The X-ray crystal structures were detected on single crystal diffractometers Agilent Technologies (Oxford Diffraction) «Supernova» and «Xcalibur».

B. **Typical experimental procedure forthe synthesis of compounds 3–9.** A mixture of 0.001 mol of 2-chloroethynylphosphonate **2a–c**, 0.001 mol of 2-thiouracil **1a–e**, 10 mL of anhydrous acetonitrile, and 0.0012 mol of potassium carbonate was vigorously stirred at room temperature for 3–5 h. After completion of the reaction, the precipitate was filtered off and washed with ethanol. The filtrate was evaporated in vacuum. The title compounds were isolated by column chromatography (eluent ethyl acetate/petroleum ether 40:70), and then recrystallized from ethyl acetate.

C. Analytical data

Table S1 NMR spectral data of compounds **3–8**

Entry	Product	^{1}H NMR, δ, ppm	^{13}C NMR, δ, ppm			^{31}P NMR,δ, ppm
		H2	C2	C3	C9	
**3a**	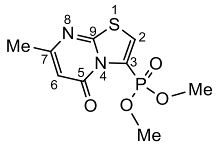	7.70 d ($^{3}J_{HP}$=7.6 Hz)	125.99 d ($^{2}J_{CP}$=13.7Hz)	126.88 d ($^{1}J_{CP}$= 225.9 Hz)	162.62 d ($^{3}J_{CP}$= 10.3 Hz)	3.75
**3b**	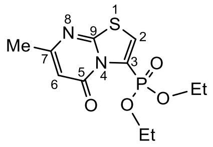	7.69 d ($^{3}J_{HP}$=7.6 Hz)	125.28 d ($^{2}J_{CP}$=13.6Hz)	128.22 d ($^{1}J_{CP}$= 223.2 Hz)	162.81 d ($^{3}J_{CP}$= 9.7 Hz)	0.93
**3c**	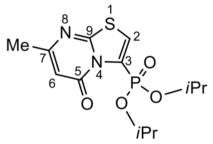	7.71 d ($^{3}J_{HP}$=8.0 Hz)	125.02 d ($^{2}J_{CP}$=13.6Hz)	129.64 d ($^{1}J_{CP}$= 221.3 Hz)	163.09 d ($^{3}J_{CP}$= 10.1 Hz)	-1.28
**3d**	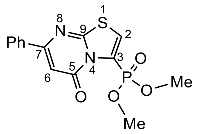	7.80 d ($^{3}J_{HP}$=6.6 Hz)	126.32 d ($^{2}J_{CP}$=13.6Hz)	127.12 d ($^{1}J_{CP}$= 225.9 Hz)	163.03 d ($^{3}J_{CP}$= 10.3 Hz)	3.84
**3e**	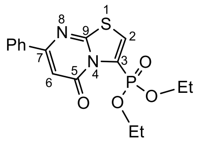	7.78 d ($^{3}J_{HP}$=7.7 Hz)	125.91 d ($^{2}J_{CP}$=10.2Hz)	128.16 d ($^{1}J_{CP}$= 223.7 Hz)	163.21 d ($^{3}J_{CP}$= 10.2 Hz)	1.02
**3f**	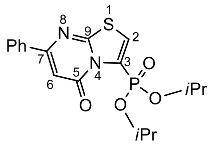	7.78 d ($^{3}J_{HP}$=7.7 Hz)	125.51 d ($^{2}J_{CP}$= 9.7Hz)	129.62 d ($^{1}J_{CP}$= 221.3 Hz)	163. 48d ($^{3}J_{CP}$= 9.7 Hz)	-1.21
**4a**	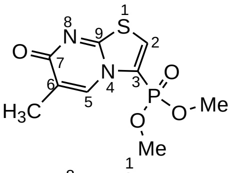	7.60 d ($^{3}J_{HP}$=7.9 Hz)	123.40 d ($^{2}J_{CP}$= 16.0 Hz)	125.39 d ($^{1}J_{CP}$= 215.0 Hz)	164. 31d ($^{3}J_{CP}$= 11.1 Hz)	3.55
**4b**	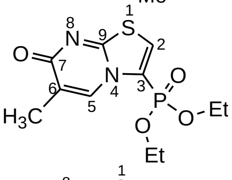	7.58 d ($^{3}J_{HP}$=7.8 Hz)	122.80 d ($^{2}J_{CP}$= 16.0 Hz)	126.57 d ($^{1}J_{CP}$= 213.4 Hz)	164. 42d ($^{3}J_{CP}$= 11.0 Hz)	0.5
**4c**	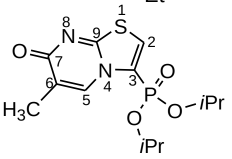	7.53 d ($^{3}J_{HP}$=7.8 Hz)	122.06 d ($^{2}J_{CP}$= 15.9 Hz)	127.87 d ($^{1}J_{CP}$= 213.0 Hz)	164. 47d ($^{3}J_{CP}$= 10.9 Hz)	-2.01
**6aa**	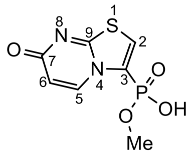	8.63 d ($^{3}J_{HP}$=7.7 Hz)	122.75 (d, $^{2}J_{CP}$ = 14.0 Hz)	131.95 d ($^{1}J_{CP}$ = 188.0 Hz)	167.42 d ($^{3}J_{CP}$ = 9.3 Hz)	-2.46

**6a**	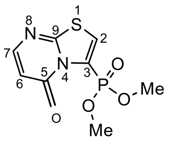	8.17 d ($^3J_{HP}$=7.8 Hz)	-	-	-	3.68
**6b**	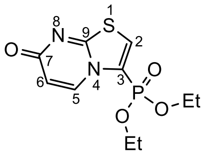	7.66 d ($^3J_{HP}$=7.9 Hz)	123.48 d ($^2J_{CP}$= 16.0 Hz)	126.67 d ($^1J_{CP}$= 213.5 Hz)	165. 49d ($^3J_{CP}$= 10.4 Hz)	0.19
**6c**	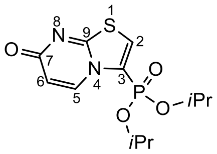	7.58 d ($^3J_{HP}$=7.9 Hz)	122.61 d ($^2J_{CP}$= 16.1 Hz)	128.08 d ($^1J_{CP}$= 212.7 Hz)	165. 53d ($^3J_{CP}$= 10.7 Hz)	-2.27
**6d**	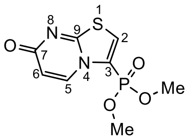	7.83 d ($^3J_{HP}$=7.7 Hz)	-	-	-	3.06
**8a**	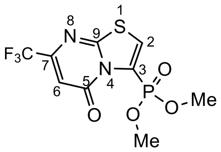	7.94 d ($^3J_{HP}$=7.5 Hz)	127.56 d ($^2J_{CP}$= 13.3 Hz)	127.90 d ($^1J_{CP}$= 223.8 Hz)		3.06
**8b**	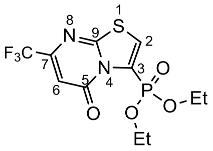	7.91 d ($^3J_{HP}$=7.5 Hz)	127.21 d ($^2J_{CP}$= 13.2 Hz)	128.89 d ($^1J_{CP}$= 221.5 Hz)		0.25
**8c**	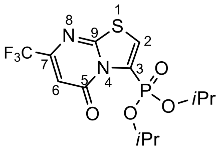	7.91 d ($^3J_{HP}$=7.6 Hz)	126.94 d ($^2J_{CP}$= 13.6 Hz)	130.24 d ($^1J_{CP}$= 219.5 Hz)		-1.96
**8d**	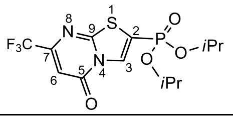	8.41 d ($^3J_{HP}$=7.6 Hz)	128.49 d ($^2J_{CP}$= 17.0 Hz)	119.79 d ($^1J_{CP}$= 209.3 Hz)		2.45

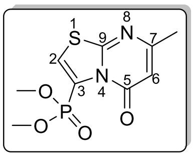

Dimethyl (7-methyl-5-oxo-5*H*-[1,3]thiazolo[3,2-*a*]pyrimidin-3-yl)phosphonate (3a). Yield 83%, R_f 0.39 (EtOAc–hexane, 1:2), white solid, mp 118–120°C. **^{1}H NMR** (400 MHz, $CDCl_3$) δ 7.70 (d, $^3J_{HP}$ = 7.6 Hz, 1H, H2), 6.02 (q, $^4J_{HH}$ = 0.8 Hz, 1H, H6), 3.82 (d, $^3J_{HP}$ = 11.6 Hz, 6H, O$C\underline{H}_3$), 2.21 (s, 3H, $C\underline{H}_3$). **^{13}C NMR** (101 MHz, $CDCl_3$) δ 163.76 (C5), 162.62 (d, $^3J_{CP}$ = 10.3 Hz, C9), 158.19 (C7), 126.88 (d, $^1J_{CP}$ = 225.9 Hz, C3), 125.99 (d, $^2J_{CP}$ = 13.7 Hz, C2), 104.93 (C6), 54.33 (d, $^2J_{CP}$ = 5.9 Hz, O$\underline{C}H_3$), 23.68 ($\underline{C}H_3$) **^{31}P NMR** (162 MHz, $CDCl_3$) δ 3.75. ν_{max}(KBr)/cm^{-1} 3127, 2961, 2857, 1674, 1566, 1531, 1484, 1397, 1362, 1289, 1246, 1126, 1065, 1023, 965, 867, 819, 761, 754, 659, 624, 570. HRMS-ESI (m/z): calcd for $C_9H_{11}N_2O_4PS$, $[M+H]^+$: 275.0250, found 275.0259.

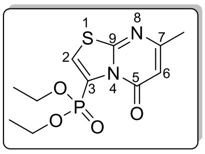

Diethyl (7-methyl-5-oxo-5*H*-[1,3]thiazolo[3,2-*a*]pyrimidin-3-yl)phosphonate (3b). Yield 80%, R_f 0.35 (EtOAc–hexane, 1:2), pale yellow solid, mp 108–110°C. **^{1}H NMR** (400 MHz, $CDCl_3$) δ 7.69 (d, $^3J_{HP}$ = 7.6 Hz, 1H, H2), 6.09 (q, $^4J_{HH}$ = 0.9 Hz, 1H, H6), 4.43 – 4.07 (m, $^3J_{CP}$ = 11.9, $^3J_{HH}$ = 7.1 Hz, 4H, O$C\underline{H}_2$), 2.27 (d, $^4J_{HH}$ = 0.8 Hz, 3H, $C\underline{H}_3$), 1.32 (t, $^3J_{HH}$ = 7.1 Hz, 6H, $OCH_2C\underline{H}_3$). **^{13}C NMR** (101 MHz, $CDCl_3$) δ 163.59 (C5), 162.81 (d, $^3J_{CP}$ = 9.7 Hz, C9), 158.26 (C6), 128.22 (d, $^1J_{CP}$ = 223.2 Hz, C3), 125.28 (d, $^2J_{CP}$ = 13.6 Hz, C2), 105.11 (C7), 63.96 (d, $^2J_{CP}$ = 6.1 Hz, O$\underline{C}H_2$), 23.70 ($\underline{C}H_3$), 16.39 (d, $^3J_{CP}$ = 6.5 Hz, $OCH_2\underline{C}H_3$). **^{15}N NMR** (41 MHz, $CDCl_3$) δ 227.48 (N8), 203.32 (d, $^2J_{NP}$ = 10.1 Hz, N4). **^{31}P NMR** (162 MHz, $CDCl_3$) δ 0.93. ν_{max}(KBr)/cm^{-1} 3219, 2992, 2000, 1682, 1563, 1528, 1491, 1399, 1364, 1287, 1244, 1142, 1021, 967, 918, 880, 821, 786, 743, 696, 623, 561. HRMS-ESI (m/z): calcd for $C_{11}H_{15}N_2O_4PS$, $[M+H]^+$: 303.0563, found 303.0575.

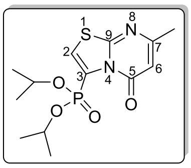

Diisopropyl (7-methyl-5-oxo-5*H*-[1,3]thiazolo[3,2-*a*]pyrimidin-3-yl)phosphonate (3c). Yield 79%, R_f 0.38 (EtOAc–hexane, 1:2), yellow solid, mp 116–118°C. **^{1}H NMR** (400 MHz, $CDCl_3$) δ 7.71 (d, $^3J_{HP}$ = 8.0 Hz, 1H, H2), 6.12 (q, $^4J_{HH}$ = 2.3 Hz, 1H, H6), 5.07 – 4.91 (m, $^3J_{CP}$ = 7.9, $^3J_{HH}$ = 6.2 Hz, 2H, OC$\underline{H}$), 2.30 (d, $^4J_{HH}$ = 2.3 Hz, 3H, $C\underline{H}_3$), 1.38 (d, $^3J_{HH}$ = 5.0 Hz, 6H, $OCH(C\underline{H}_3)_2$), 1.31 (d, $^3J_{HH}$ = 6.1 Hz, 6H, $OCH(C\underline{H}_3)_2$). **^{13}C NMR** (101 MHz, $CDCl_3$) δ 163.34 (C5), 163.09 (d, $^3J_{CP}$ = 10.1 Hz, C9), 158.19 (C6), 129.64 (d, $^1J_{CP}$ = 221.3 Hz, C3), 125.02 (d, $^2J_{CP}$ = 13.6 Hz, C2), 105.25 (C7), 72.91 (d, $^2J_{CP}$ = 6.1 Hz, O$\underline{C}$H), 24.20 (d, $^3J_{CP}$ = 3.9 Hz, $OCH_2\underline{C}H_3$), 23.81 (d, $^3J_{CP}$ = 5.8 Hz, $OCH_2\underline{C}H_3$), 23.68 ($\underline{C}H_3$). **^{31}P NMR** (162 MHz, $CDCl_3$) δ -1.28. ν_{max}(KBr)/cm^{-1} 3135, 2985, 1687, 1569, 1530, 1487, 1387, 1369, 1279, 1261, 1180, 1126, 1021, 987, 864, 776, 683, 621, 565, 554. HRMS-ESI (m/z): calcd for $C_{13}H_{19}N_2O_4PS$, $[M+H]^+$: 353.0695, found 353.0706.

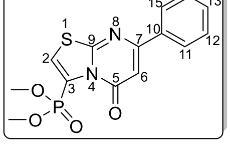

Dimethyl (5-oxo-7-phenyl-5*H*-[1,3]thiazolo[3,2-*a*]pyrimidin-3-yl)phosphonate (3d). Yield 89%, R_f 0.37 (EtOAc–hexane, 1:2), pale yellow solid, mp 173–175°C. **^{1}H NMR** (400 MHz, $CDCl_3$) δ 7.98 – 7.95 (m, 2H, H12, H14), 7.80 (d, $^3J_{HP}$ = 6.6 Hz, 1H, H2),7.49 – 7.39 (m, 3H, H11,H13,H15), 6.74 (s, 1H, H6), 3.98 (d, $^3J_{HP}$ = 11.6 Hz, 6H, O$C\underline{H}_3$). **^{13}C NMR** (101 MHz, $CDCl_3$) δ 163.03 (d, $^3J_{CP}$ = 10.3 Hz, C9), 160.93 (C5), 158.95 (C7), 135.75 (C10), 130.93 (C13), 128.84 (C_{Ph}), 127.28 (C_{Ph}), 127.12 (d, $^1J_{CP}$ = 225.9 Hz, C3), 126.32 (d, $^2J_{CP}$ = 13.6 Hz, C2), 101.77 (C6), 54.52 (d, $^2J_{CP}$ = 6.4 Hz, O$\underline{C}H_3$). **^{15}N NMR** (41 MHz, $CDCl_3$) δ 221.57 (N8), 203.25 (d, $^2J_{NP}$ = 6.8 Hz, N4). **^{31}P NMR** (162 MHz, $CDCl_3$) δ 3.84. ν_{max}(KBr)/cm^{-1} 3126, 2954, 2850, 1673, 1558, 1532, 1499, 1479, 1385, 1292, 1248, 1133, 1065, 1029, 976, 842, 827, 765, 753, 694, 650, 576. HRMS-ESI (m/z): calcd for $C_{14}H_{13}N_2O_4PS$, $[M+H]^+$: 337.0406, found 337.0411.

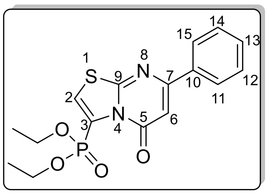

Diethyl (5-oxo-7-phenyl-5*H*-[1,3]thiazolo[3,2-*a*]pyrimidin-3-yl)phosphonate (3e). Yield 76%, R_f 0.35 (EtOAc–hexane, 1:2), pale yellow solid, mp 152–154°C. **^{1}H NMR** (400 MHz, $CDCl_3$) δ 8.03 – 7.89 (m, 2H, H12, H14), 7.78 (d, $^{3}J_{HP}$ = 7.7 Hz, 1H, H2), 7.51 – 7.39 (m, 3H, H11,H13,H15), 6.72 (s, 1H, H6), 4.51 – 4.21 (m, $^{3}J_{CP}$ = 11.8, $^{3}J_{HH}$ = 7.1 Hz, 4H, $OC\underline{H}_2$), 1.39 (t, $^{3}J_{HH}$ = 7.1 Hz, 6H, $C\underline{H}_3$). **^{13}C NMR** (101 MHz, $CDCl_3$) δ 163.21 (d, $^{3}J_{CP}$ = 10.2 Hz, C9), 160.74 (C5), 158.95 (C7), 135.78 (C10), 130.85 (C_{Ph}), 128.81(C_{Ph}), 128.16 (d, $^{1}J_{CP}$ = 223.7 Hz, C3), 127.24 (C_{Ph}), 125.91 (d, $^{2}J_{CP}$ = 13.3 Hz, C2), 101.80 (C6), 64.13 (d, $^{2}J_{CP}$ = 6.4 Hz, $O\underline{C}H_2$), 16.47 (d, $^{3}J_{CP}$ = 6.5 Hz, $\underline{C}H_3$). **^{31}P NMR** (162 MHz, $CDCl_3$) δ 1.02. ν_{max}(KBr)/cm^{-1} 3134, 2981, 2907, 1685, 1553, 1505, 1481, 1378, 1290, 1251, 1130, 1056, 1019, 976, 838, 774, 692, 654, 566, 528. HRMS-ESI (m/z): calcd for $C_{16}H_{17}N_2O_4PS$, $[M+H]^+$: 387.0539, found 387.0552.

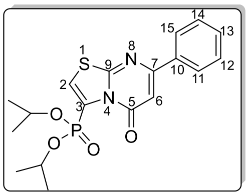

Diisopropyl (5-oxo-7-phenyl-5*H*-[1,3]thiazolo[3,2-*a*]pyrimidin-3-yl)phosphonate (3f). Yield 87%, R_f 0.34 (EtOAc–hexane, 1:2), pale yellow solid, mp 156–158°C. **^{1}H NMR** (400 MHz, $CDCl_3$) δ 8.01 – 7.95 (m, 2H, H12, H14), 7.78 (d, $^{3}J_{HP}$= 7.7 Hz, 1H, H2), 7.56 – 7.39 (m, 3H, H11,H13,H15), 6.73 (s, 1H, H6), 5.17 – 4.96 (m, $^{3}J_{CP}$ = 7.7, $^{3}J_{HH}$ = 6.2 Hz, 2H, $OC\underline{H}$), 1.43 (d, $^{3}J_{HH}$ = 6.2 Hz, 6H, $C\underline{H}_3)_2$), 1.37 (d, $^{3}J_{HH}$ = 6.2 Hz, 6H, $(C\underline{H}_3)_2$). **^{13}C NMR** (101 MHz, $CDCl_3$) δ 163.48 (d, $^{3}J_{CP}$ = 9.7 Hz, C9), 160.50 (C5), 158.83 (C7), 135.86 (C_{Ph}), 130.79 (C_{Ph}), 129.62 (d, $^{1}J_{CP}$ = 221.3 Hz,C3), 128.81 (C_{Ph}), 125.51 (d, $^{2}J_{CP}$ = 13.6 Hz, C2), 101.95 (C6), 73.01 (d, $^{2}J_{CP}$ = 6.5 Hz, $O\underline{C}H$), 24.26 (d, $^{3}J_{CP}$ = 3.8 Hz, $\underline{C}H_3$), 23.88 (d, $^{3}J_{CP}$ = 5.8 Hz, $\underline{C}H_3$). **^{31}P NMR** (162 MHz, $CDCl_3$) δ -1.21. ν_{max}(KBr)/cm^{-1} 3446, 3128, 2976, 1687, 1559, 1527, 1503, 1480, 1385, 1247, 1140, 1111, 1071, 1017, 982, 842, 774, 744, 694, 654, 582. HRMS-ESI (m/z): calcd for $C_{18}H_{21}N_2O_4PS$, $[M+H]^+$: 415.0852, found 415.0867.

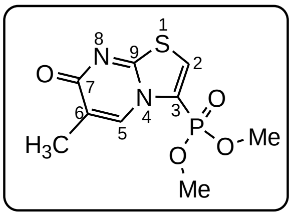

Dimethyl (6-methyl-7-oxo-5*H*-[1,3]thiazolo[3,2-*a*]pyrimidin-3-yl)phosphonate (4a). Yield 64%, R_f 0.57 (EtOAc–hexane, 1:2), yellow solid, mp 139–141°C. **^{1}H NMR** (400 MHz, $CDCl_3$) δ 8.09 (q, $^{4}J_{HH}$ = 1.3 Hz, 1H, H7), 7.60 (d, $^{3}J_{HP}$ = 7.9 Hz, 1H, H2), 3.92 (d, $^{3}J_{HP}$ = 11.6 Hz, 6H, $OC\underline{H}_3$), 2.14 (d, $^{4}J_{HH}$ = 1.3 Hz, 3H, $C\underline{H}_3$). **^{13}C NMR** (101 MHz, $CDCl_3$) δ 167.32 (C5), 164.31 (d, $^{3}J_{CP}$ = 11.1 Hz, C9), 131.37 (C6), 125.39 (d, $^{1}J_{CP}$ = 215.0 Hz,C3), 123.40 (d, $^{2}J_{CP}$ = 16.0 Hz, C2), 122.21(C7), 54.17 (d, $^{2}J_{CP}$ = 5.8 Hz, $O\underline{C}H_3$), 14.86 (CH_3). **^{31}P NMR** (162 MHz, $CDCl_3$) δ 3.55. ν_{max}(KBr)/cm^{-1} 3445, 3052, 2958, 2854, 1644, 1691, 1545, 1495, 1419, 1380, 1344, 1265, 1185, 1146, 1041, 972, 838, 774, 757, 539. HRMS-ESI (m/z): calcd for $C_9H_{11}N_2O_4PS$, $[M+H]^+$: 297.0069, found 297.0079.

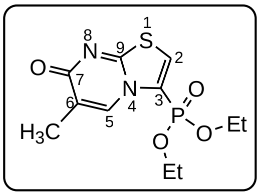

Diethyl (6-methyl-7-oxo-5*H*-[1,3]thiazolo[3,2-*a*]pyrimidin-3-yl)phosphonate (4b). Yield 65%, R_f 0.54 (EtOAc–hexane, 1:2), pale yellow solid, mp 141–143°C. **^{1}H NMR** (400 MHz, $CDCl_3$) δ 8.10 (q, $^{4}J_{HH}$ = 1.4 Hz, 1H, H7), 7.58 (d, $^{3}J_{HP}$ = 7.8 Hz, 1H, H2), 4.47 – 4.05 (m, $^{3}J_{CP}$ = 10.2, $^{3}J_{HH}$ = 7.1 Hz, 4H, $OC\underline{H}_2$), 2.08 (d, $^{4}J_{HH}$ = 1.4 Hz, 3H, $C\underline{H}_3$), 1.36 (t, $^{3}J_{HH}$ = 7.1 Hz, 6H, $OCH_2C\underline{H}_3$). **^{13}C NMR** (101 MHz, $CDCl_3$) δ 167.38 (C5), 164.42 (d, $^{3}J_{CP}$ = 11.0 Hz, C9), 131.45 (C6), 126.57 (d, $^{1}J_{CP}$ = 213.4 Hz, C3), 122.80 (d, $^{2}J_{CP}$ = 16.0 Hz, C2), 121.93 (C7), 64.31 (m, $^{2}J_{CP}$ = 5.7 Hz, $O\underline{C}H_2$), 16.28 (d, $^{3}J_{CP}$ = 6.0 Hz, $OCH_2\underline{C}H_3$), 14.81($\underline{C}H_3$). **^{31}P NMR** (162 MHz, $CDCl_3$) δ 0.50. ν_{max}(KBr)/cm^{-1} 3463, 3085, 2981, 2923, 1647, 1623, 1547, 1498, 1419, 1400, 1344, 1252, 1144, 1046, 1016, 978, 847, 772, 613, 567. HRMS-ESI (m/z): calcd for $C_{11}H_{15}N_2O_4PS$, $[M+H]^+$: 325.0382, found 325.0392.

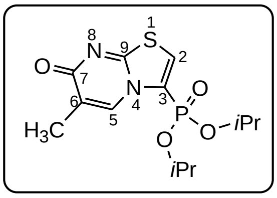

Diisopropyl (6-methyl-7-oxo-5*H*-[1,3]thiazolo[3,2-*a*]pyrimidin-3-yl)phosphonate (4c). Yield 68%, R_f 0.54 (EtOAc–hexane, 1:2), pale yellow solid, mp 130–132°C. **^{1}H NMR** (400 MHz, $CDCl_3$) δ 8.12 (q, $^4J_{HH}$ = 1.4 Hz, 1H, H7), 7.53 (d, $^3J_{HP}$ = 7.8 Hz, 1H, H2), 5.09 – 4.60 (m, $^3J_{HP}$ = 7.8, $^3J_{HH}$ = 6.2 Hz, 2H, $OC\underline{H}(CH_3)_2$), 2.11 (d, $^4J_{HH}$ = 1.4 Hz, 3H, $C\underline{H}_3$), 1.43 (d, $^3J_{HH}$ = 6.2 Hz, 6H, $OCH(C\underline{H}_3)_2$), 1.31 (d, $^3J_{HH}$ = 6.2 Hz, 6H, $OCH(C\underline{H}_3)_2$). **^{13}C NMR** (101 MHz, $CDCl_3$) δ 167.41(C5), 164.47 (d, $^3J_{CP}$ = 10.9 Hz, C9), 131.42 (C6), 127.87 (d, $^1J_{CP}$ = 213.0 Hz, C3), 122.06 (d, $^2J_{CP}$ = 15.9 Hz,C2), 121.87 (C7), 73.88 (d, $^2J_{CP}$ = 5.8 Hz, $O\underline{C}H$), 24.02 (d, $^3J_{CP}$ = 4.1 Hz, $OCH(\underline{C}H_3)_2$), 23.73 (d, $^3J_{CP}$ = 5.1 Hz, $OCH(\underline{C}H_3)_2$), 14.85($\underline{C}H_3$). **^{31}P NMR** (162 MHz, $CDCl_3$) δ -2.01. ν_{max}(KBr)/cm^{-1} 3109, 2980, 2924, 1623, 1536, 1481, 1419, 1387, 1344, 1265, 1138, 1021, 993, 849, 772, 614, 559, 514. HRMS-ESI (m/z): calcd for $C_{13}H_{19}N_2O_4PS$, $[M+H]^+$: 353.0695, found 353.0708.

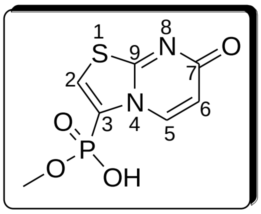

(7-Oxo-7*H*-thiazolo[3,2-*a*]pyrimidin-3-yl)phosphonic acid monomethyl ester (6aa). Yield 30%, pale yellow oil. **^{1}H NMR** (400 MHz, D_2O) δ 8.63 (d, $^3J_{HH}$ = 7.7 Hz, 1H, H5), 7.73 (d, $^3J_{HP}$ = 6.5 Hz, 1H, H2), 6.60 (d, $^3J_{HH}$ = 7.7 Hz, 1H, H6), 3.47 (d, $^3J_{HH}$ = 11. 6 Hz, 6H, $C\underline{H}_3$). **^{13}C NMR** (101 MHz, D_2O) δ 169.15 (C7), 167.42 (d, $^3J_{CP}$ = 9.3 Hz, C9), 139.27(C5), 131.95 (d, $^1J_{CP}$ = 188.0 Hz, C3), 122.75 (d, $^2J_{CP}$ = 14.0 Hz, C2), 111.01 (C6), 52.64 (d, $^2J_{CP}$ = 5.6 Hz, $O\underline{C}H_3$). **^{31}P NMR** (162 MHz, D_2O) δ -2.46.

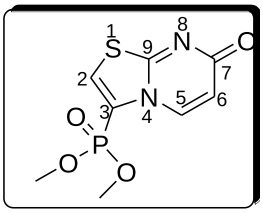

Dimethyl (7-oxo-7*H*-thiazolo[2,3-*b*]pyrimidin-3-yl)phosphonate (6a). Yield 7%, R_f 0.52 (EtOAc–hexane, 1:2), pale yellow oil. **^{1}H NMR** (400 MHz, $CDCl_3$) δ 8.17 (d, $^3J_{HH}$ = 7.8 Hz, 1H, H5), 7.69 (d, $^3J_{HP}$ = 7.7 Hz, 1H, H2), 6.35 (d, $^3J_{HH}$ = 7.8 Hz, 1H, H6), 3.87 (d, $^3J_{HP}$ = 11.6, 6H, OCH_3). **^{31}P NMR** (162 MHz, $CDCl_3$) δ 3.68.

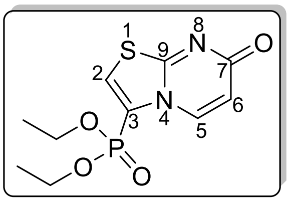

Diethyl (7-oxo-7*H*-thiazolo[3,2-*a*]pyrimidin-3-yl)phosphonate (6b). Yield 72%, R_f 0.52 (EtOAc–hexane, 1:2), pale yellow solid, mp 153–155°C. **^{1}H NMR** (400 MHz, $CDCl_3$) δ 8.19 (d, $^3J_{HH}$ = 7.8 Hz, 1H, H5), 7.66 (d, $^3J_{HP}$ = 7.9 Hz, 1H, H2), 6.33 (d, $^3J_{HH}$ = 7.8 Hz, 1H, H6), 4.40 – 4.07 (m, $^3J_{HP}$ = 10.1, $^3J_{HH}$ = 7.1 Hz, 4H, $OC\underline{H}_2CH_3$), 1.33 (t, $^3J_{HH}$ = 7.1 Hz, 6H, $OCH_2C\underline{H}_3$). **^{13}C NMR** (101 MHz, $CDCl_3$) δ 166.49 (C7), 165.49 (d, $^3J_{CP}$ = 10.4 Hz, C9), 135.33 (C5), 126.67 (d, $^1J_{CP}$ = 213.5 Hz, C3), 123.48 (d, $^2J_{CP}$ = 16.0 Hz, C2), 112.43(C6), 64.37 (d, $^2J_{CP}$ = 5.5 Hz, $O\underline{C}H_2$), 16.26 (d, $^3J_{CP}$ = 6.1 Hz, $OCH_2\underline{C}H_3$). **^{31}P NMR** (162 MHz, $CDCl_3$) δ 0.19. ν_{max}(KBr)/cm^{-1} 3037, 1637, 1550, 1481, 1428, 1351, 1262, 1161, 1083, 1038, 1028, 985, 834, 752, 591, 559. HRMS-ESI (m/z): calcd for $C_{10}H_{13}N_2O_4PS$, $[M+H]^+$: 289.0406, found 289.0413.

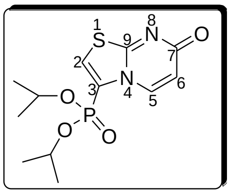

Diisopropyl (7-oxo-7*H*-[1,3]thiazolo[3,2-*a*]pyrimidin-3-yl)phosphonate (6c). Yield 88%, R_f 0.48 (EtOAc–hexane, 1:2), pale yellow solid, mp 97–99°C. **^{1}H NMR** (400 MHz, $CDCl_3$) δ 8.19 (d, $^3J_{HH}$ = 7.8 Hz, 1H, H5), 7.58 (d, $^3J_{HP}$ = 7.9 Hz, 1H, H2), 6.41 (d, $^3J_{HH}$ = 7.8 Hz, 1H, H6), 5.05 – 4.62 (m, $^3J_{HP}$ = 7.7 Hz, $^3J_{HH}$ = 6.2 Hz, 2H, $OC\underline{H}(CH_3)_2$), 1.44 (d, $^3J_{HH}$ = 6.2 Hz, 6H, $OCH(C\underline{H}_3)_2$), 1.31 (d, $^3J_{HH}$ = 6.2 Hz, 6H, $OCH(C\underline{H}_3)_2$). **^{13}C NMR** (101 MHz, $CDCl_3$) δ 166.55(C7), 165.53 (d, $^3J_{CP}$ = 10.7 Hz, C9), 135.21(C5), 128.08 (d, $^1J_{CP}$ = 212.7 Hz, C3),

122.61 (d, $^{2}J_{CP}$ = 16.1 Hz, C2), 112.46 (C6), 74.01 (d, $^{3}J_{CP}$ = 5.8 Hz, O$\underline{C}$H), 24.03 (d, $^{3}J_{CP}$ = 3.9 Hz, OCH($\underline{C}H_3)_2$), 23.74 (d, $^{3}J_{CP}$ = 4.7 Hz, OCH($\underline{C}H_3)_2$). **^{31}P NMR** (162 MHz, $CDCl_3$) δ -2.27. ν_{max}(KBr)/cm^{-1} 3541, 3493, 2989, 2000, 1643, 1542, 1481, 1429, 1398, 1351, 1260, 1161, 1083, 820, 776, 585, 563, 500. HRMS-ESI (m/z): calcd for $C_{12}H_{17}N_2O_4PS$, $[M+H]^+$: 317.0719, found 317.0730.

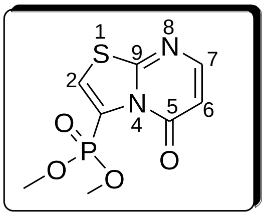

Dimethyl (5-oxo-7*H*-thiazolo[2,3-b]pyrimidin-3-yl)phosphonate (7a). Yield 38%, R_f 0.45 (EtOAc–hexane, 1:2), pale yellow oil. **^{1}H NMR** (400 MHz, $CDCl_3$) δ 7.95 (d, $^{3}J_{HH}$ = 6.5 Hz, 1H, H6), 7.83 (d, $^{3}J_{HP}$ = 7.7 Hz, 1H, H2), 6.33 (d, $^{3}J_{HH}$ = 6.5 Hz, 1H, H7), 3.75 (d, $^{3}J_{HP}$ = 11.1, 6H, OCH_3). **^{31}P NMR** (162 MHz, $CDCl_3$) δ 3.06.

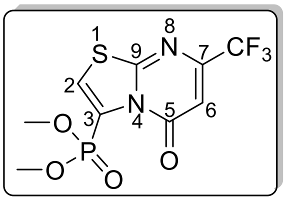

Dimethyl [5-oxo-7-(trifluoromethyl)-5*H*-[1,3]thiazolo[3,2-*a*]pyrimidin-3-yl]phosphonate (8a). Yield 46%, pale yellow solid, R_f 0.46 (EtOAc–hexane, 1:2), mp 170–174°C. **^{1}H NMR** (400 MHz, $CDCl_3$) δ 7.94 (d, $^{3}J_{HP}$ = 7.5 Hz, 1H, H2), 6.73 (s, 1H, H6), 4.00 (d, $^{3}J_{HP}$ = 11.6 Hz, 6H, OC$\underline{H}_3$). **^{13}C NMR** (101 MHz, $CDCl_3$) δ 164.75 (d, $^{3}J_{CP}$ = 10.3 Hz, C9), 157.77 (C5), 151.46 (q, $^{2}J_{CF}$ = 36.2 Hz, C7), 127.90 (d, $^{1}J_{CP}$ = 223.8 Hz, C3), 127.56 (d, $^{2}J_{CP}$ = 13.3 Hz, C2), 120.31 (q, $^{1}J_{CF}$ = 275.2 Hz, $\underline{C}F_3$), 104.72 (q, $^{3}J_{CF}$ = 3.1 Hz, C6), 54.62 (d, $^{2}J_{CP}$ = 6.2 Hz, O$\underline{C}H_3$). **^{19}F NMR** (376 MHz, $CDCl_3$) δ -70.49. **^{31}P NMR** (162 MHz, $CDCl_3$) δ 3.06. ν_{max}(KBr)/cm^{-1} 3132, 2963, 2370, 1692, 1533, 1501, 1427, 1281, 1250, 1192, 1155, 1136, 1067, 1032, 854, 822, 762, 717, 677, 571. HRMS-ESI (m/z): calcd for $C_9H_8F_3N_2O_4PS$, $[M+H]^+$: 328.9967, found 328.9962.

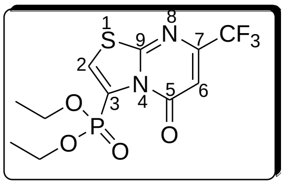

Diethyl [5-oxo-7-(trifluoromethyl)-5*H*-[1,3]thiazolo[3,2-*a*]pyrimidin-3-yl]phosphonate (8b). Yield 40%, R_f 0.43 (EtOAc–hexane, 1:2), pale yellow solid, mp 150–152°C. **^{1}H NMR** (400 MHz, $CDCl_3$) δ 7.91 (d, $^{3}J_{HP}$ = 7.5 Hz, 1H, H2), 6.67 (s, 1H, H6), 4.41 – 4.25 (m, $^{3}J_{HP}$ = 11.9, $^{3}J_{HH}$ = 7.1 Hz, 4H, OC$\underline{H}_2CH_3$), 1.38 (t, $^{3}J_{HH}$ = 7.1, Hz, 6H, $OCH_2C\underline{H}_3$). **^{13}C NMR** (101 MHz, $CDCl_3$) δ 164.98 (d, $^{3}J_{CP}$ = 10.2 Hz, C9), 157.73 (C5), 151.25 (q, $^{2}J_{CF}$ = 36.0 Hz, C7), 128.89 (d, $^{1}J_{CP}$ = 221.5 Hz, C3), 127.21 (d, $^{2}J_{CP}$ = 13.2 Hz, C2), 120.32 (q, $^{1}J_{CF}$ = 275.1 Hz, CF_3), 104.66 (q, $^{3}J_{CF}$ = 3.1 Hz, C6), 64.30 (d, $^{2}J_{CP}$ = 6.3 Hz, O$\underline{C}H_2$), 16.39 (d, $^{3}J_{CP}$ = 6.5 Hz, $OCH_2\underline{C}H_3$). **^{31}P NMR** (162 MHz, $CDCl_3$) δ 0.25. ν_{max}(KBr)/cm^{-1} 3132, 3071, 2990, 1705, 1535, 1501, 1427, 1283, 1250, 1188, 1144, 1061, 1028, 974, 852, 795, 719, 579. HRMS-ESI (m/z): calcd for $C_{11}H_{12}F_3N_2O_4PS$, $[M+H]^+$: 357.0280, found 357.0291.

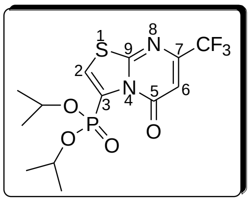

Diisopropyl [5-oxo-7-(trifluoromethyl)-5*H*-[1,3]thiazolo[3,2-*a*]pyrimidin-3-yl]phosphonate (8c). Yield 29%, R_f 0.42 (EtOAc–hexane, 1:2), pale yellow solid, mp 112–114°C. **^{1}H NMR** (400 MHz, $CDCl_3$) δ 7.91 (d, $^{3}J_{HP}$ = 7.6 Hz, 1H, H2), 6.67 (s, 1H, H6), 5.11 – 4.93 (m, $^{3}J_{HP}$ = 7.2, $^{3}J_{HH}$ = 6.2 Hz, 2H, OC$\underline{H}(CH_3)_2$), 1.42 (d, $^{3}J_{HH}$ = 6.2 Hz, 6H, OCH(C$\underline{H}_3)_2$), 1.34 (d, $^{3}J_{HH}$ = 6.2 Hz, 6H, OCH(C$\underline{H}_3)_2$). **^{13}C NMR** (101 MHz, $CDCl_3$) δ 165.21 (d, $^{3}J_{CP}$ = 10.0 Hz, C9), 157.58 (C5), 151.10 (q, $^{2}J_{CF}$ = 36.1 Hz, C7), 130.24 (d, $^{1}J_{CP}$ = 219.5 Hz, C3), 126.94 (d, $^{2}J_{CP}$ = 13.6 Hz, C2), 120.36 (q, $^{1}J_{CF}$ = 275.1 Hz, CF_3), 104.74 (q, $^{3}J_{CF}$ = 3.2 Hz, C6), 73.41 (d, $^{2}J_{CP}$ = 6.3 Hz, O$\underline{C}$H), 24.17 (d, $^{3}J_{CP}$ = 4.0 Hz, OCH($\underline{C}H_3$)), 23.81 (d, $^{3}J_{CP}$ = 5.6 Hz, OCH($\underline{C}H_3$)). **^{19}F NMR** (376 MHz, $CDCl_3$) δ -70.54. **^{31}P NMR** (162 MHz, $CDCl_3$) δ -1.96. ν_{max}(KBr)/cm^{-1} 3084, 2984, 2928, 2370, 1711, 1533, 1506, 1425, 1385, 1277, 1248, 1196, 1149, 1101, 1018, 993, 849, 742, 717, 673, 638, 582. HRMS-ESI (m/z): calcd for $C_{13}H_{16}F_3N_2O_4PS$, $[M+H]^+$: 385.0593, found 385.0606.

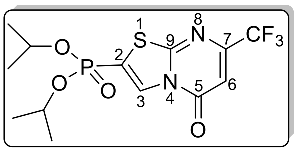

Diisopropyl [5-oxo-7-(trifluoromethyl)-5*H*-[1,3]thiazolo[3,2-*a*]pyrimidin-2-yl]phosphonate (9c). Yield 25%, R_f 0.54 (EtOAc–hexane, 1:2), pale yellow solid, mp 106–108°C. **^{1}H NMR** (400 MHz, $CDCl_3$) δ 8.41 (d, $^{3}J_{HP}$ = 7.5 Hz, 1H, H3), 6.71 (s, 1H, H6), 4.84 (m, $^{3}J_{HP}$ = 7.2, $^{3}J_{HH}$ = 6.2 Hz, 2H, OC<u>H</u>(CH_3)$_2$), 1.44 (d, $^{3}J_{HH}$ = 6.2 Hz, 6H, OCH(C<u>H</u>$_3$)$_2$), 1.34 (d, $^{3}J_{HH}$ = 6.2 Hz, 6H, OCH(C<u>H</u>$_3$)$_2$). **^{13}C NMR** (101 MHz, $CDCl_3$) δ 165.31 (d, $^{3}J_{CP}$ = 10.4 Hz, C9), 157.51 (C5), 152.24 (q, $^{2}J_{CF}$ = 36.1 Hz, C7), 128.49 (d, $^{2}J_{CP}$ = 17.0 Hz, C3), 120.33 (q, $^{1}J_{CF}$ = 275.1 Hz, CF_3), 119.78 (d, $^{1}J_{CP}$ = 209.5 Hz, C2), 103.95 (d, $^{3}J_{CF}$ = 3.0 Hz, C6), 73.63 (d, $^{2}J_{CP}$ = 5.8 Hz, O<u>C</u>H), 23.92 (d, $^{3}J_{CP}$ = 4.3 Hz, OCH(<u>C</u>H_3)), 23.86 (d, $^{3}J_{CP}$ = 4.8 Hz, OCH(<u>C</u>H_3)). **^{31}P NMR** (162 MHz, $CDCl_3$) δ 2.46.

D. **NMR Spectra**

Dimethyl (7-methyl-5-oxo-5*H*-[1,3]thiazolo[3,2-*a*]pyrimidin-3-yl)phosphonate (3a)

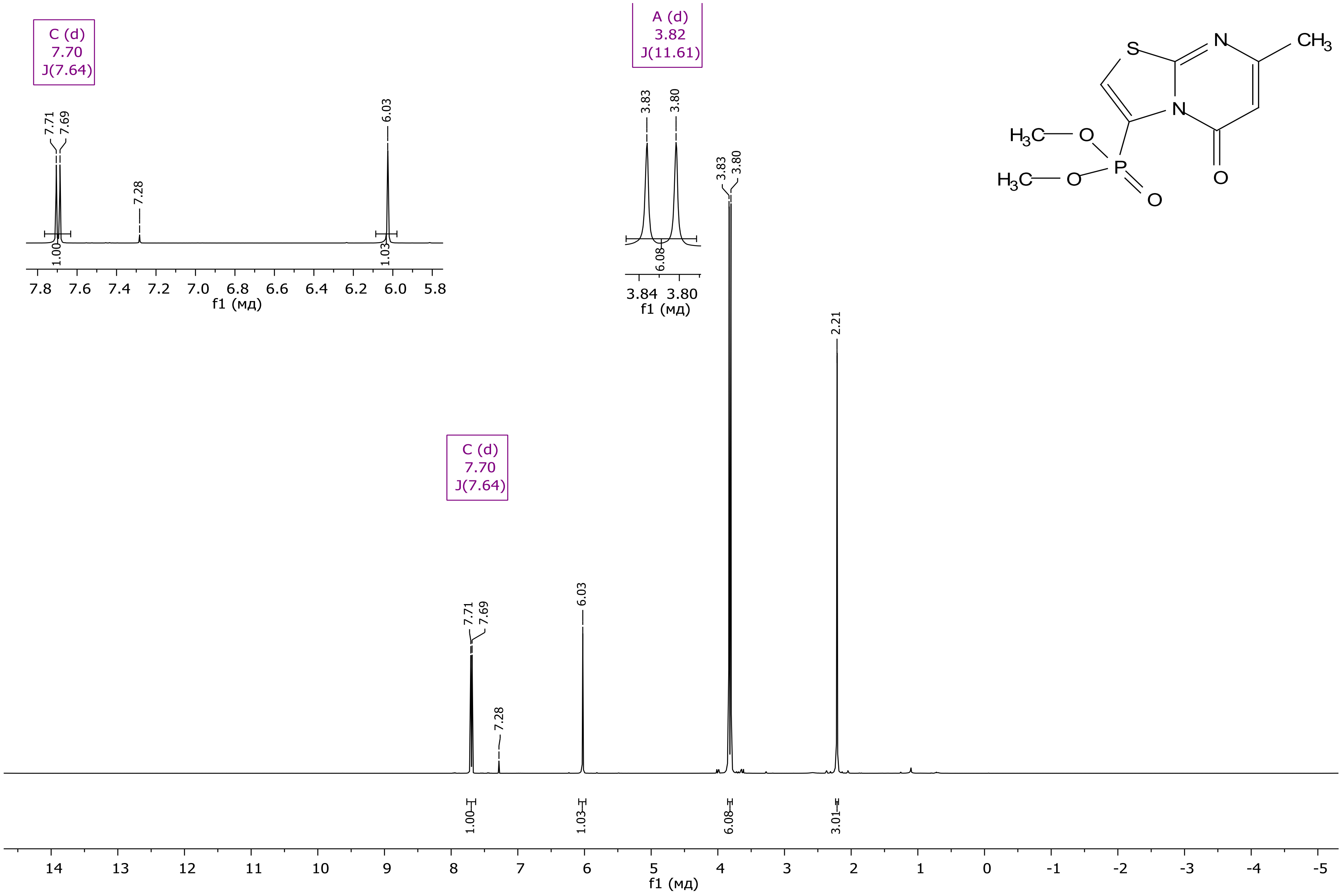

^{1}H NMR spectrum of compound **3a**

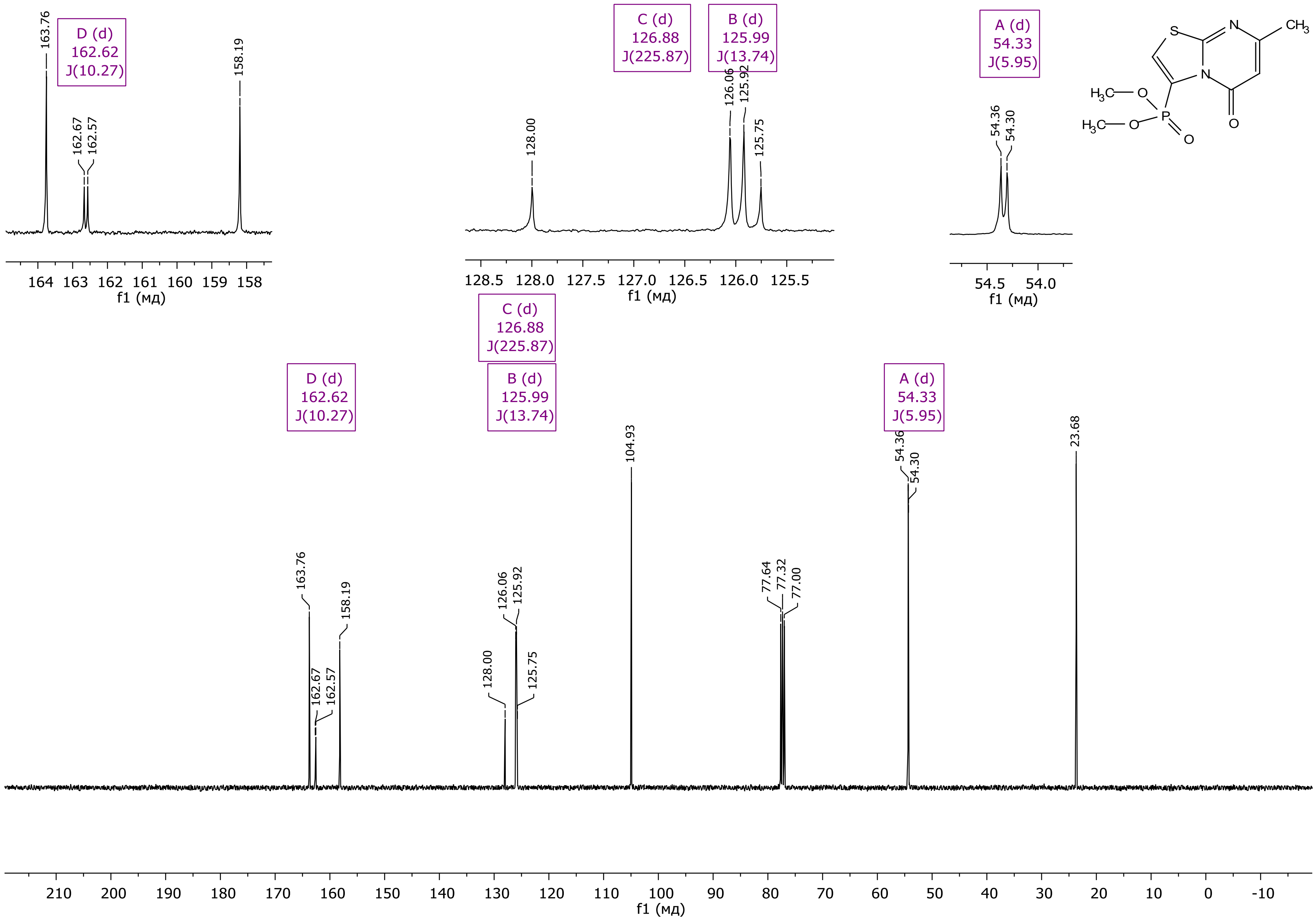

^{13}C NMR spectrum of compound **3a**

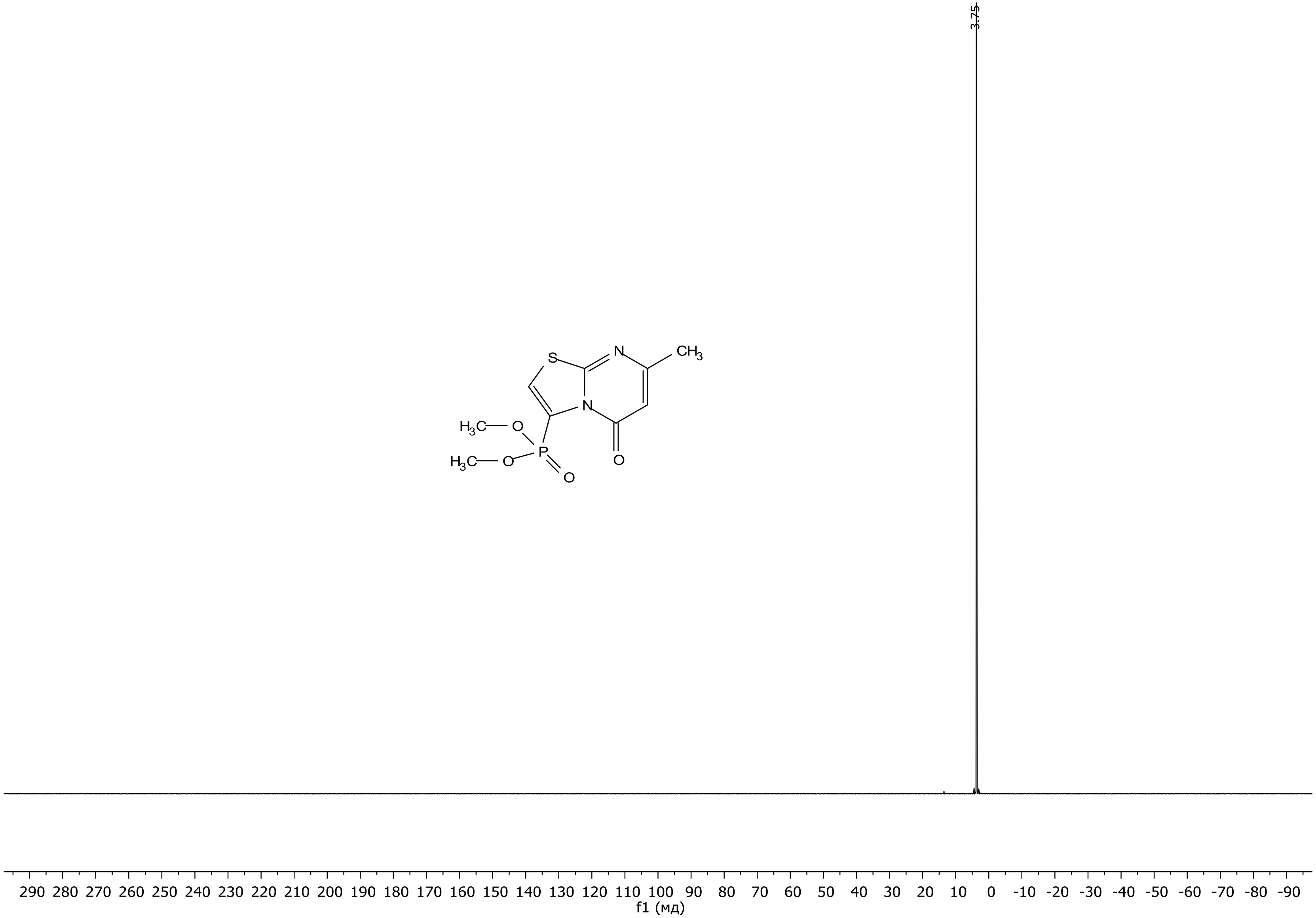

^{31}P NMR spectrum of compound **3a**

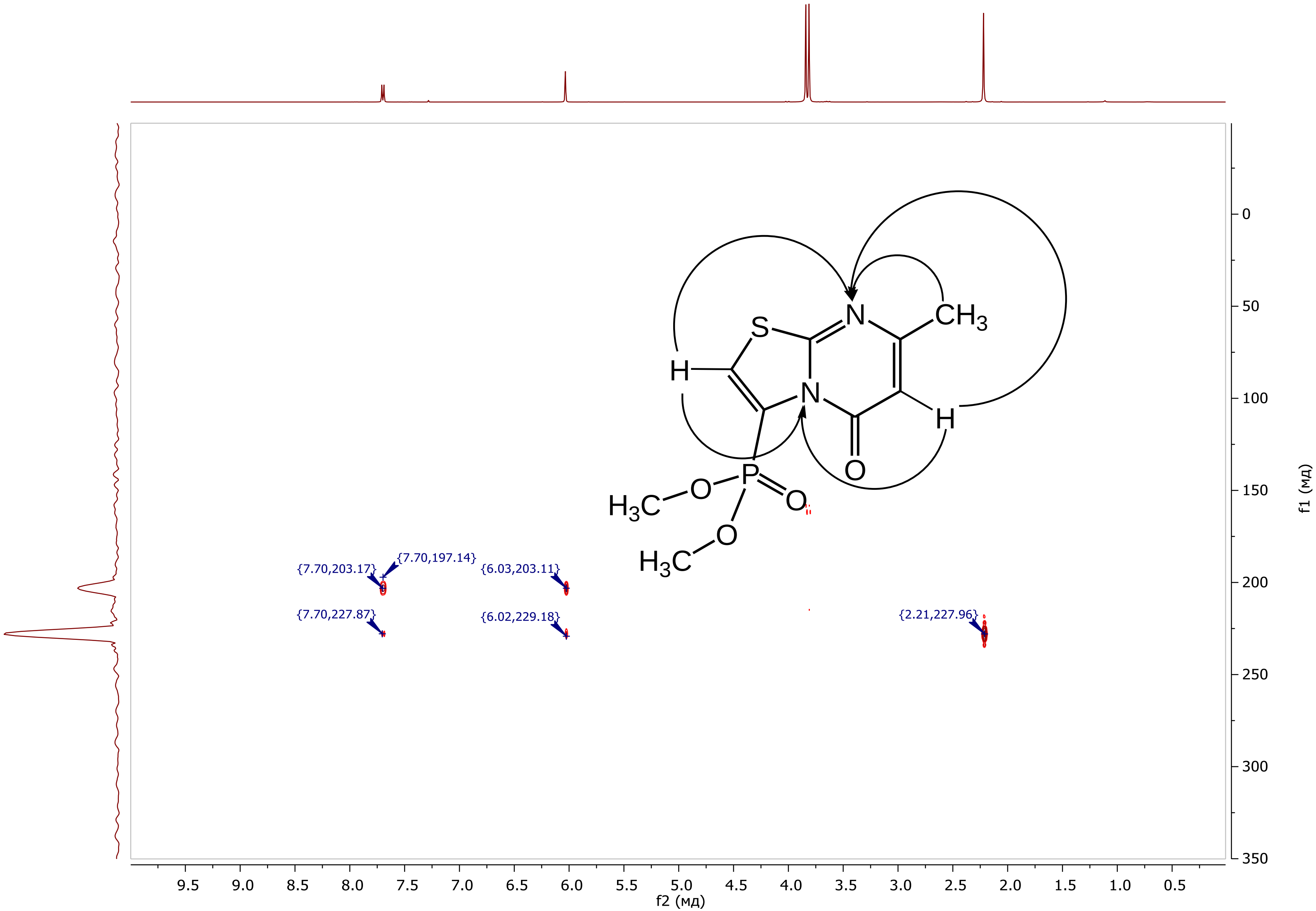

HMBC ^{1}H-^{15}N NMR spectrum of compound **3a**

Diethyl (7-methyl-5-oxo-5*H*-[1,3]thiazolo[3,2-*a*]pyrimidin-3-yl)phosphonate (3b)

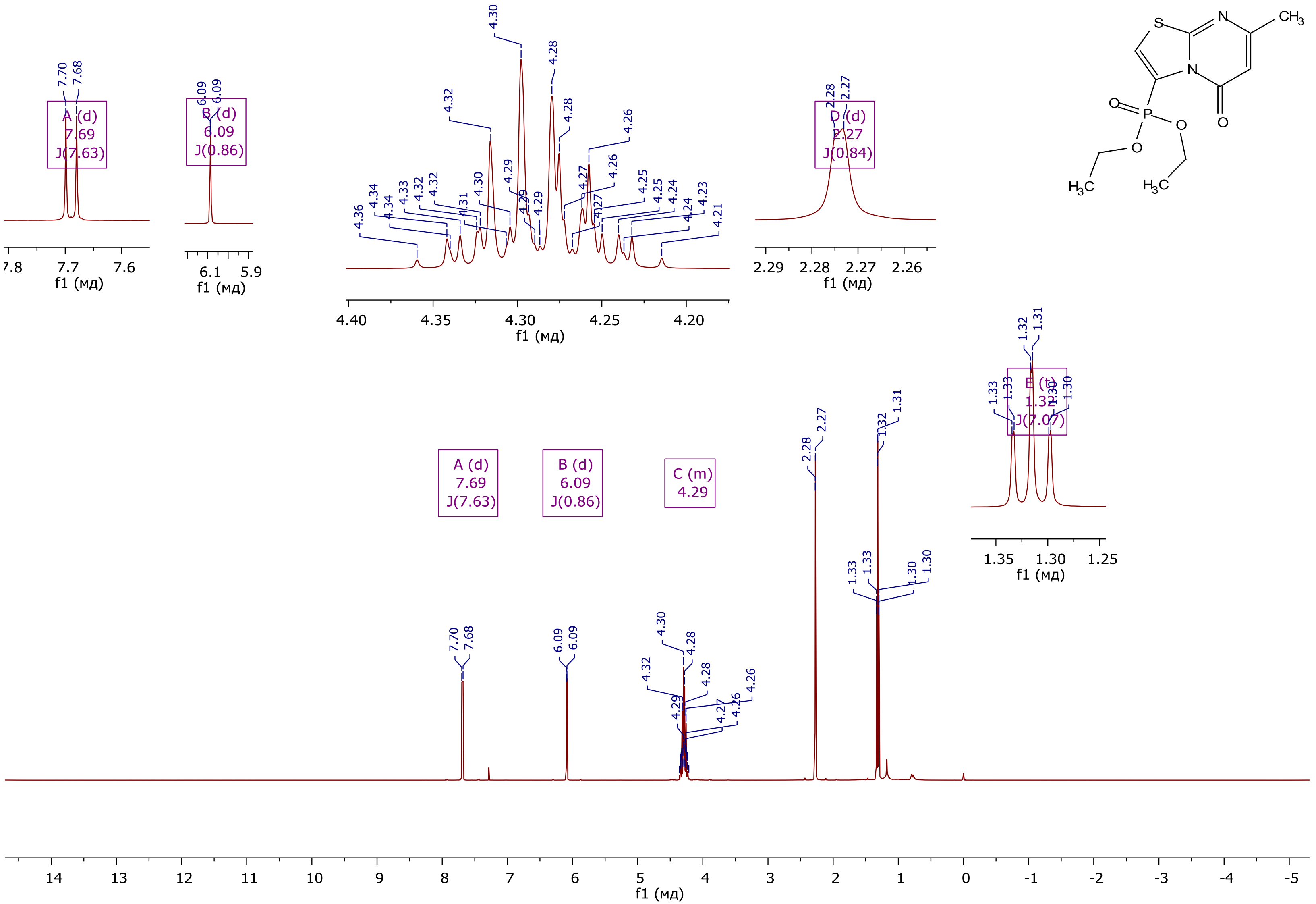

^{1}H NMR spectrum of compound **3b**

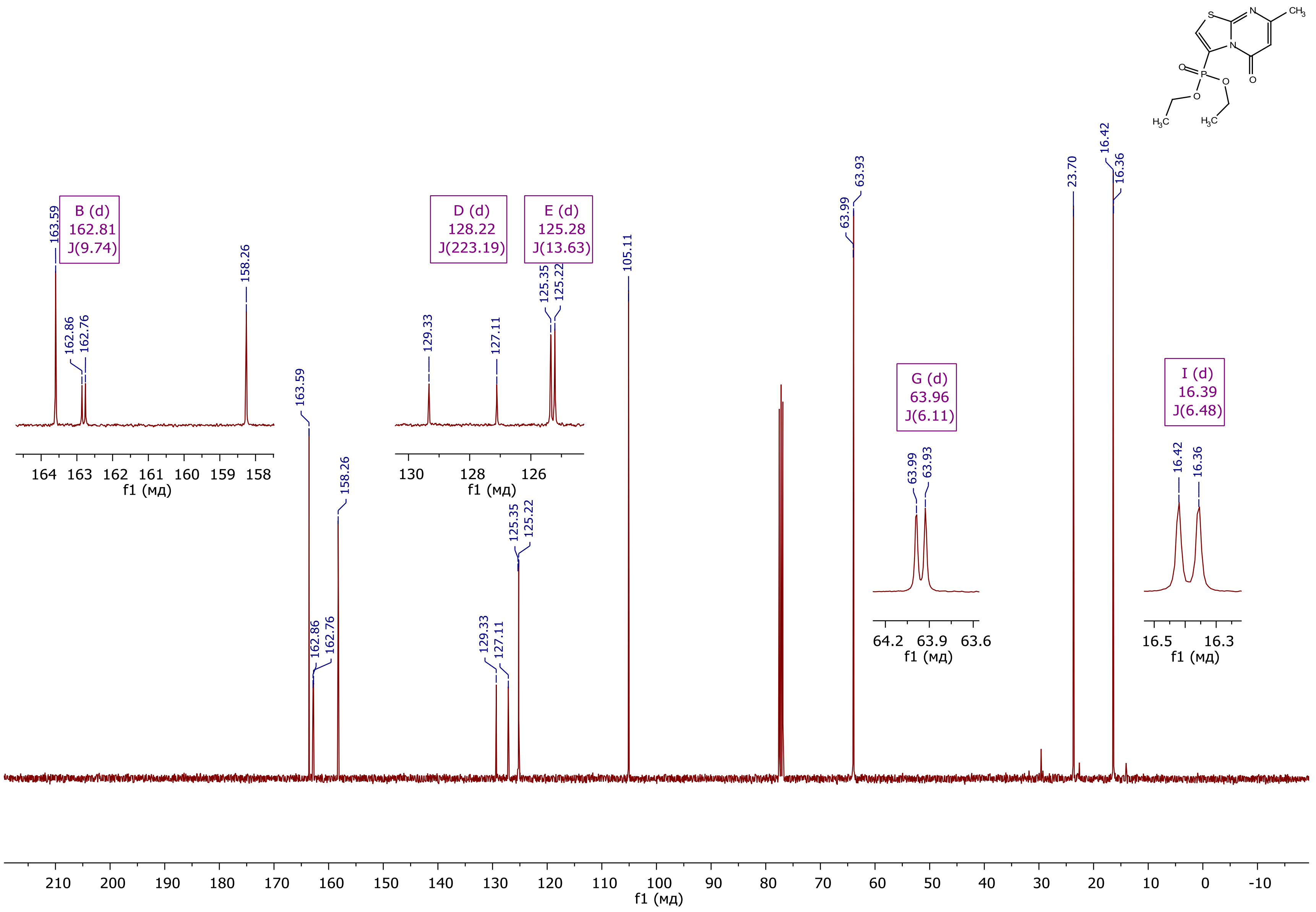

^{13}C NMR spectrum of compound **3b**

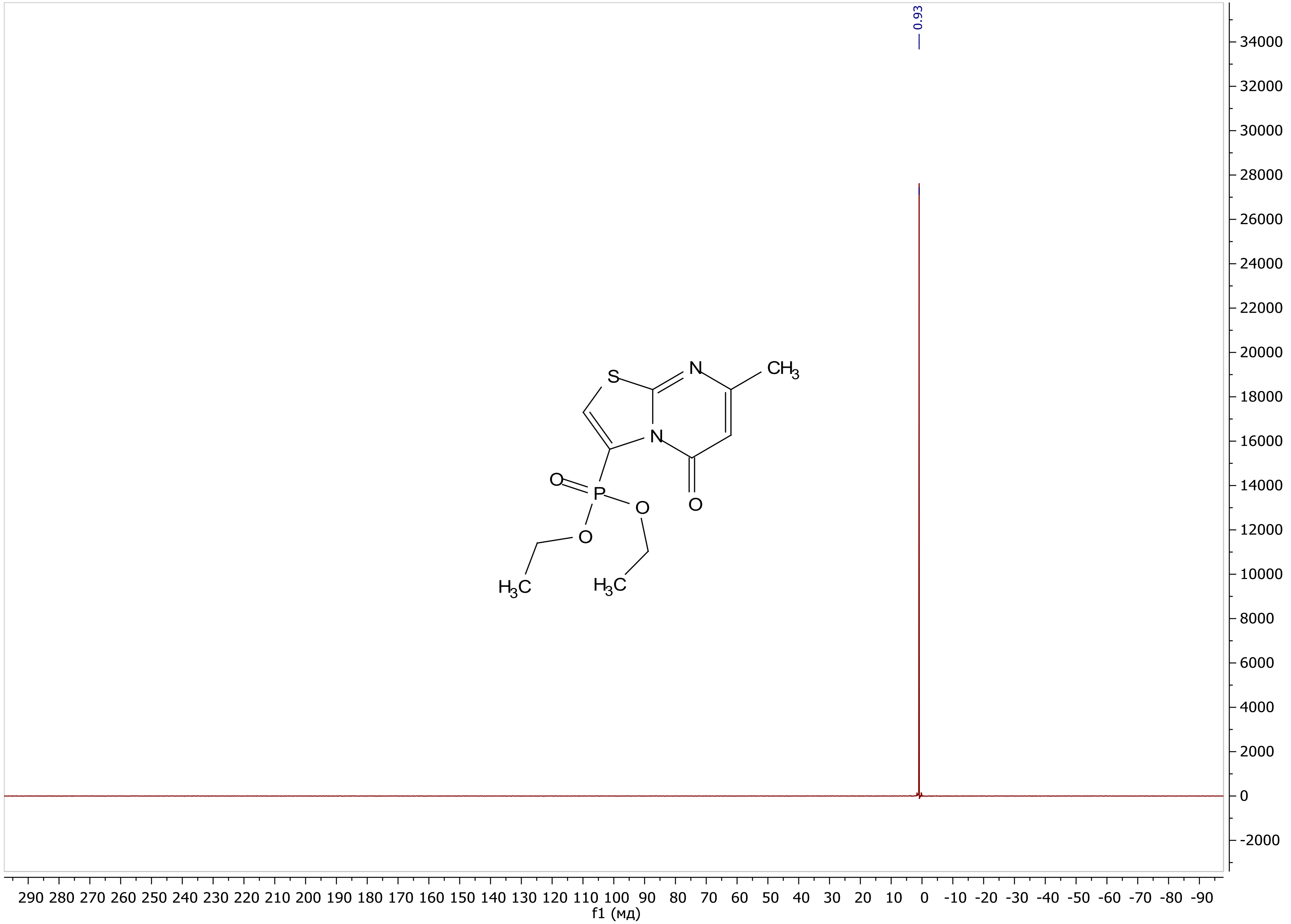

^{31}P NMR spectrum of compound **3b**

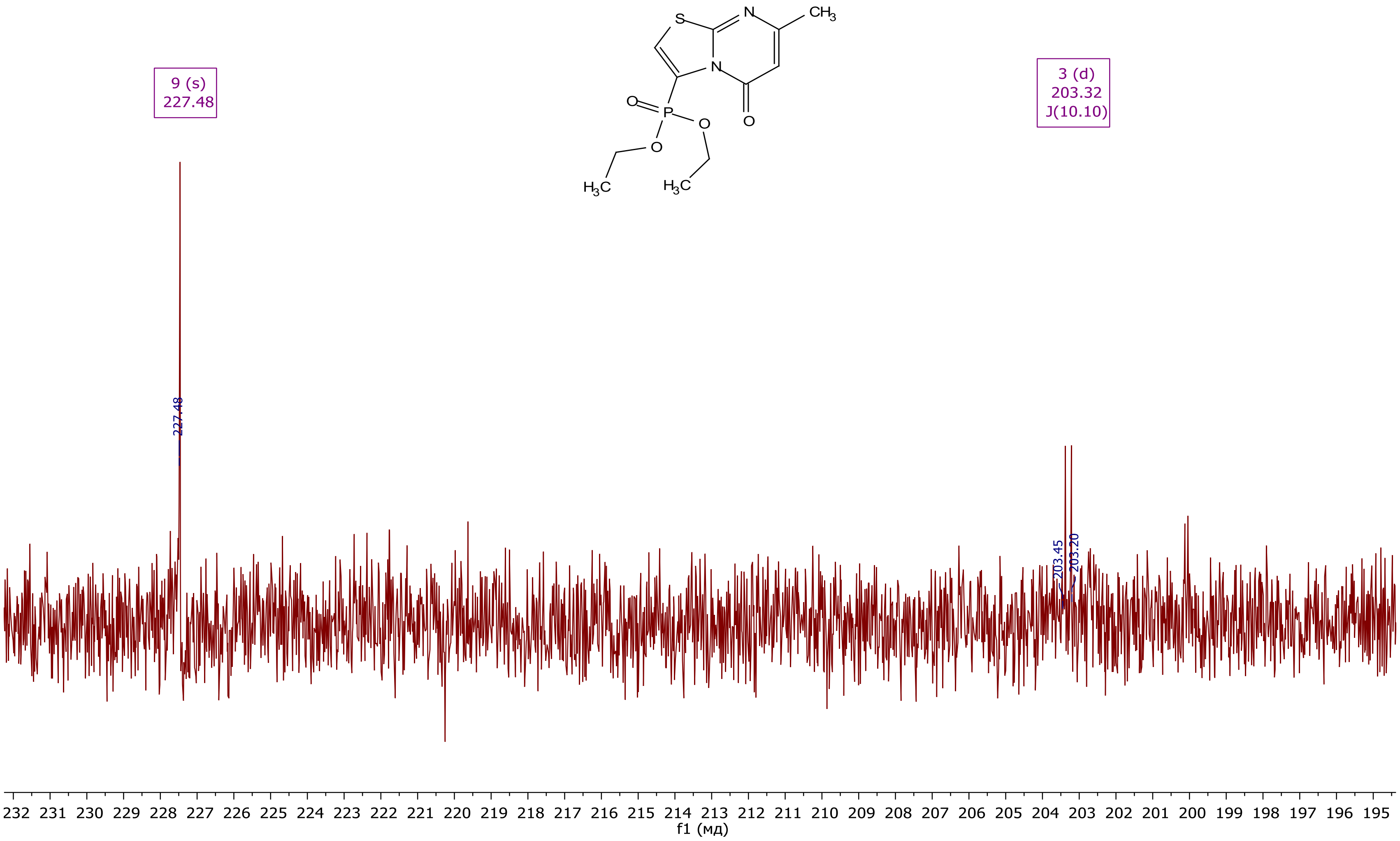

^{15}N NMR spectrum of compound **3b**

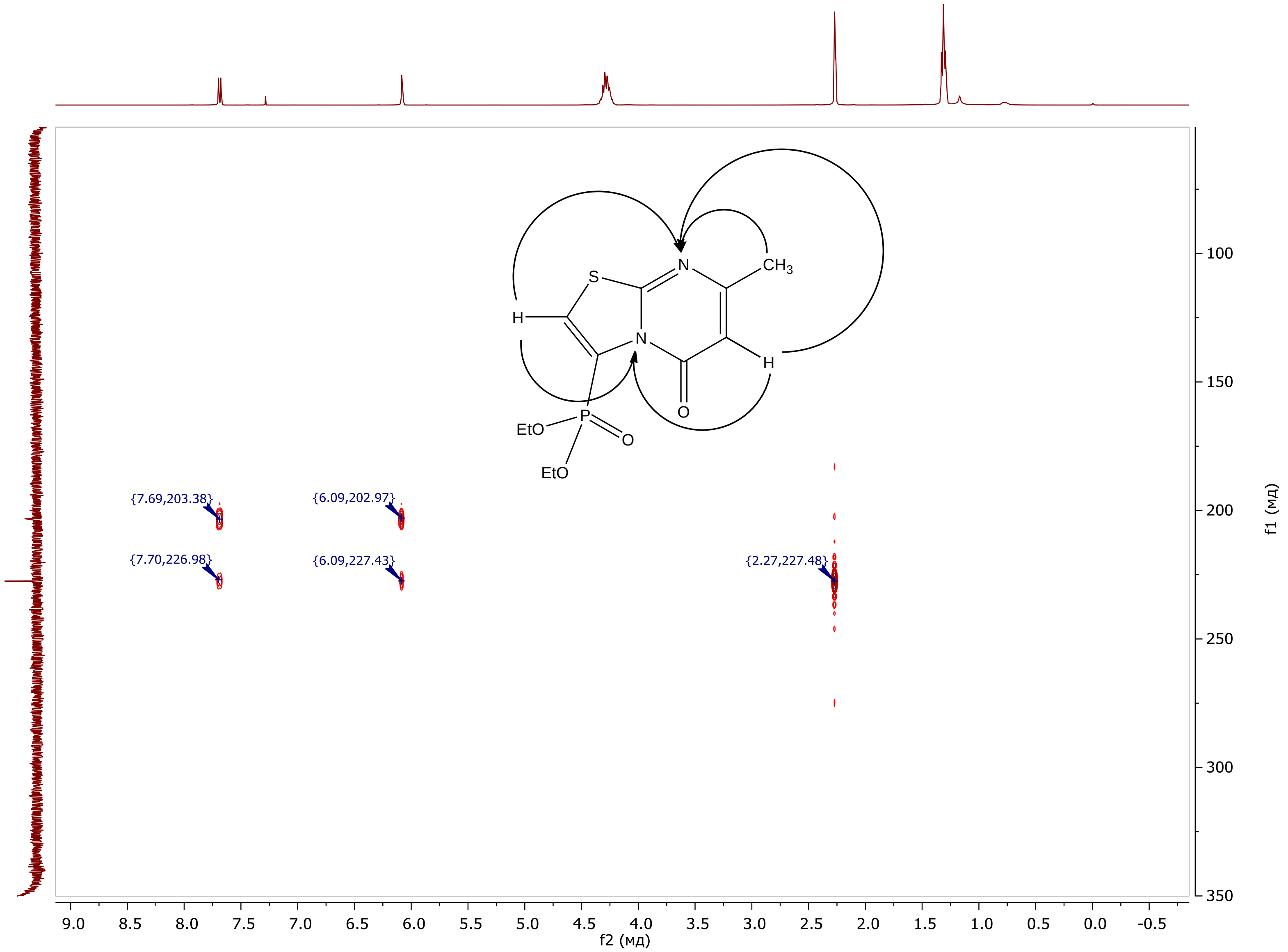

HMBC ^{1}H-^{15}N NMR spectrum of compound **3b**

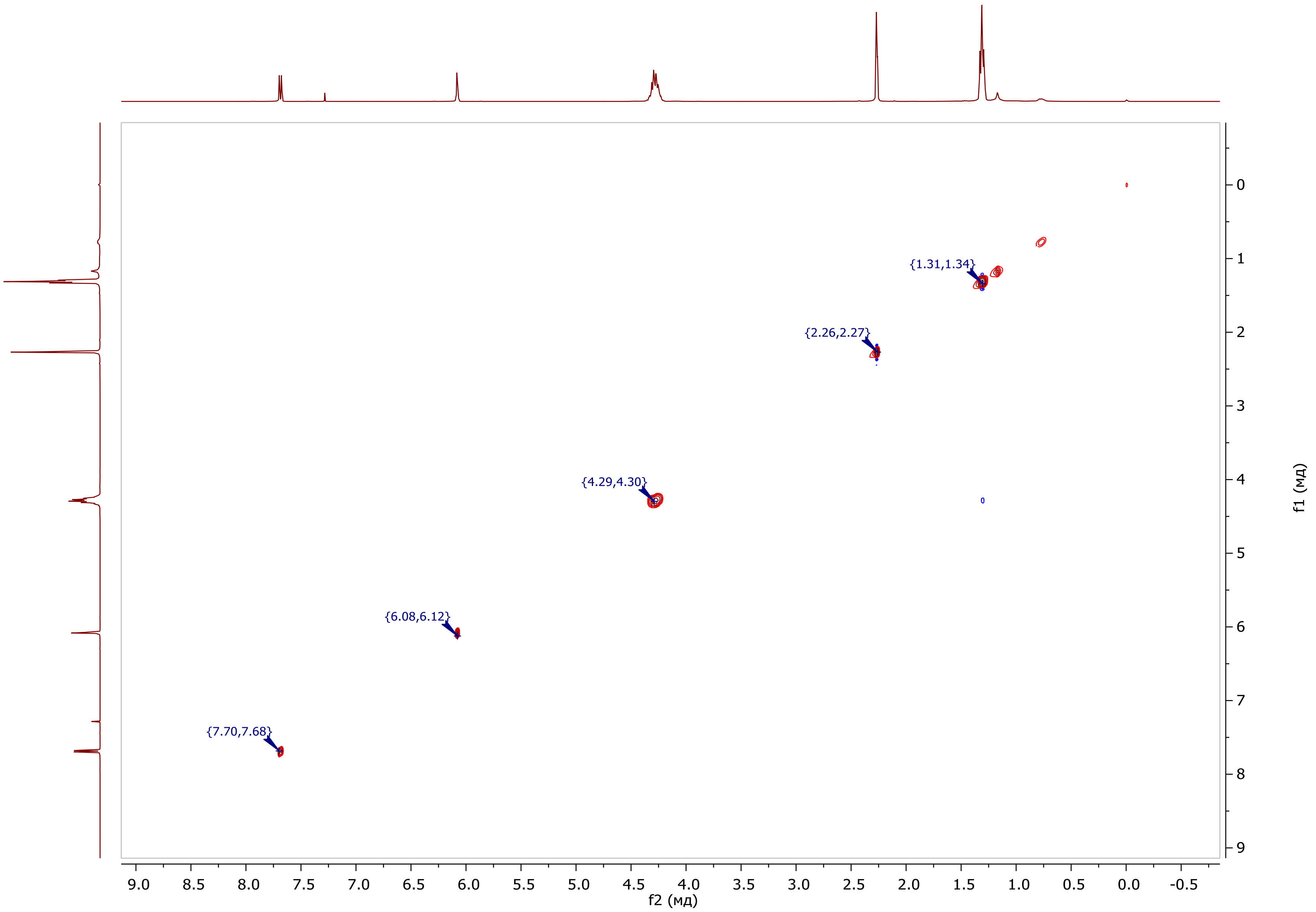

HSQC ^{1}H-^{13}C NMR spectrum of compound **3b**

Diisopropyl (7-methyl-5-oxo-5*H*-[1,3]thiazolo[3,2-*a*]pyrimidin-3-yl)phosphonate (3c)

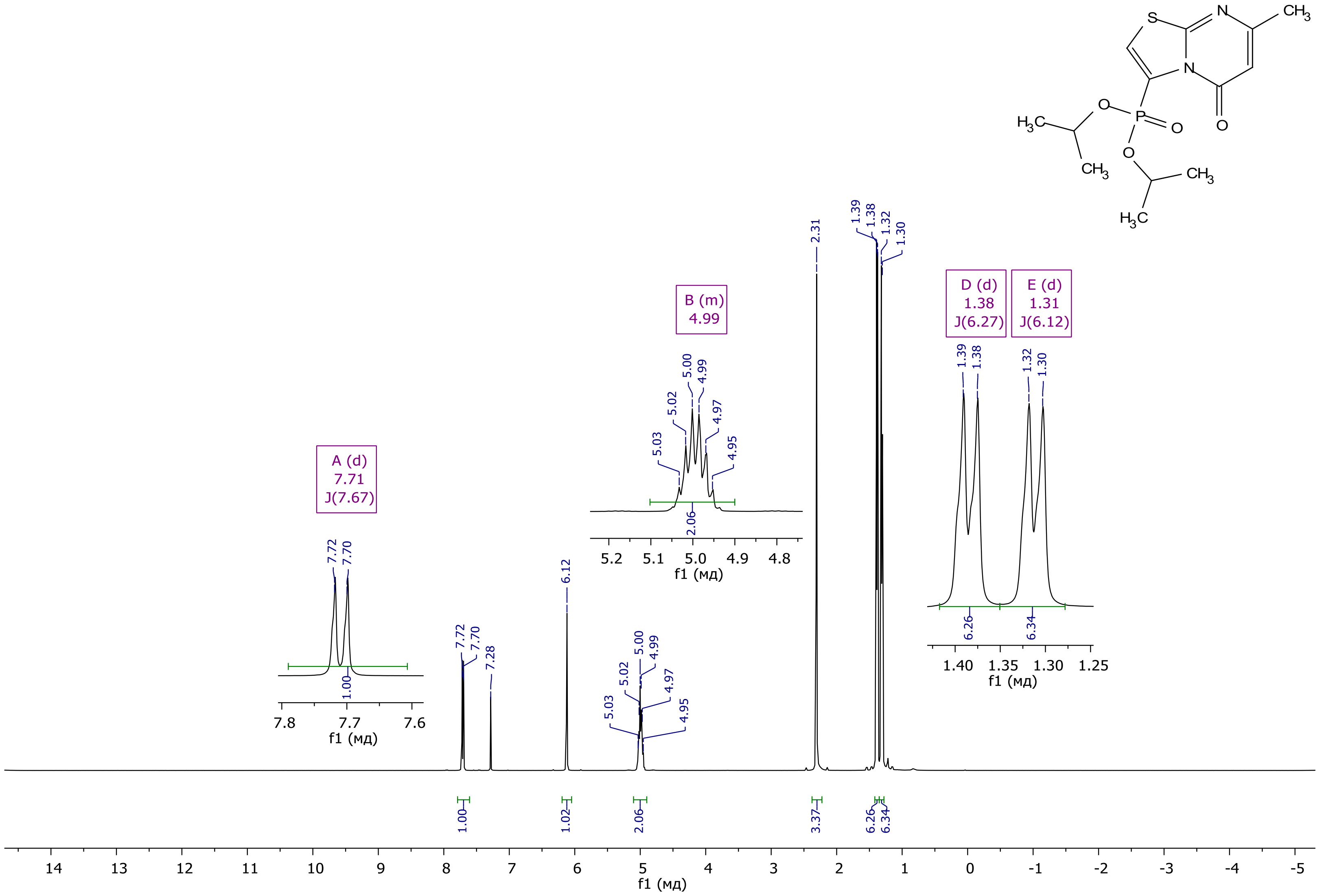

^{1}H NMR spectrum of compound **3c**

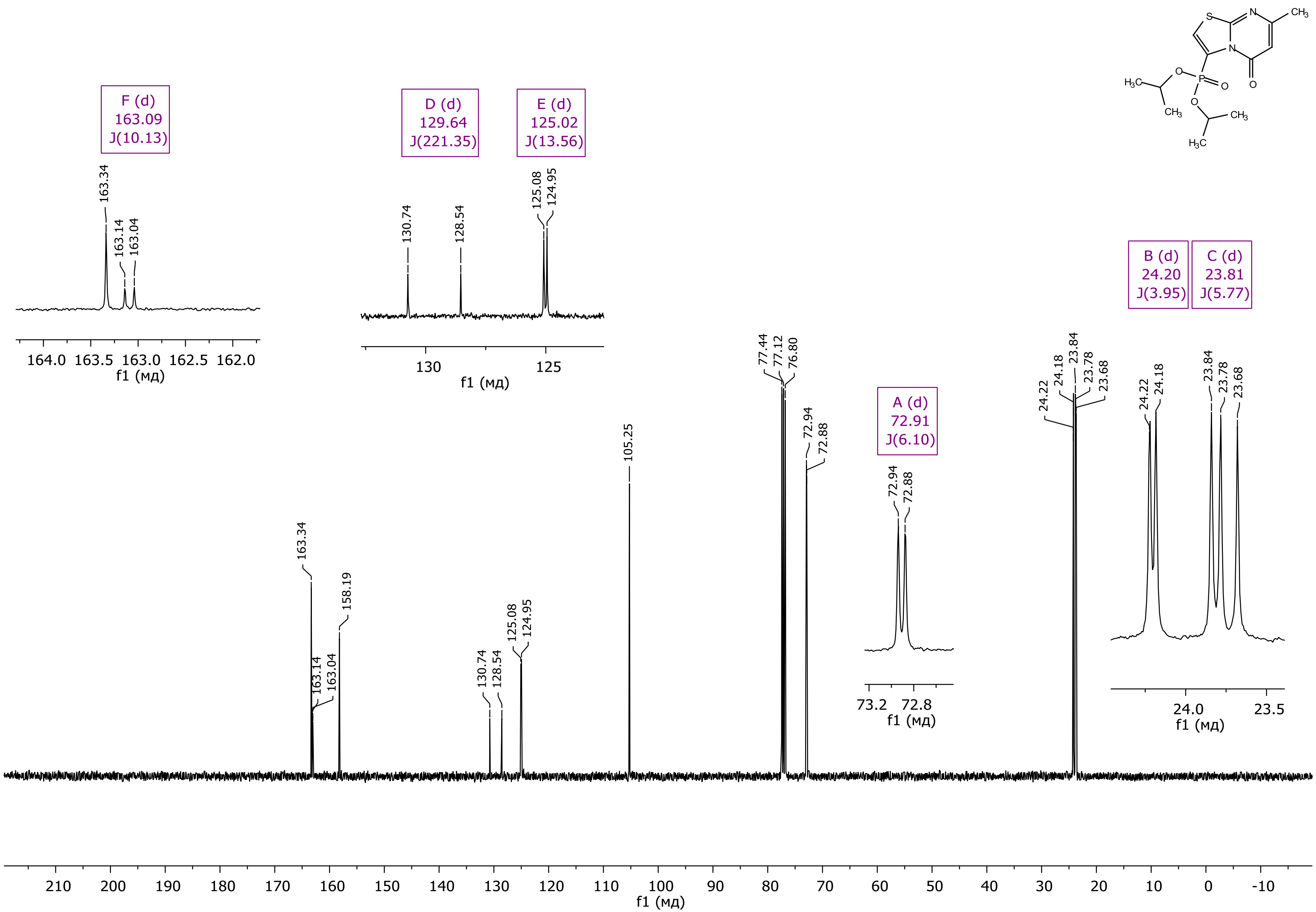

^{13}C NMR spectrum of compound **3b**

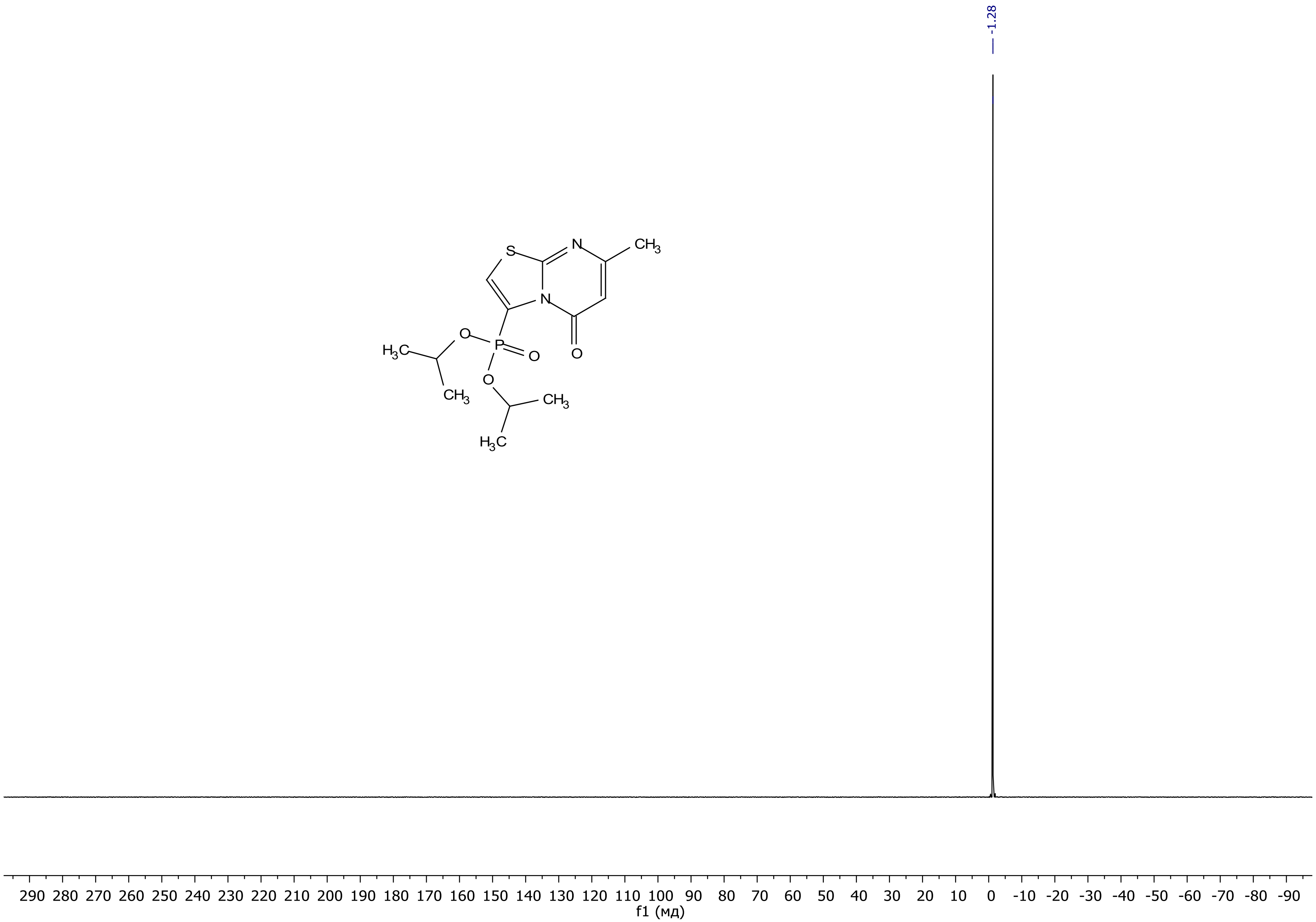

^{31}P NMR spectrum of compound **3b**

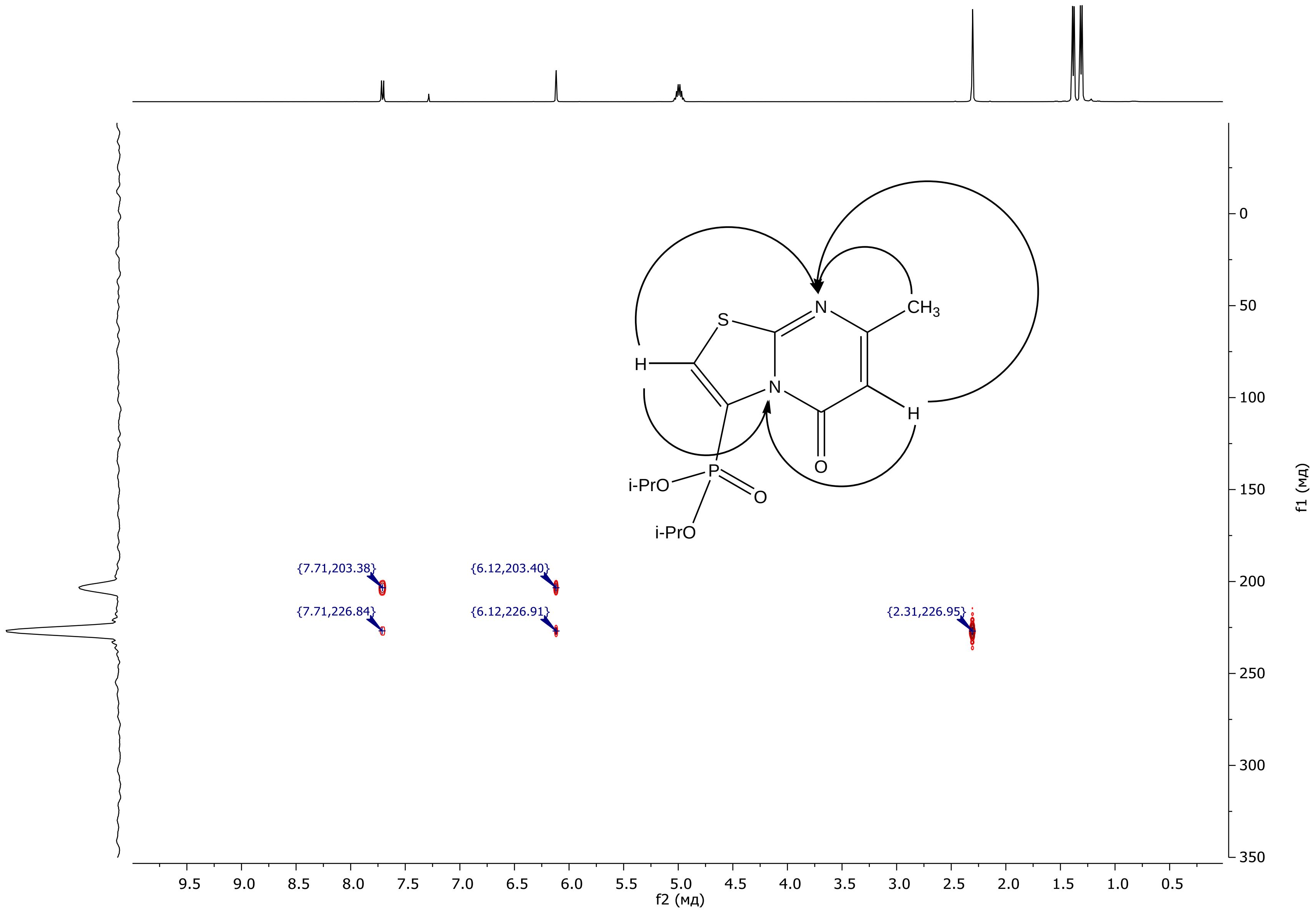

HMBC ^{1}H-^{15}N NMR spectrum of compound **3c**

Dimethyl (5-oxo-7-phenyl-5*H*-[1,3]thiazolo[3,2-*a*]pyrimidin-3-yl)phosphonate (3d)

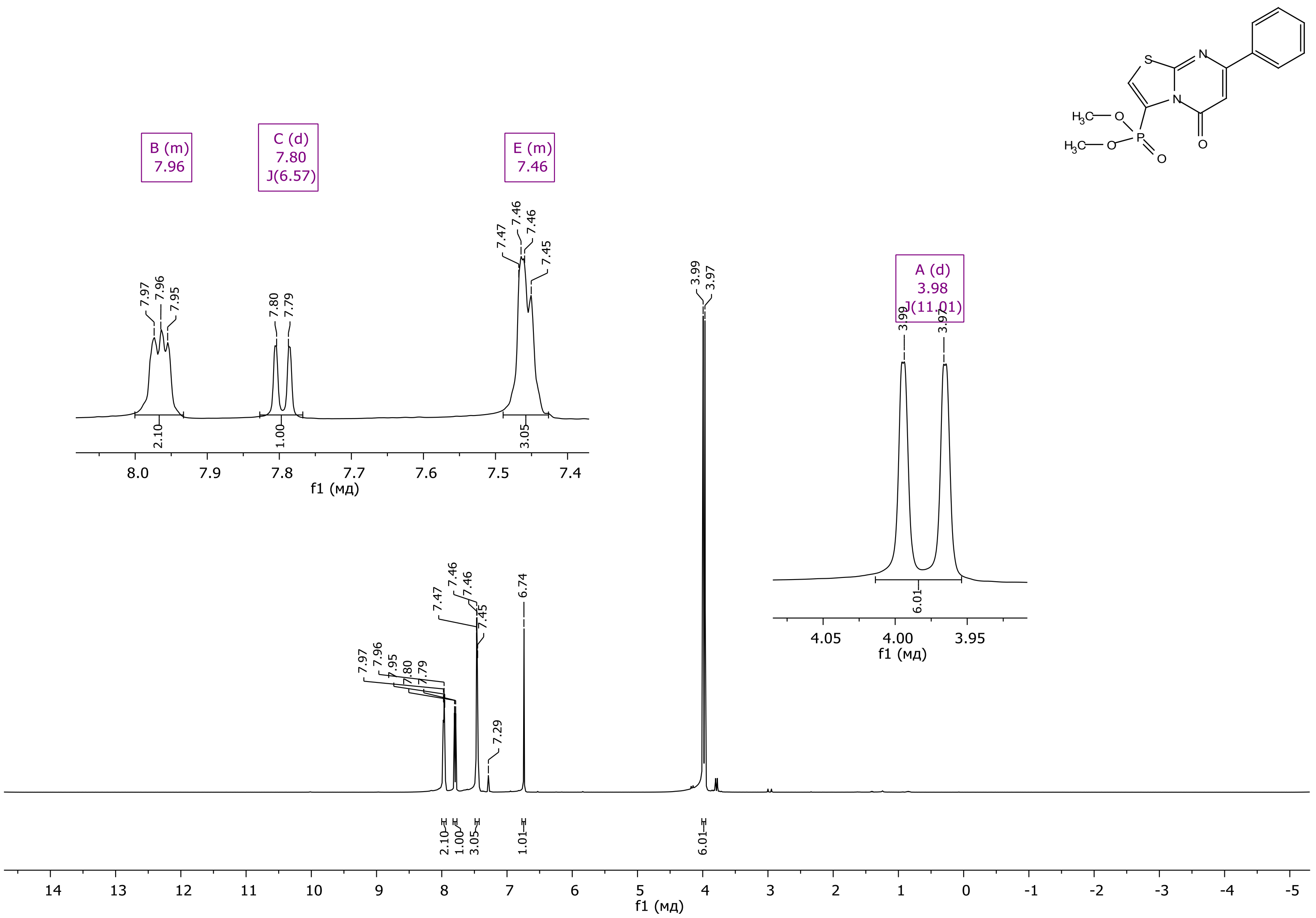

^{1}H NMR spectrum of compound **3d**

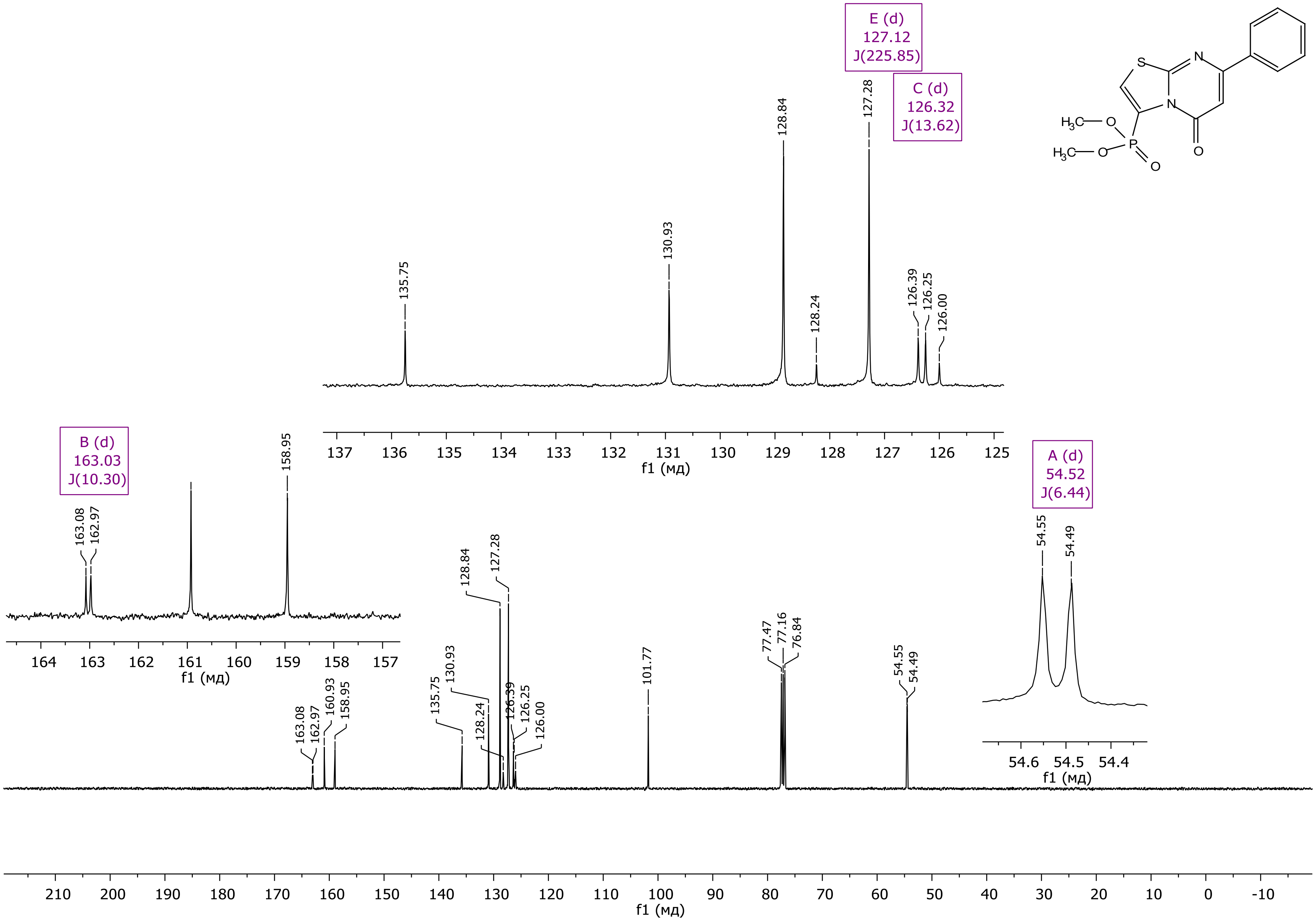

^{13}C NMR spectrum of compound **3d**

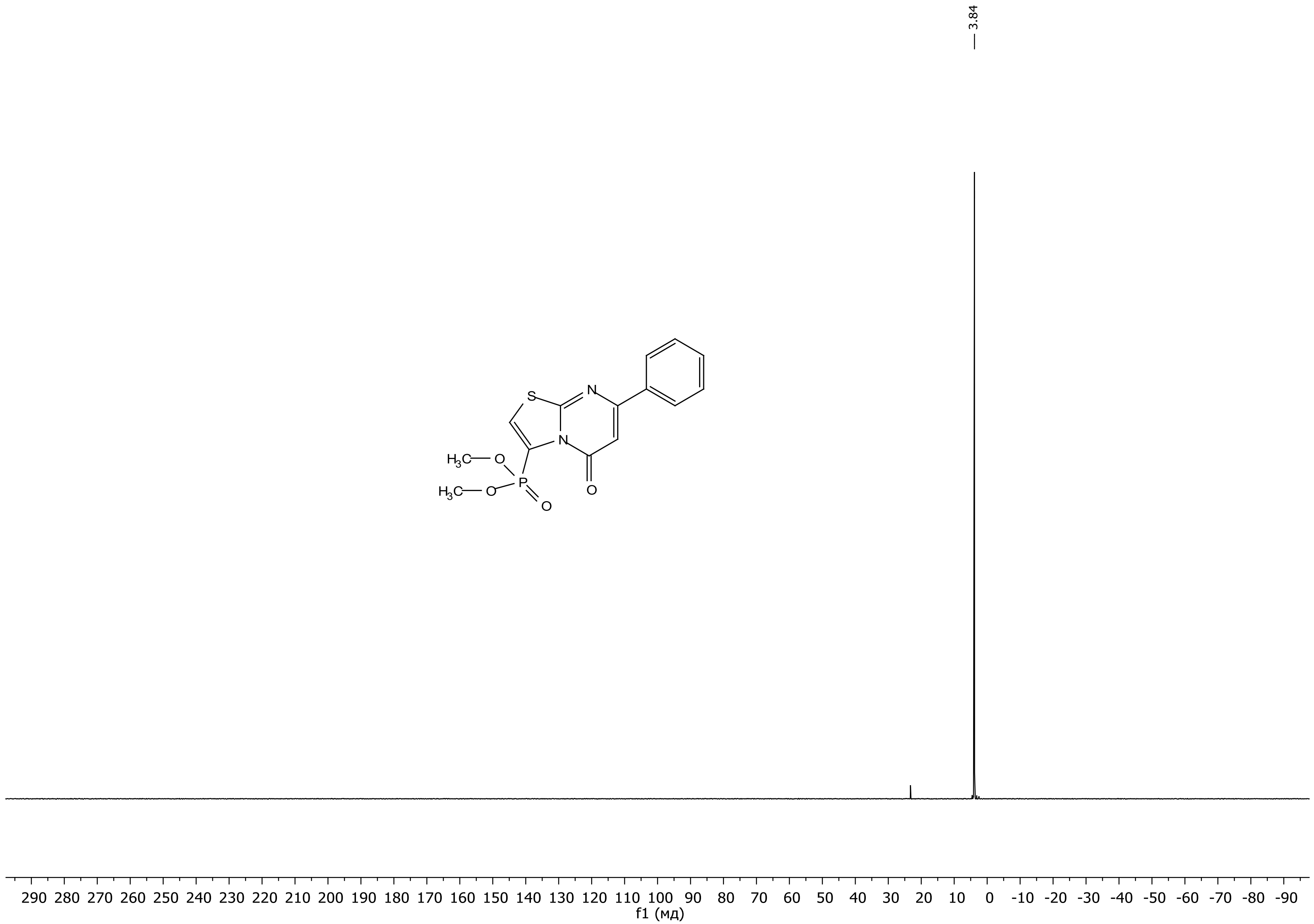

^{31}P NMR spectrum of compound **3b**

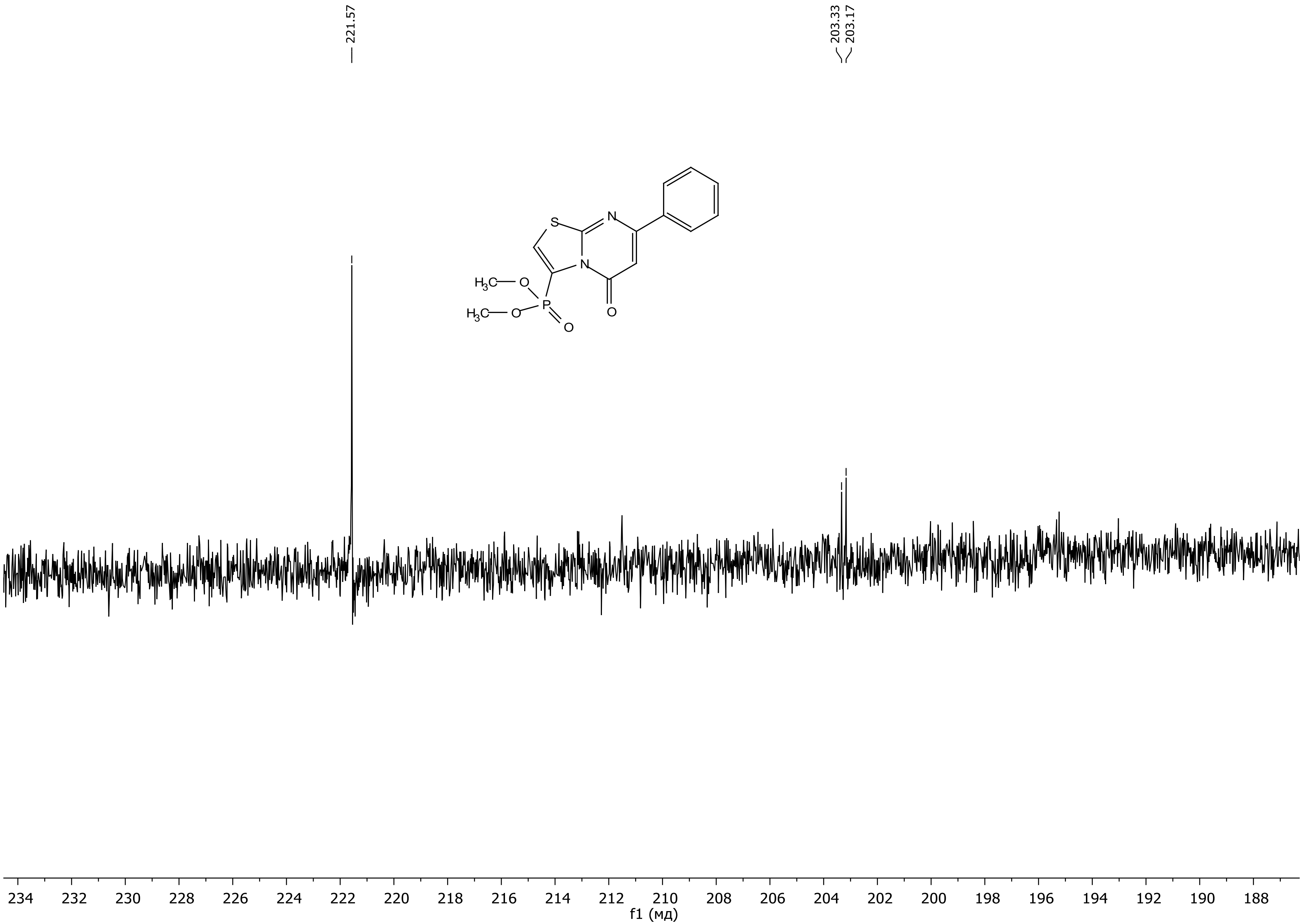

^{15}N NMR spectrum of compound **3d**

Diethyl (5-oxo-7-phenyl-5*H*-[1,3]thiazolo[3,2-*a*]pyrimidin-3-yl)phosphonate (3e)

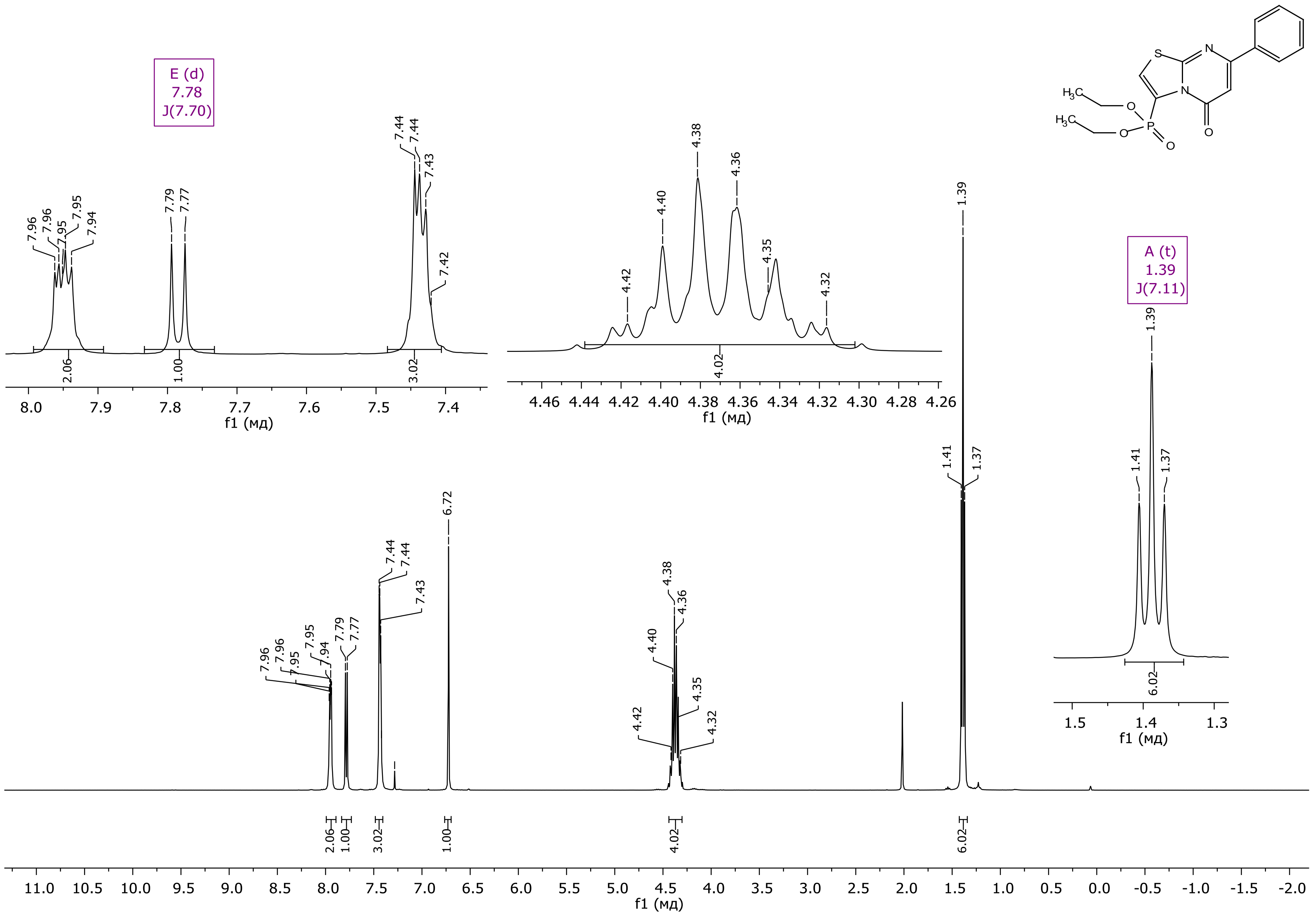

^{1}H NMR spectrum of compound **3e**

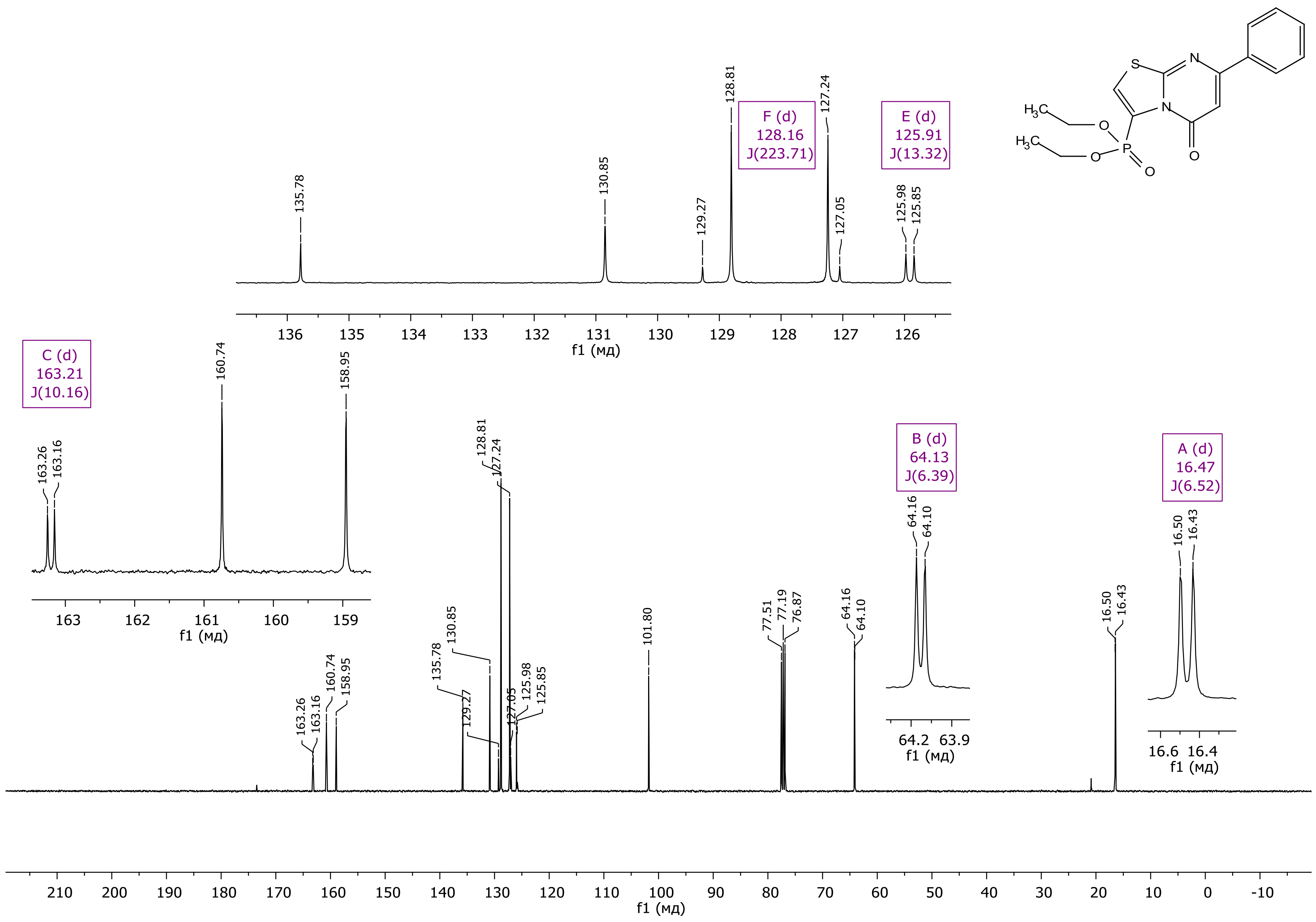

^{13}C NMR spectrum of compound **3e**

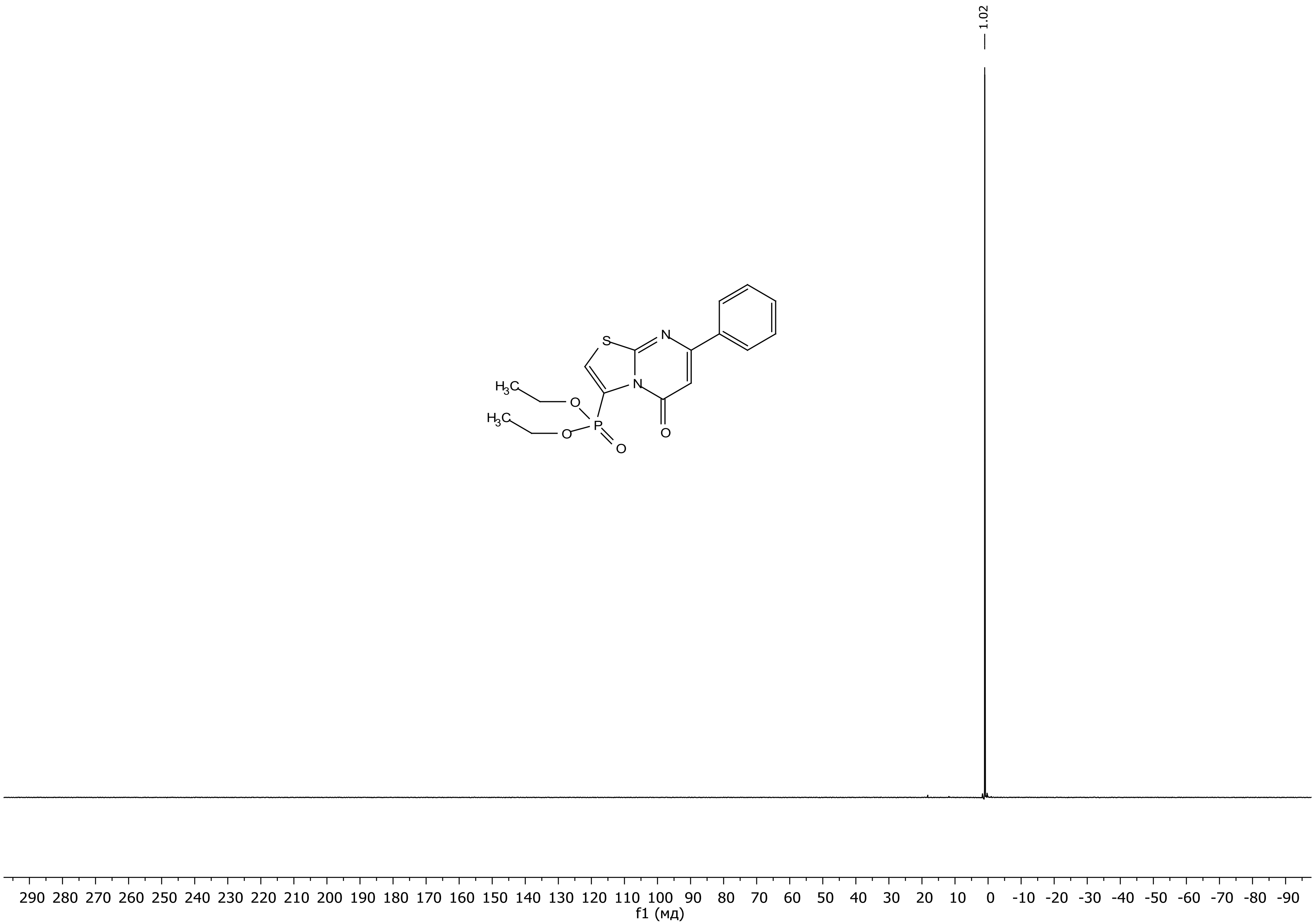

^{31}P NMR spectrum of compound **3e**

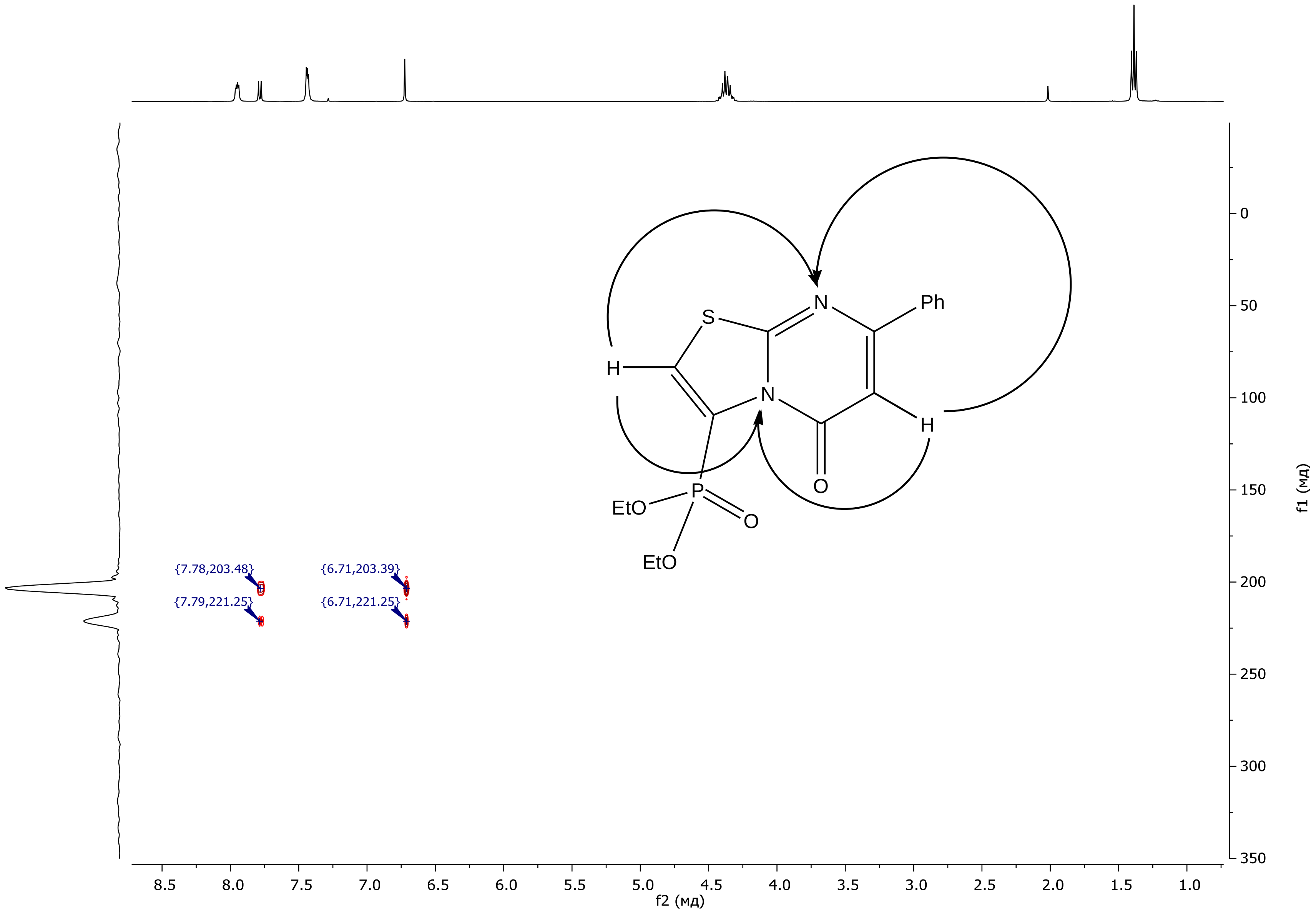

HMBC ^{1}H-^{15}N NMR spectrum of compound **3e**

Diisopropyl (5-oxo-7-phenyl-5*H*-[1,3]thiazolo[3,2-*a*]pyrimidin-3-yl)phosphonate (3f)

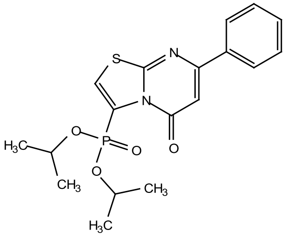

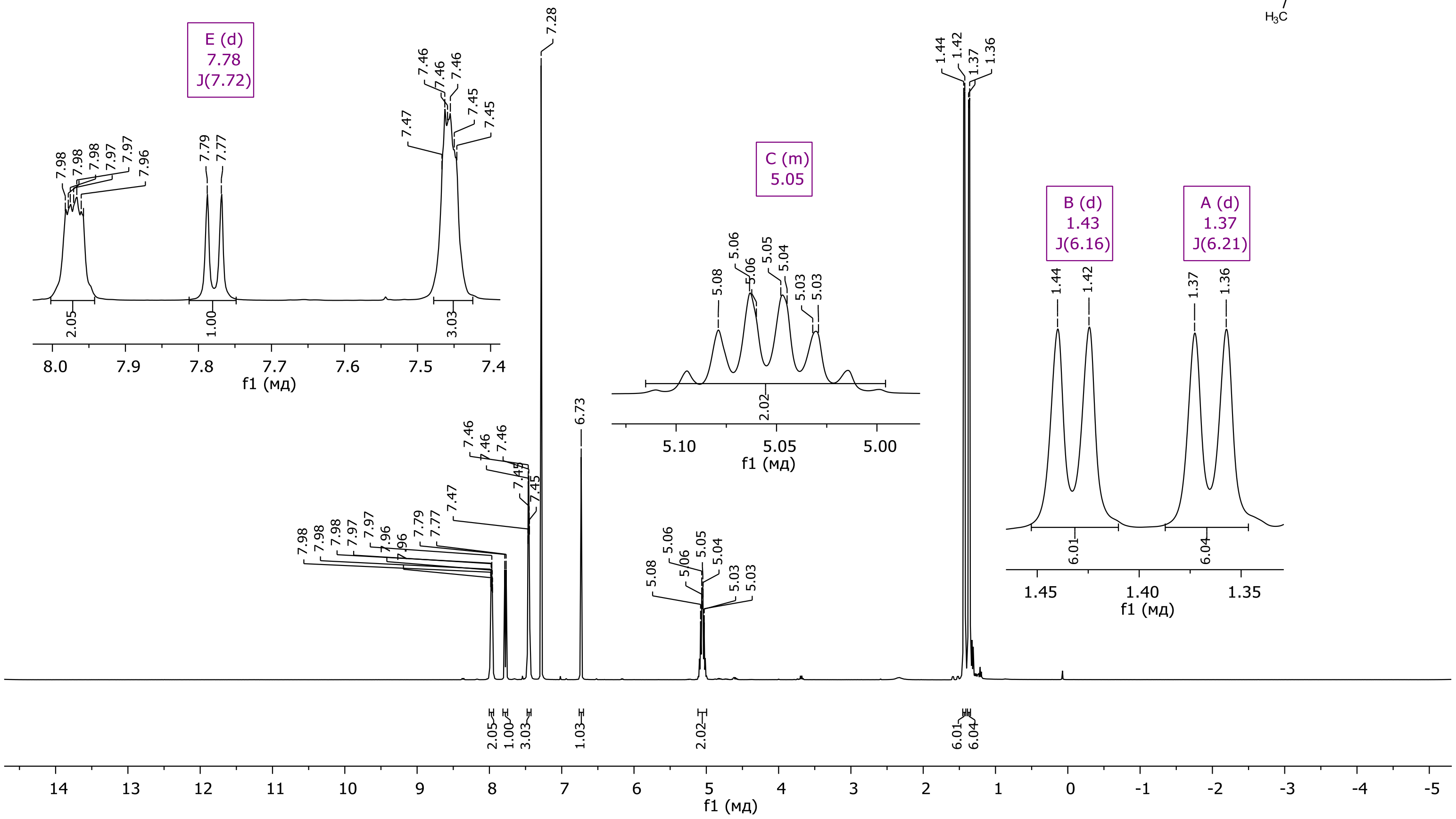

^{1}H NMR spectrum of compound **3f**

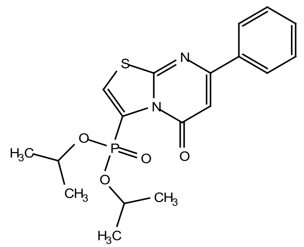

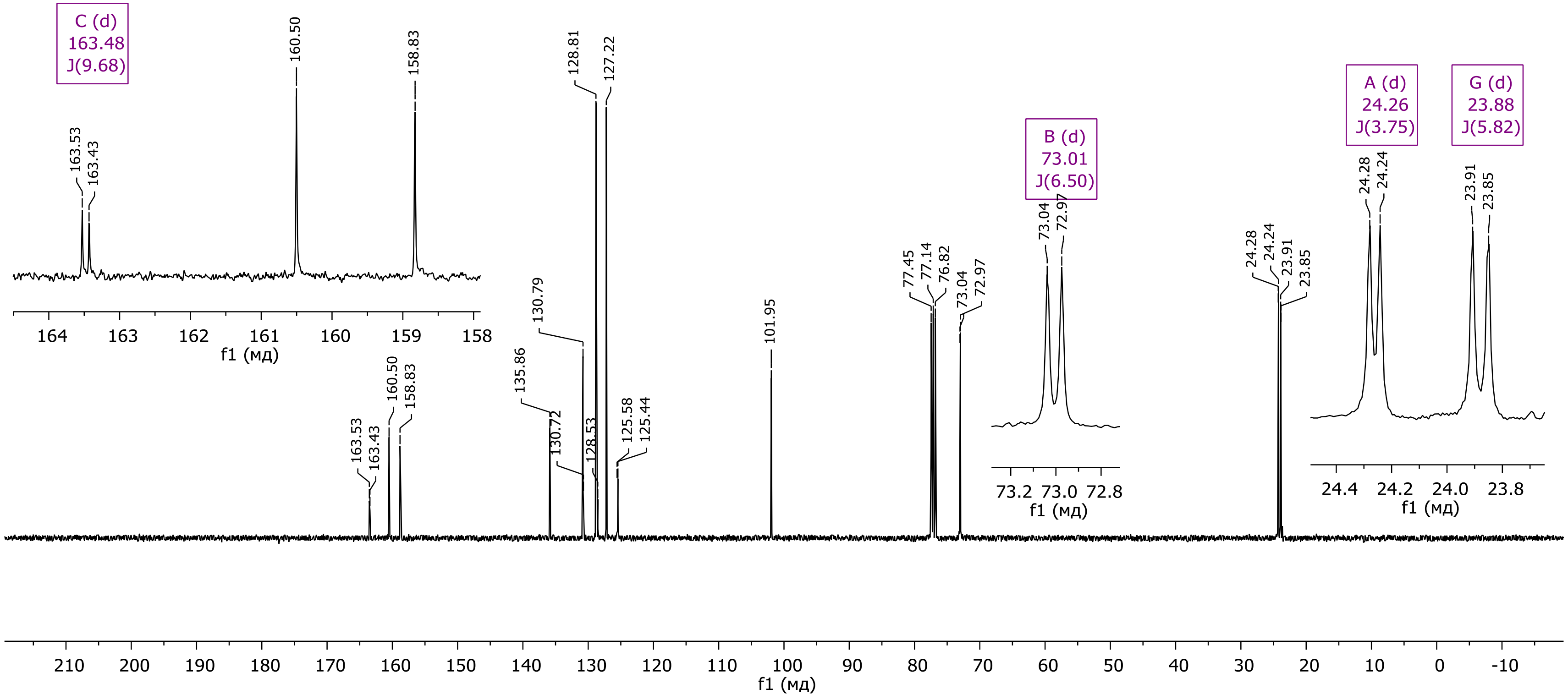

^{13}C NMR spectrum of compound **3f**

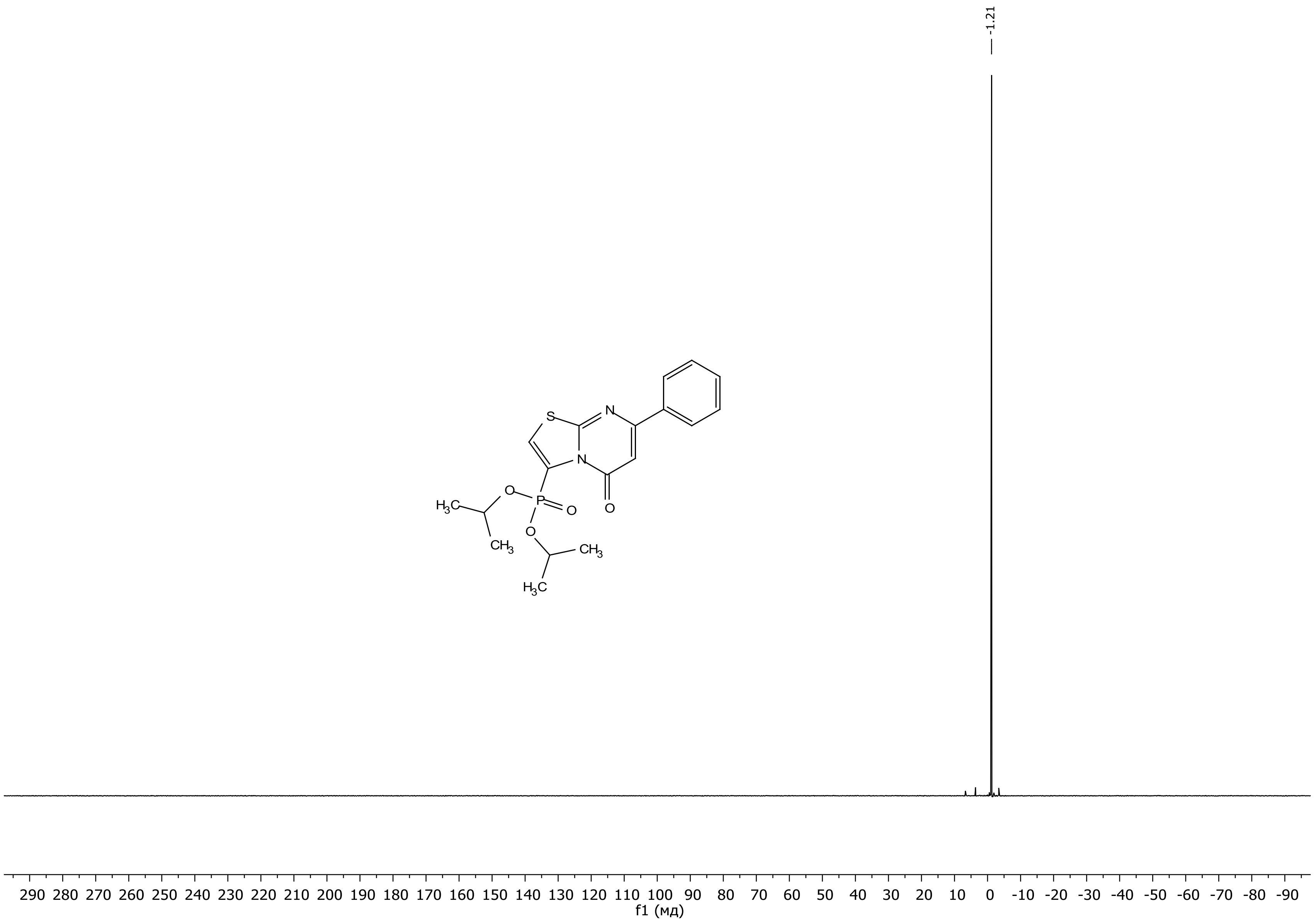

^{31}P NMR spectrum of compound **3f**

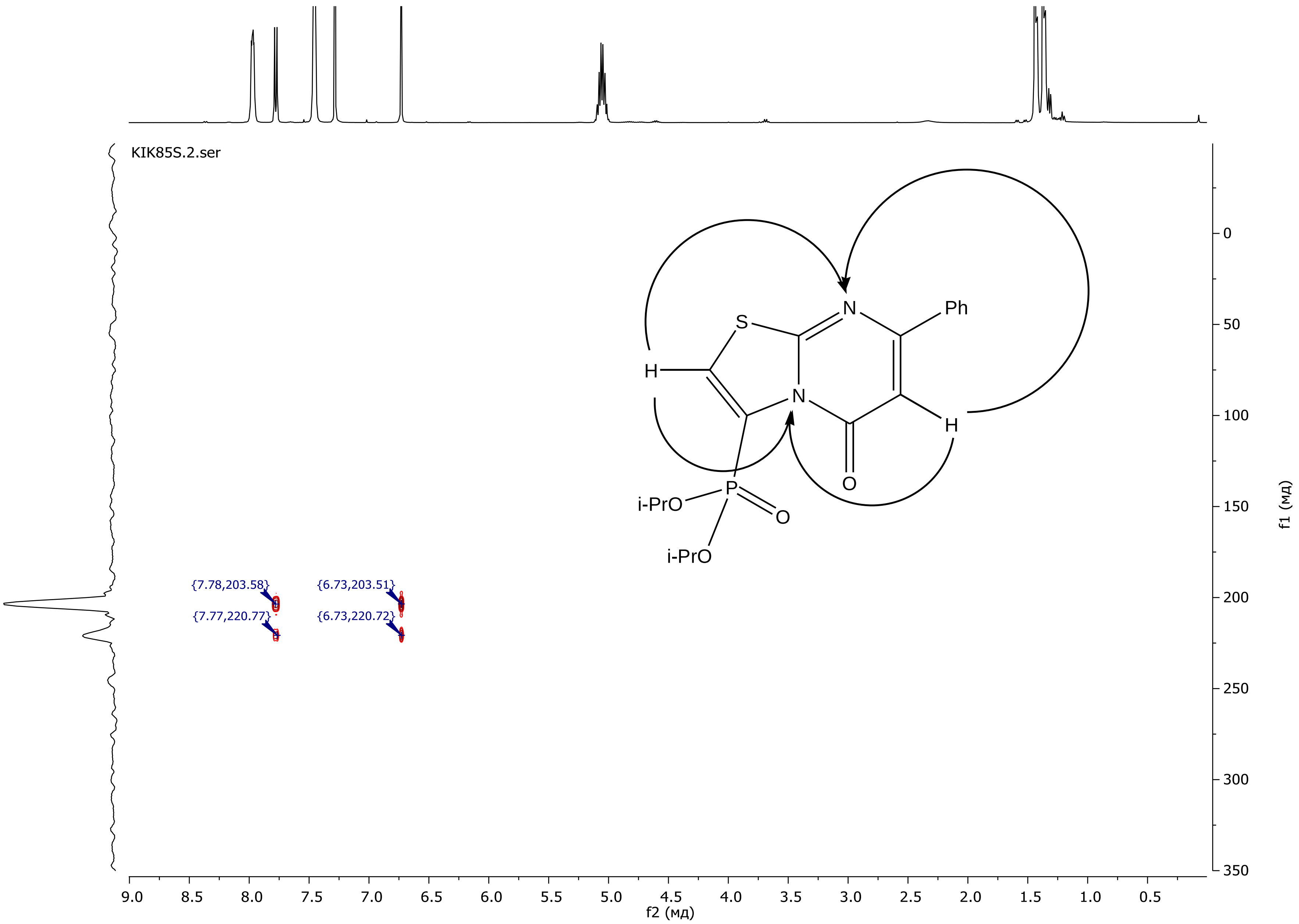

HMBC ^{1}H-^{15}N NMR spectrum of compound **3f**

Dimethyl (6-methyl-7-oxo-7*H*-[1,3]thiazolo[3,2-*a*]pyrimidin-3-yl)phosphonate (4a)

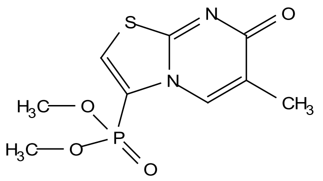

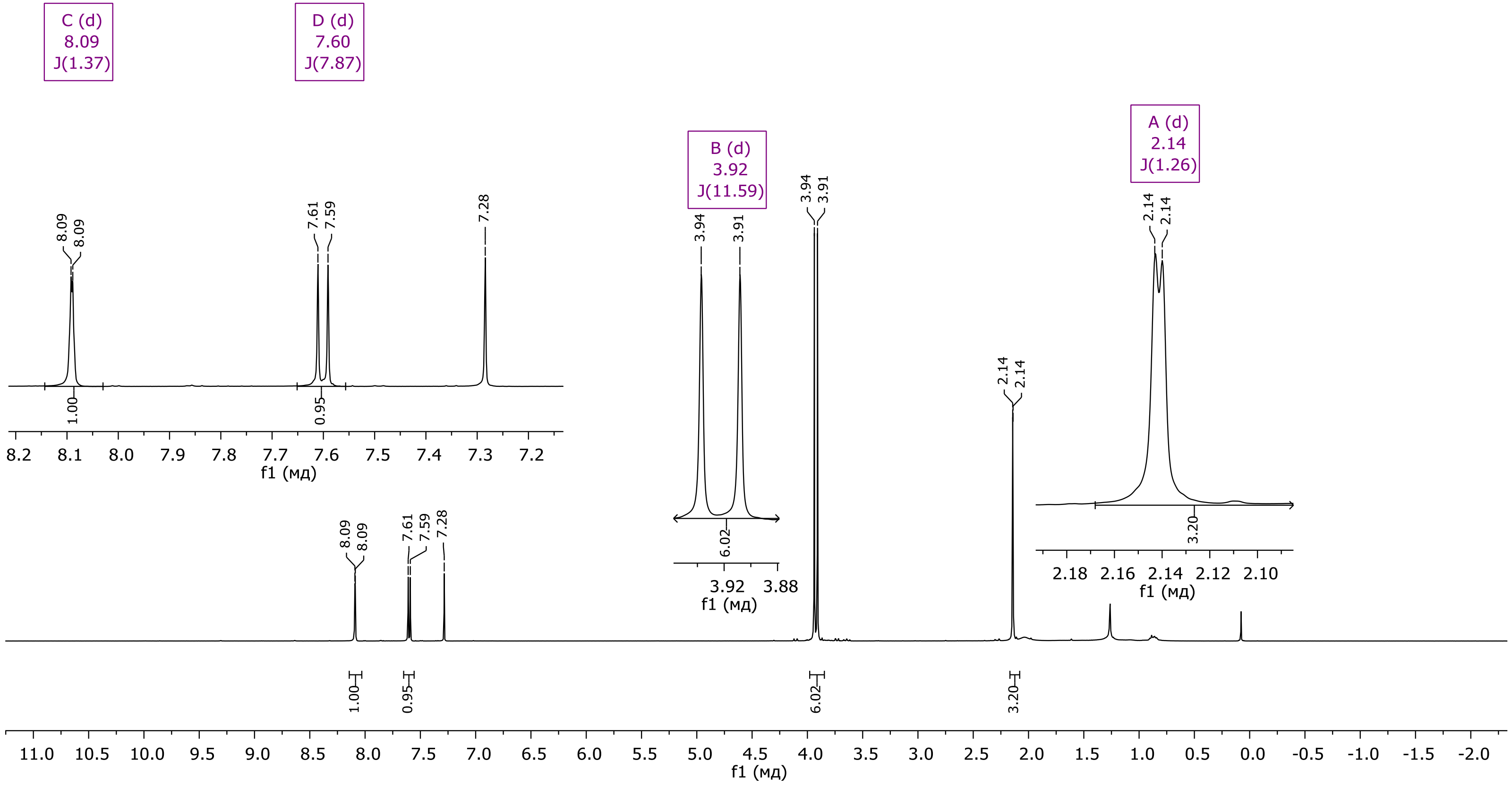

^{1}H NMR spectrum of compound **4a**

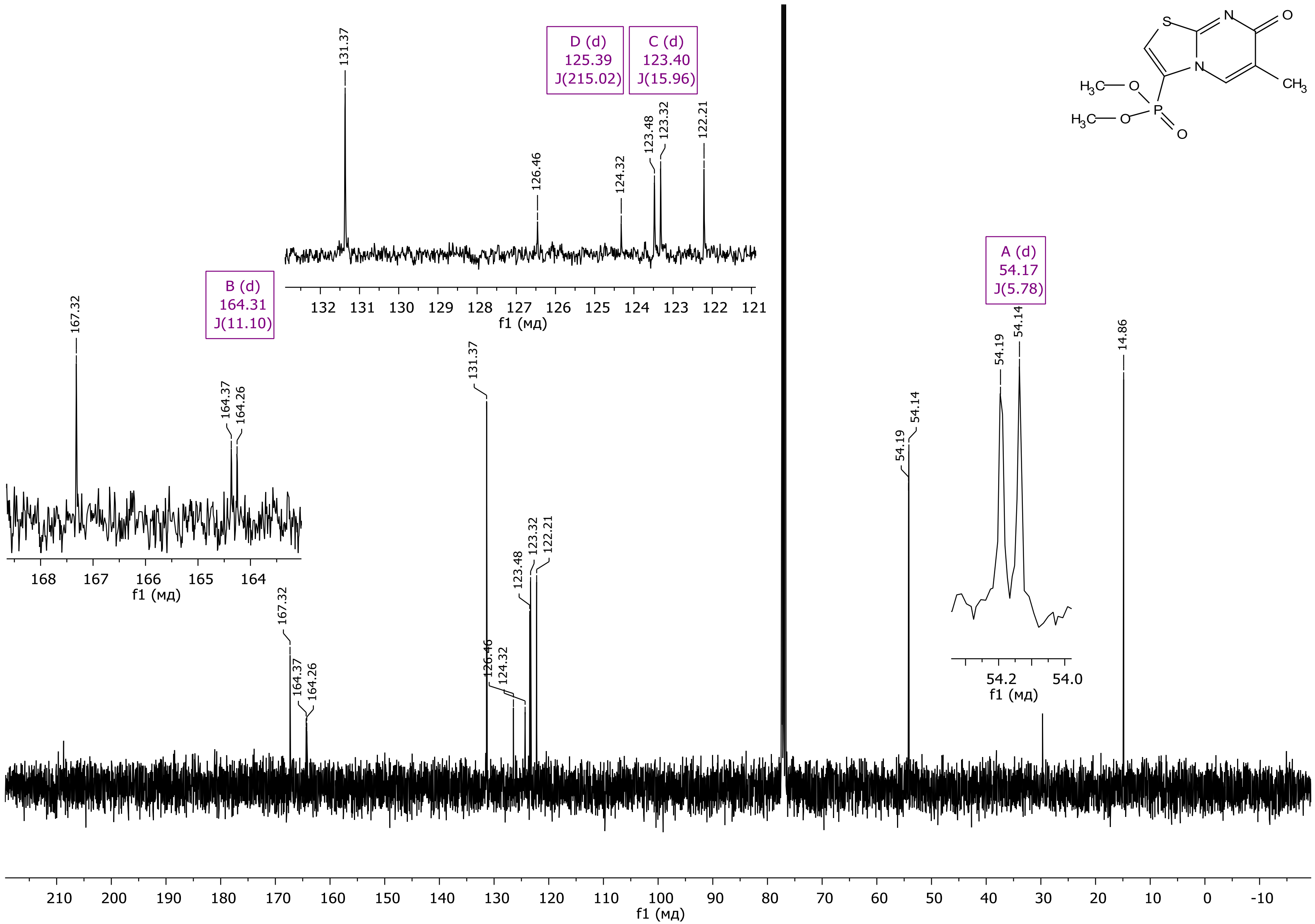

^{13}C NMR spectrum of compound **4a**

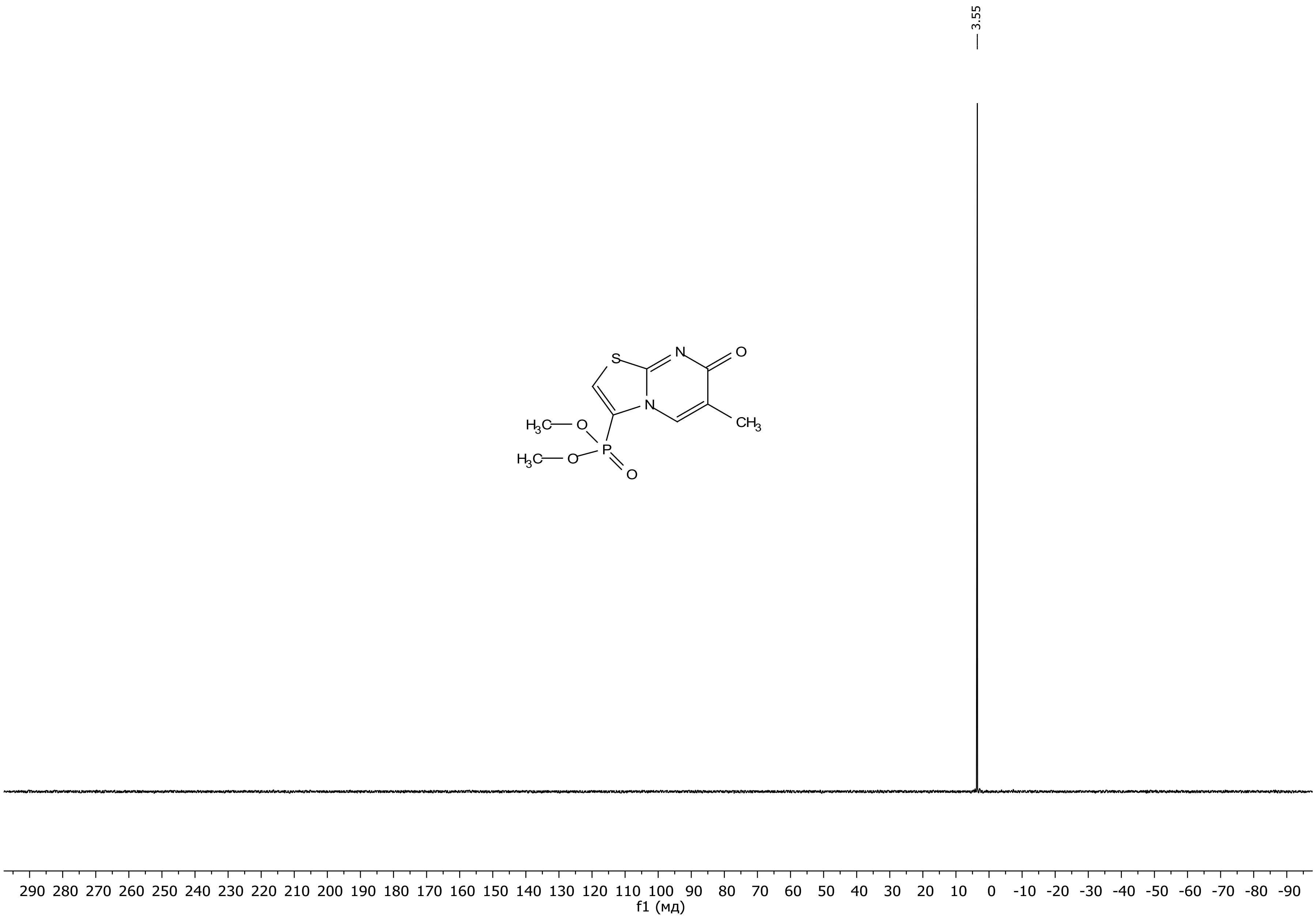

^{31}P NMR spectrum of compound **4a**

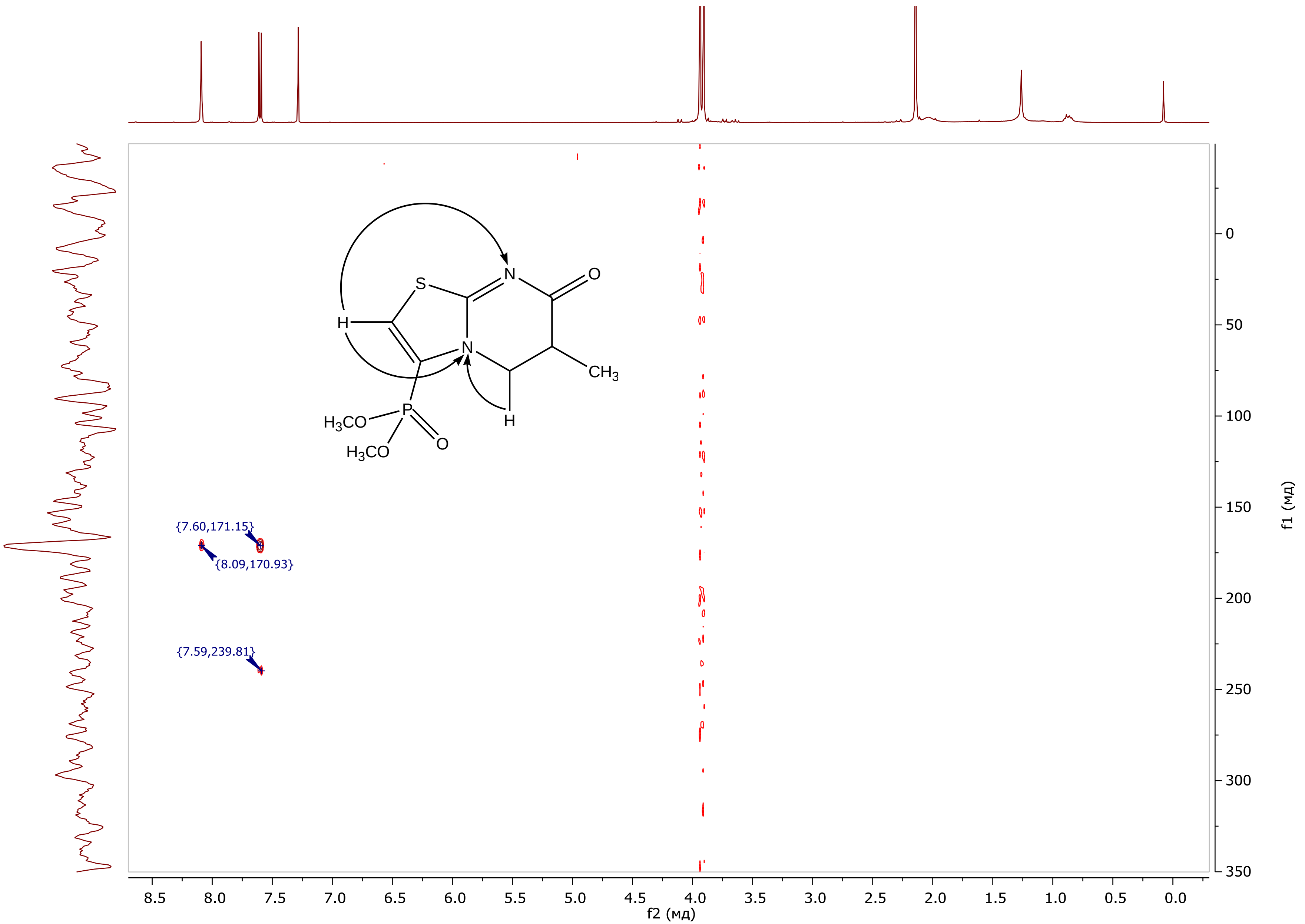

HMBC ^{1}H-^{15}N NMR spectrum of compound **4a**

Diethyl (6-methyl-7-oxo-7*H*-[1,3]thiazolo[3,2-*a*]pyrimidin-3-yl)phosphonate (4b)

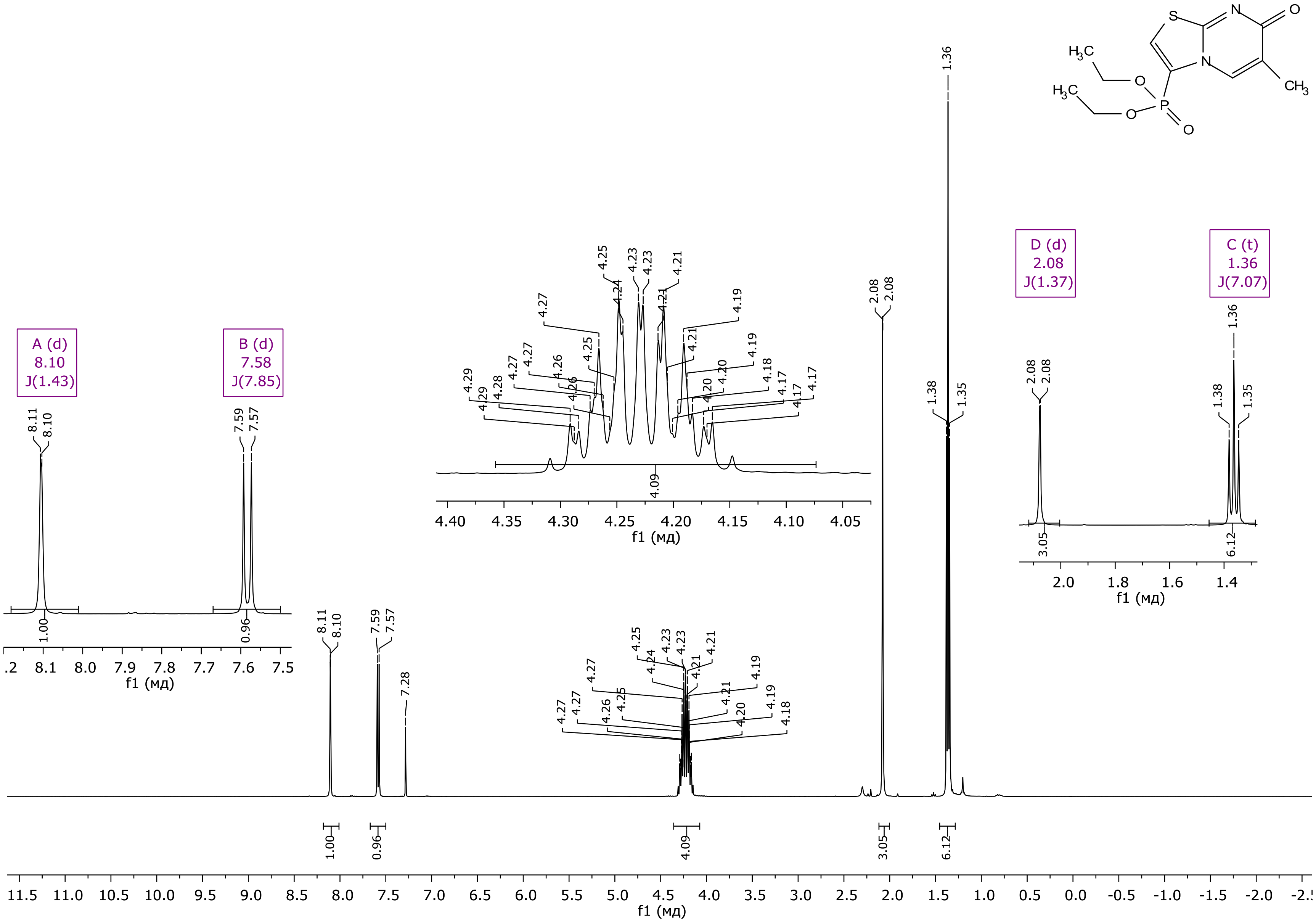

^{1}H NMR spectrum of compound **4b**

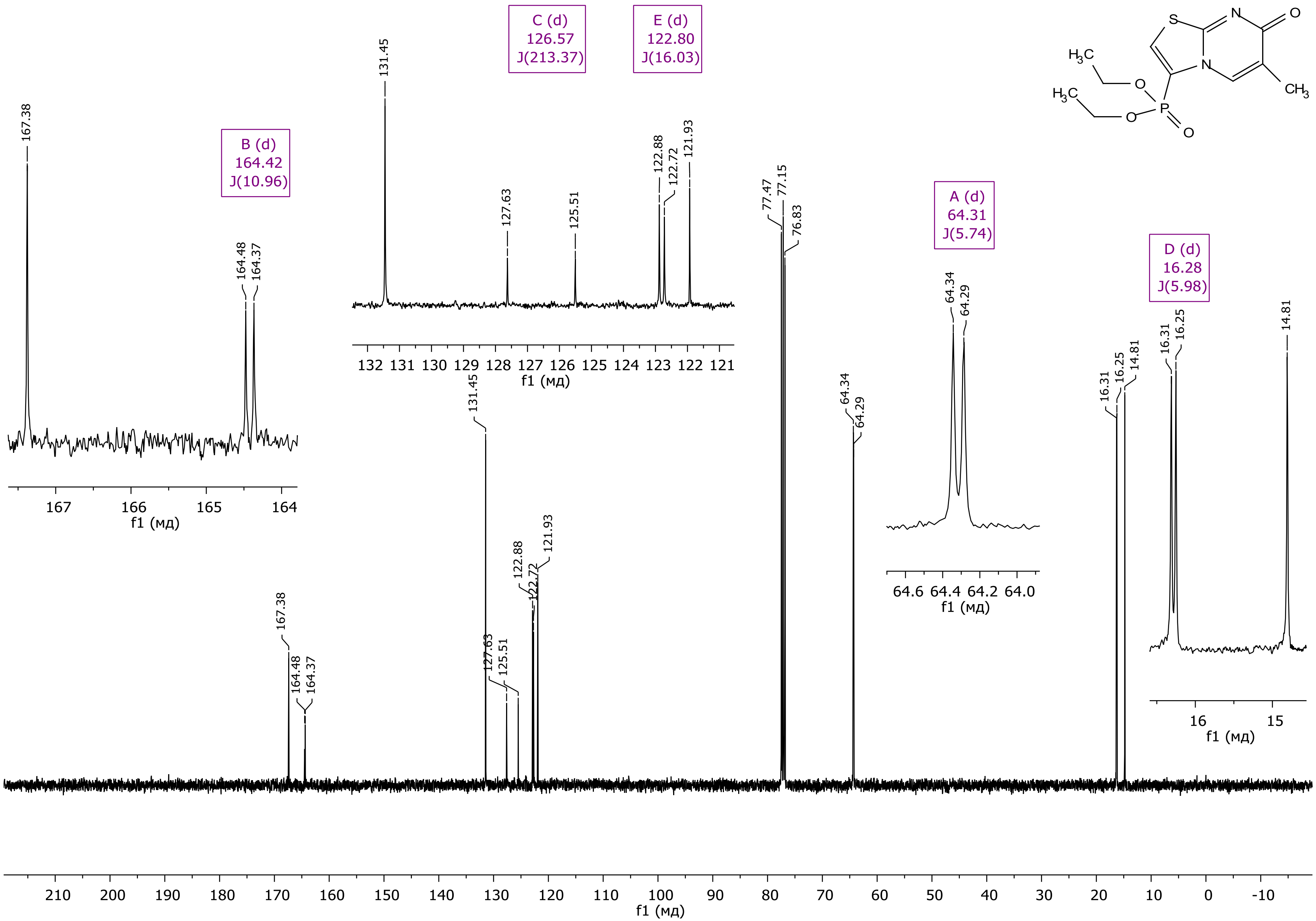

^{13}C NMR spectrum of compound **4b**

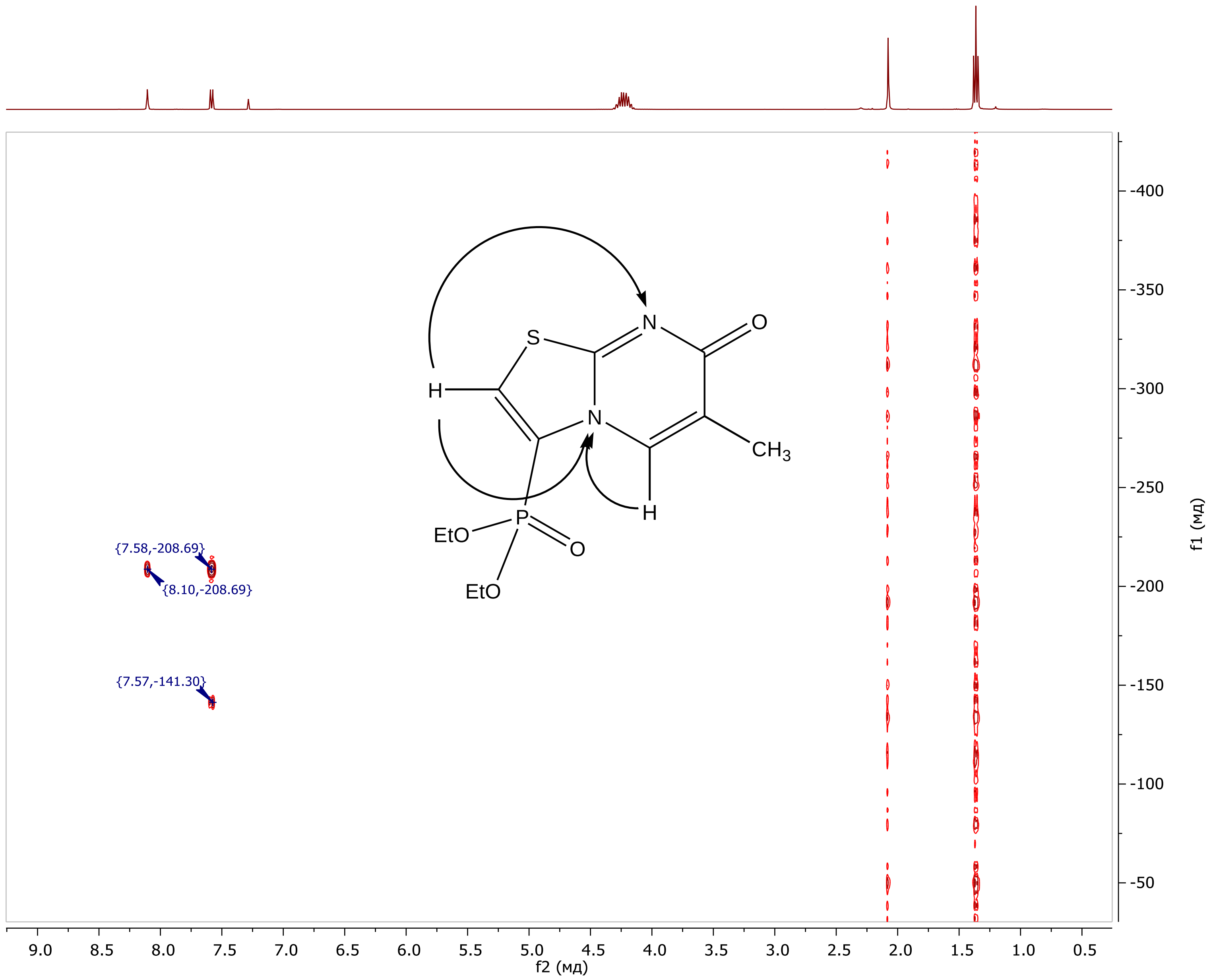

HMBC ^{1}H-^{15}N NMR spectrum of compound **4b**

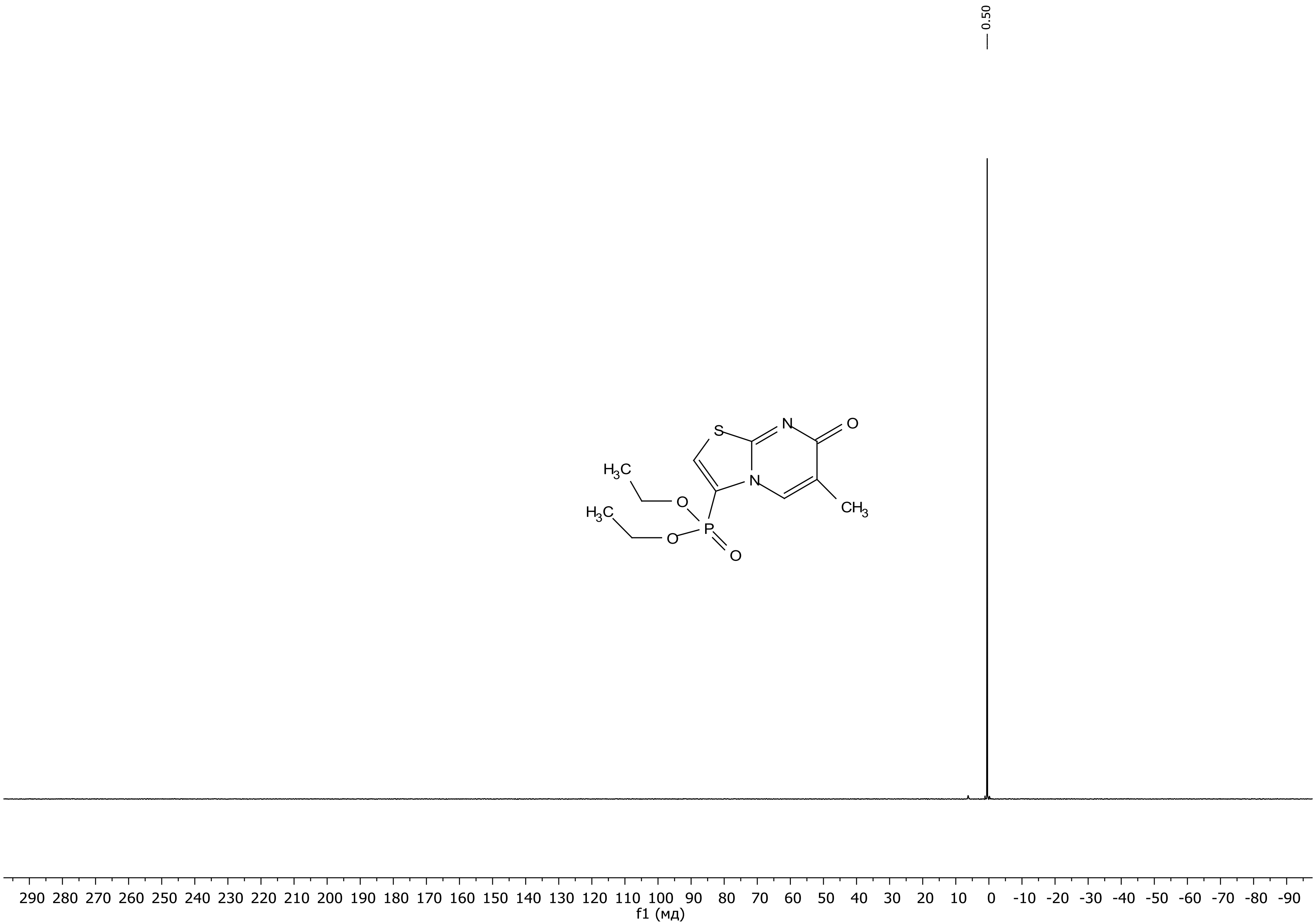

^{31}P NMR spectrum of compound **4b**

Diisopropyl (6-methyl-7-oxo-7*H*-[1,3]thiazolo[3,2-*a*]pyrimidin-3-yl)phosphonate (4c)

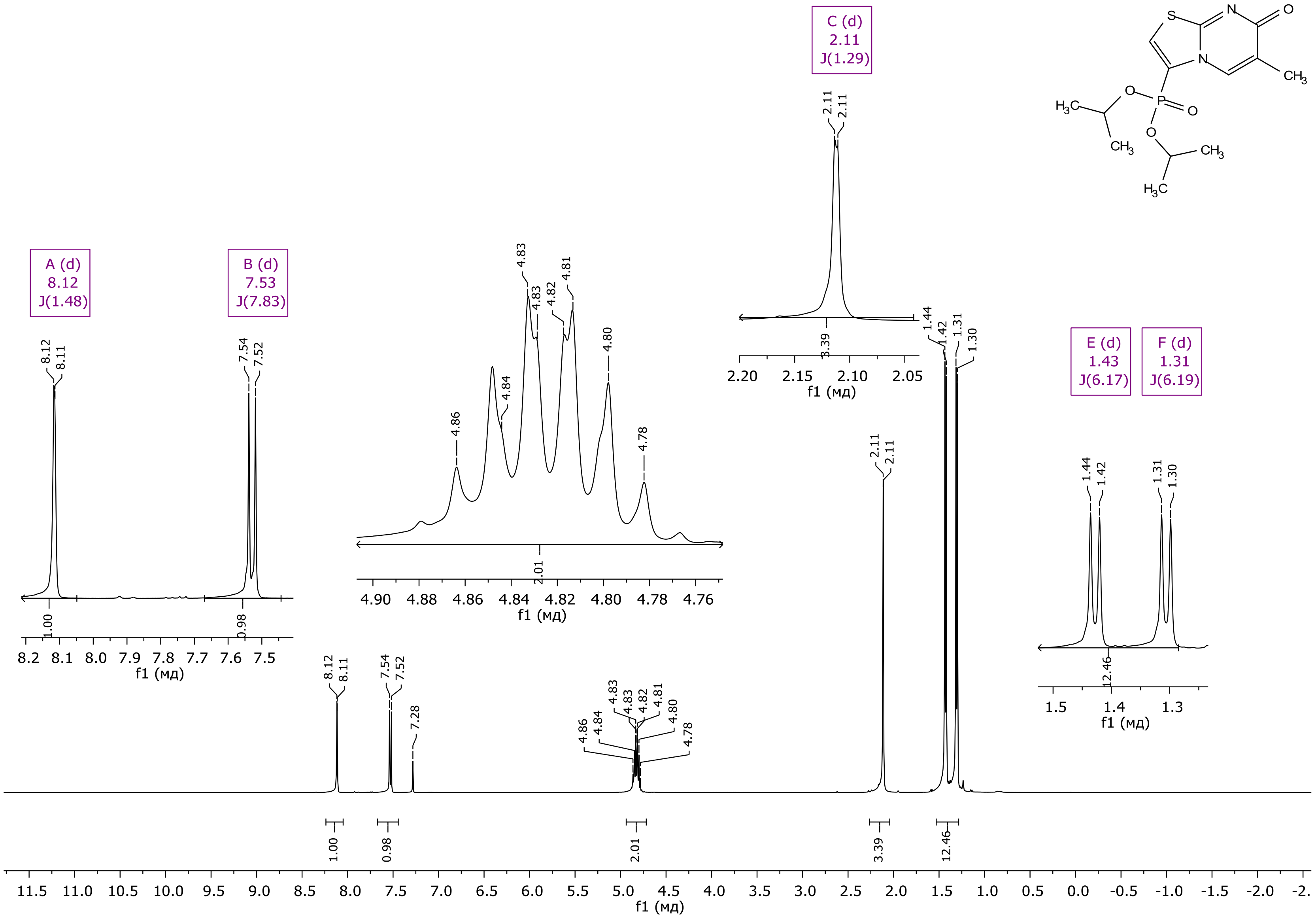

^{1}H NMR spectrum of compound **4c**

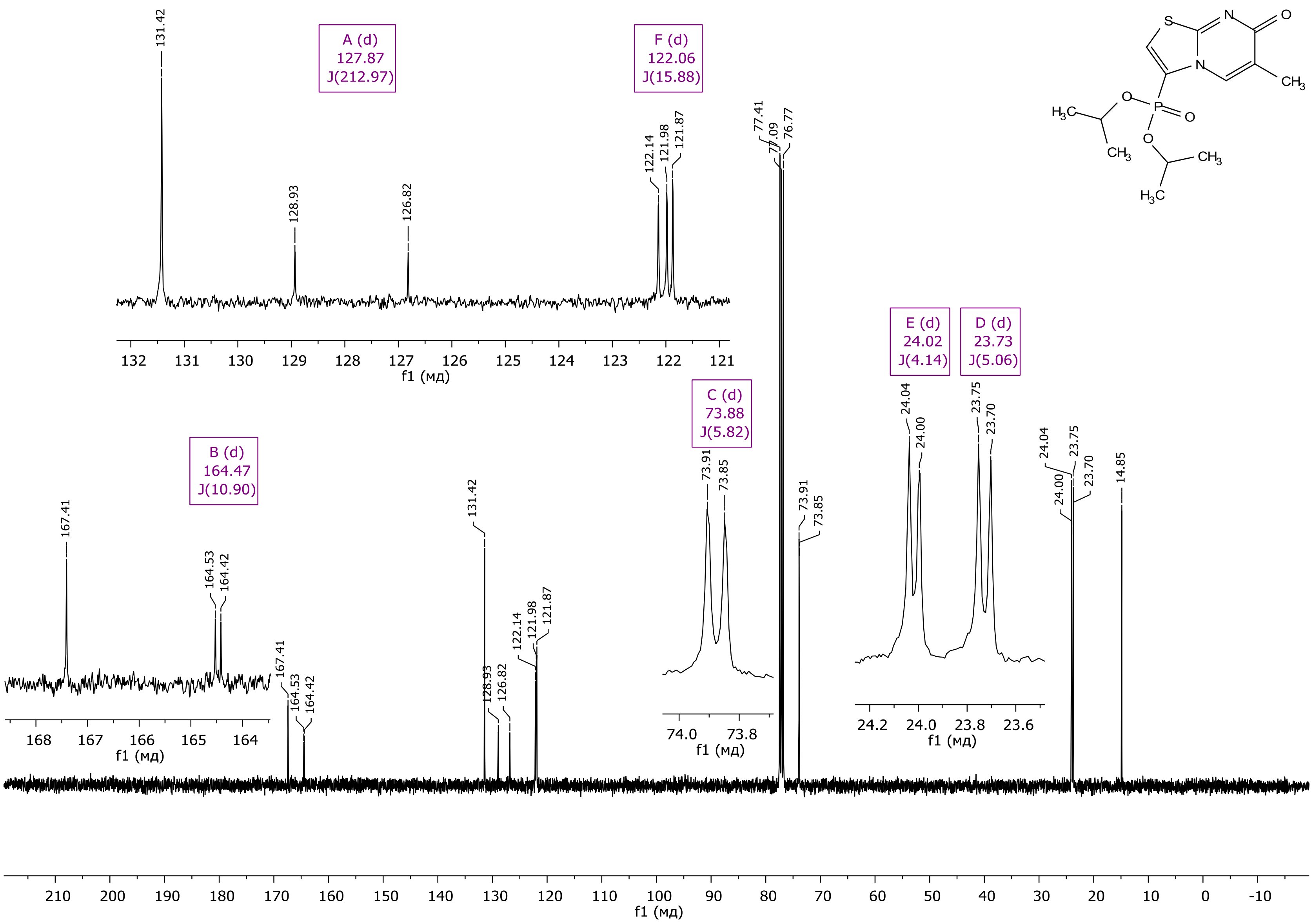

^{13}C NMR spectrum of compound **4c**

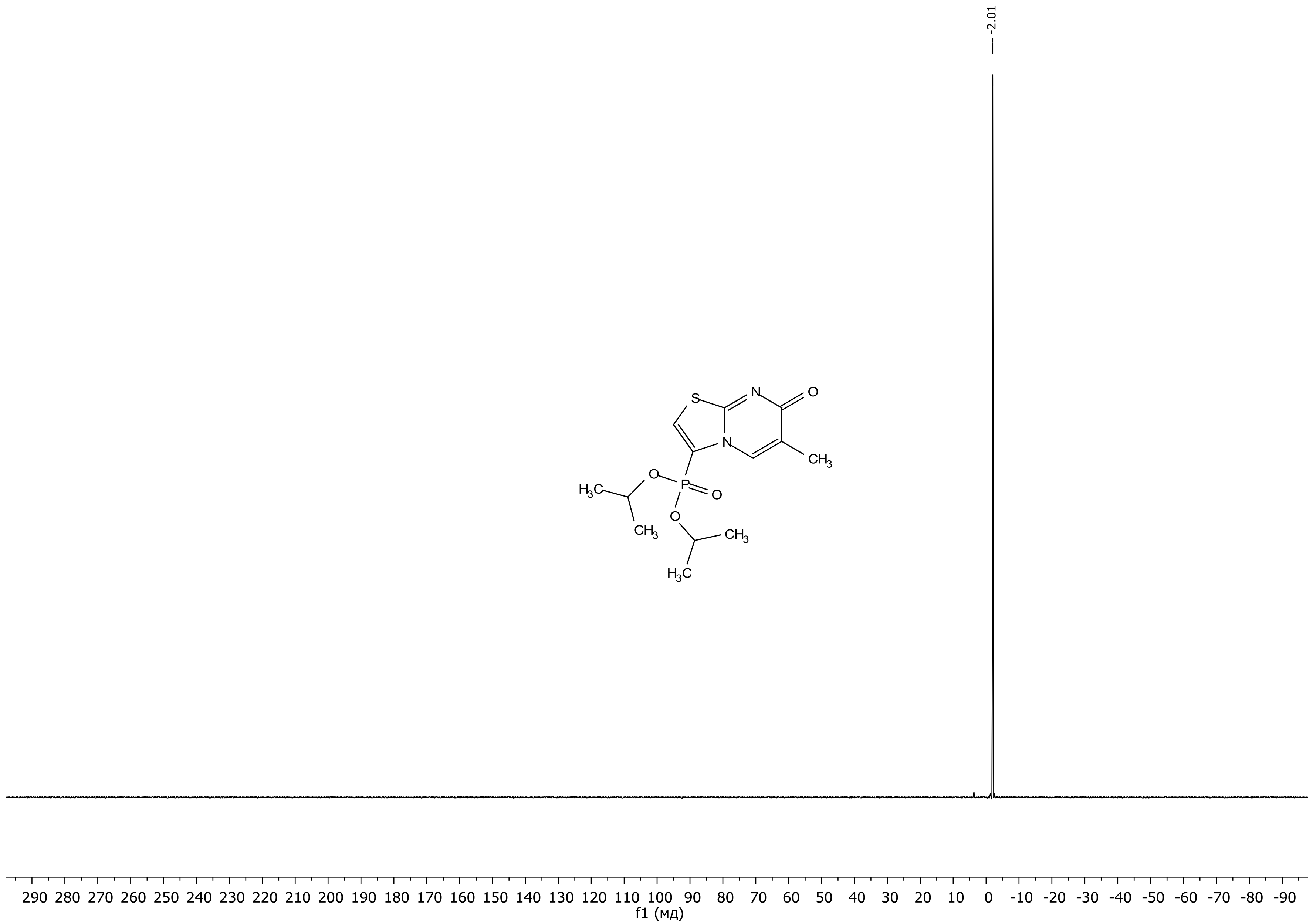

^{31}P NMR spectrum of compound **4c**

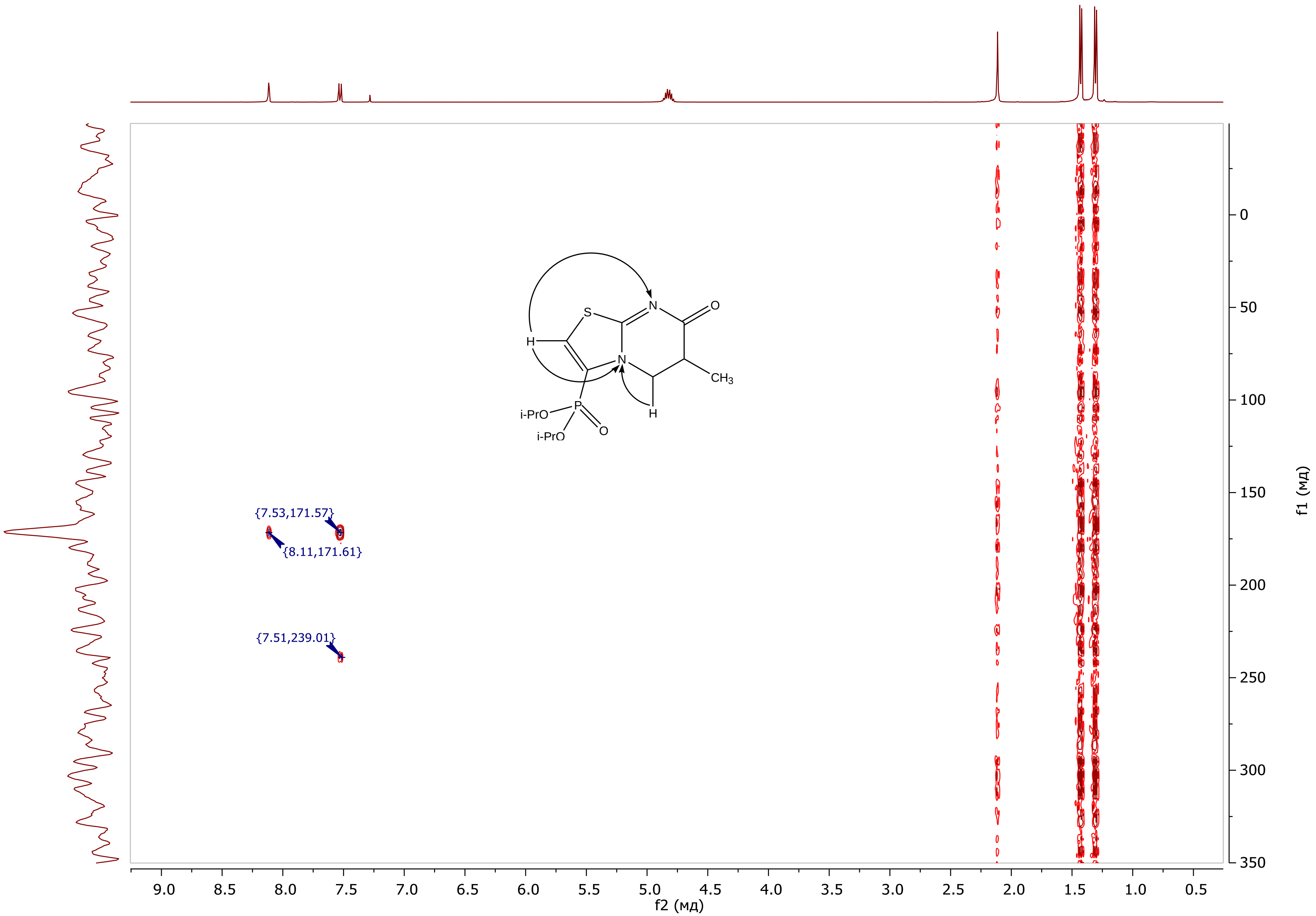

HMBC ^{1}H-^{15}N NMR spectrum of compound **4c**

(7-Oxo-7*H*-thiazolo[3,2-*a*]pyrimidin-3-yl)phosphonic acid monomethyl ester (6aa)

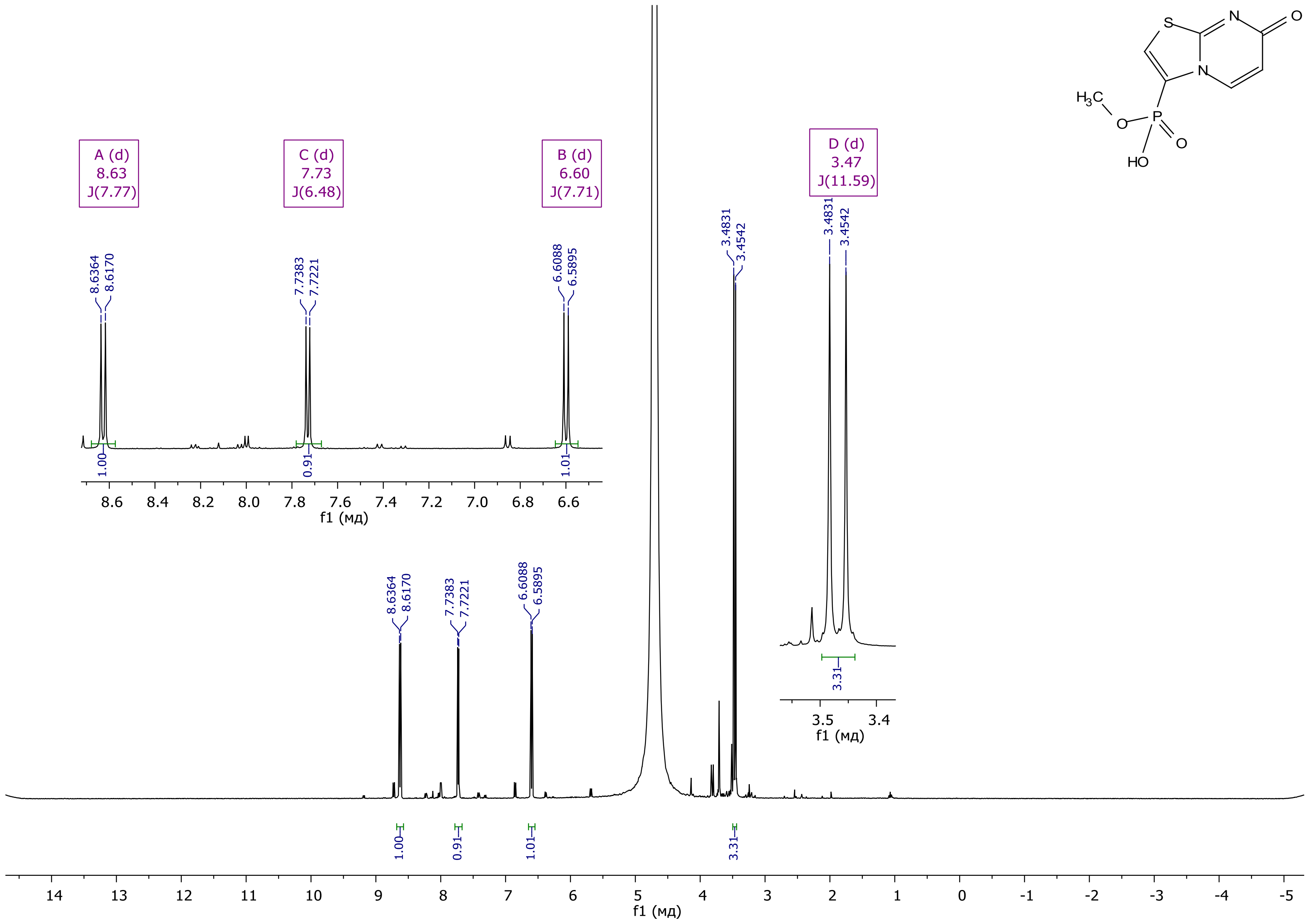

^{1}H NMR spectrum of compound **6aa**

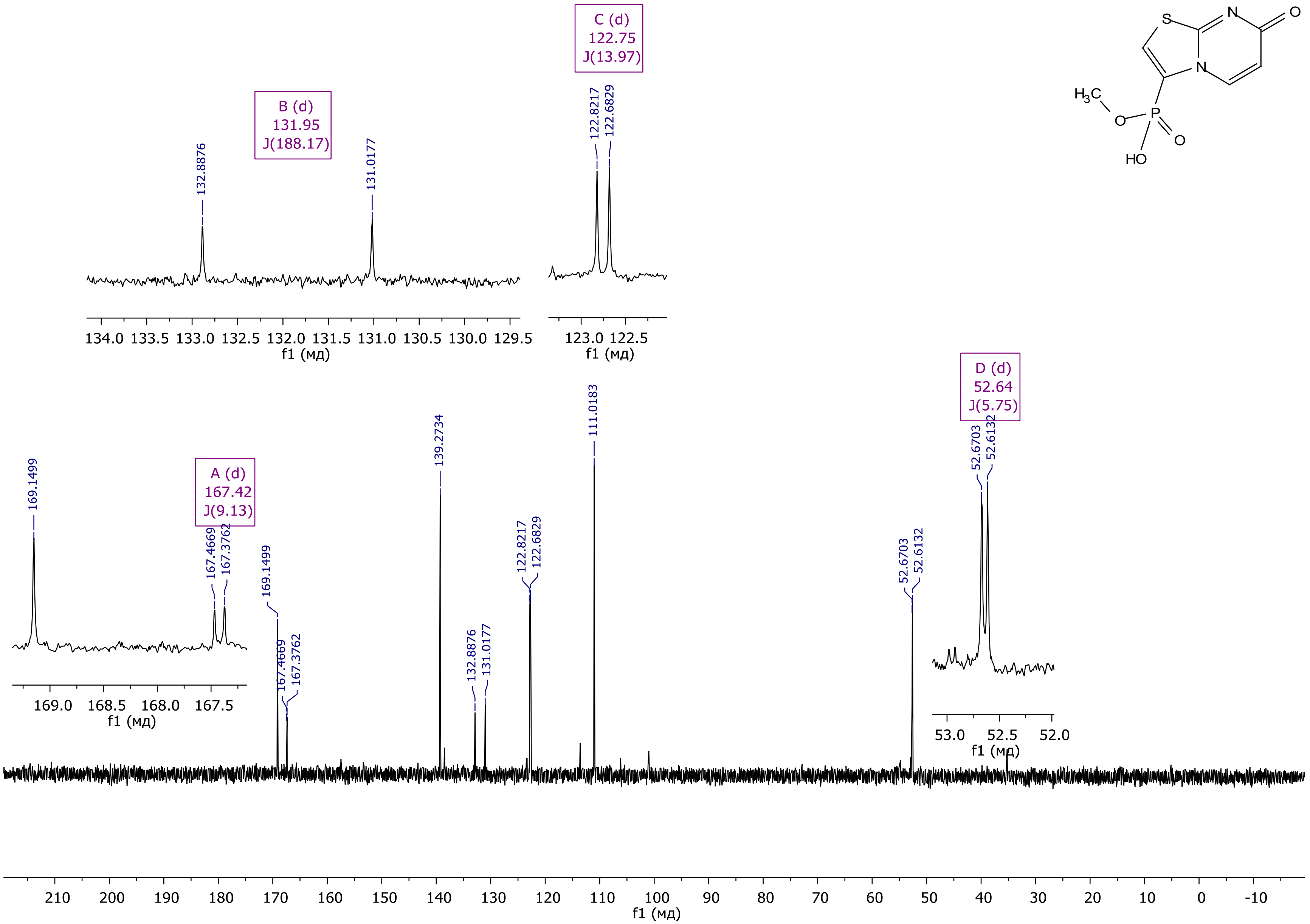

^{13}C NMR spectrum of compound **6aa**

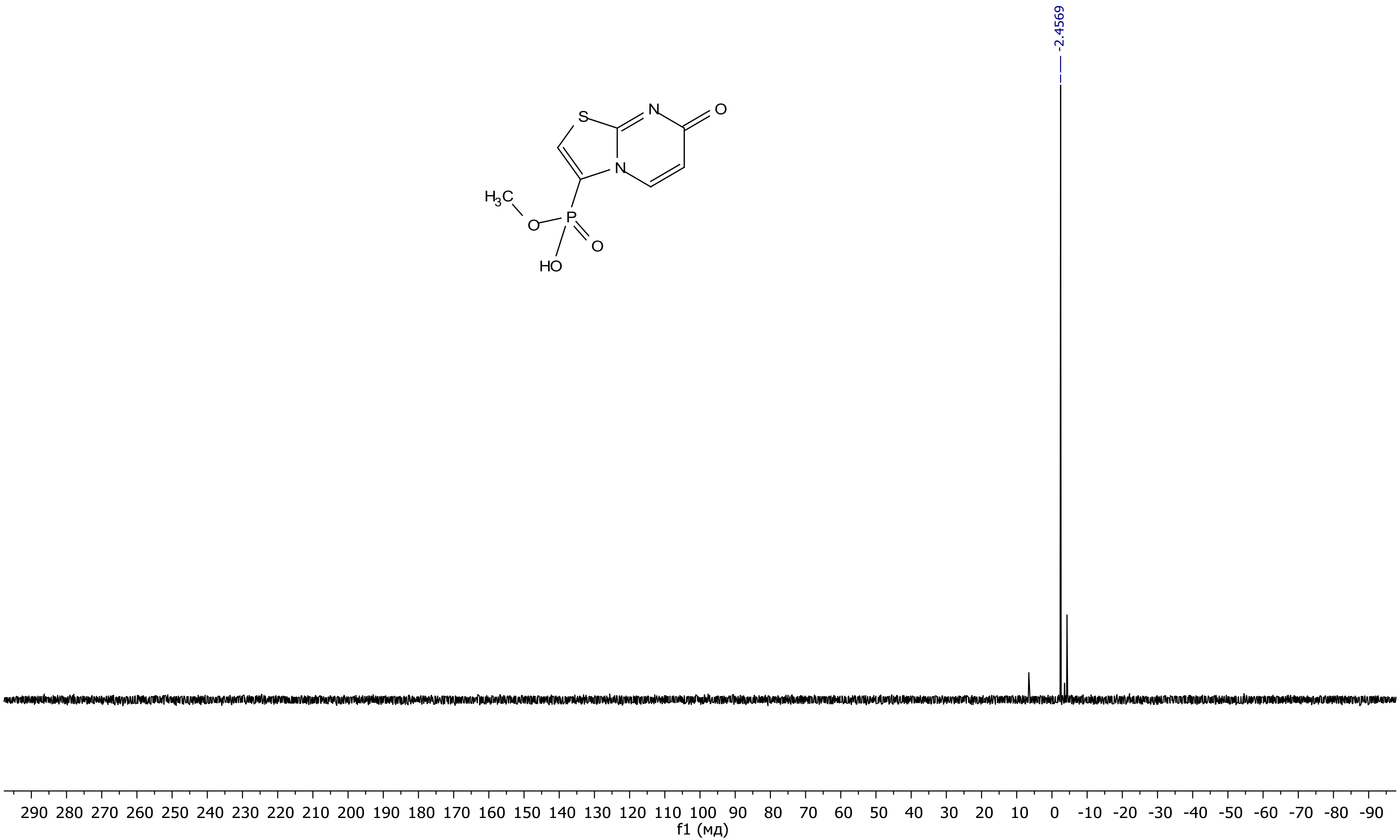

^{31}P NMR spectrum of compound **6aa**

Diethyl (7-oxo-7*H*-thiazolo[3,2-*a*]pyrimidin-3-yl)phosphonate (6b)

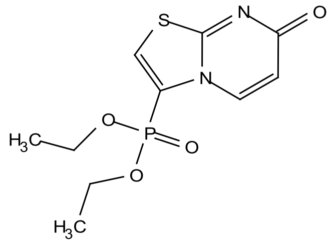

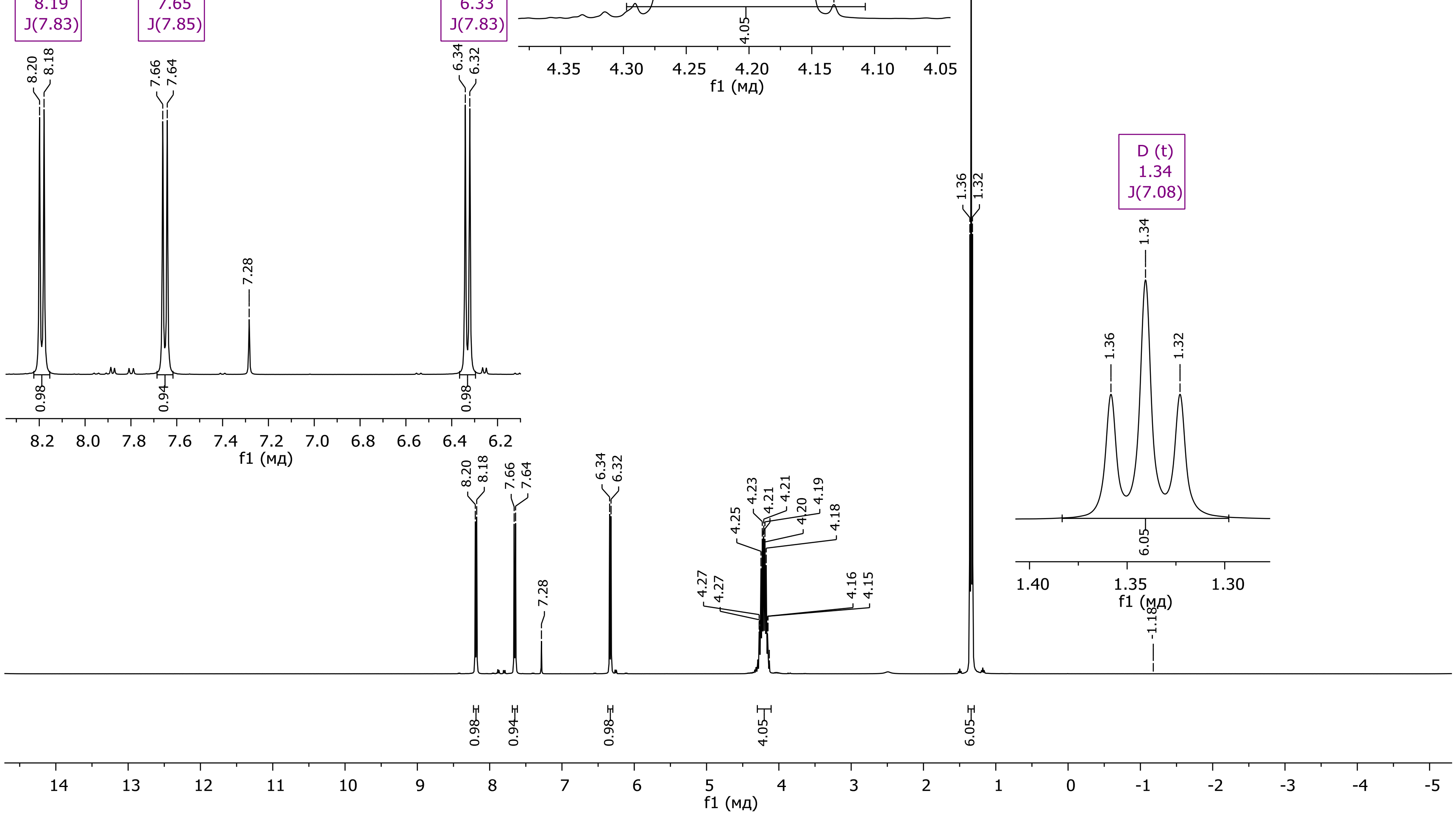

^{1}H NMR spectrum of compound **6b**

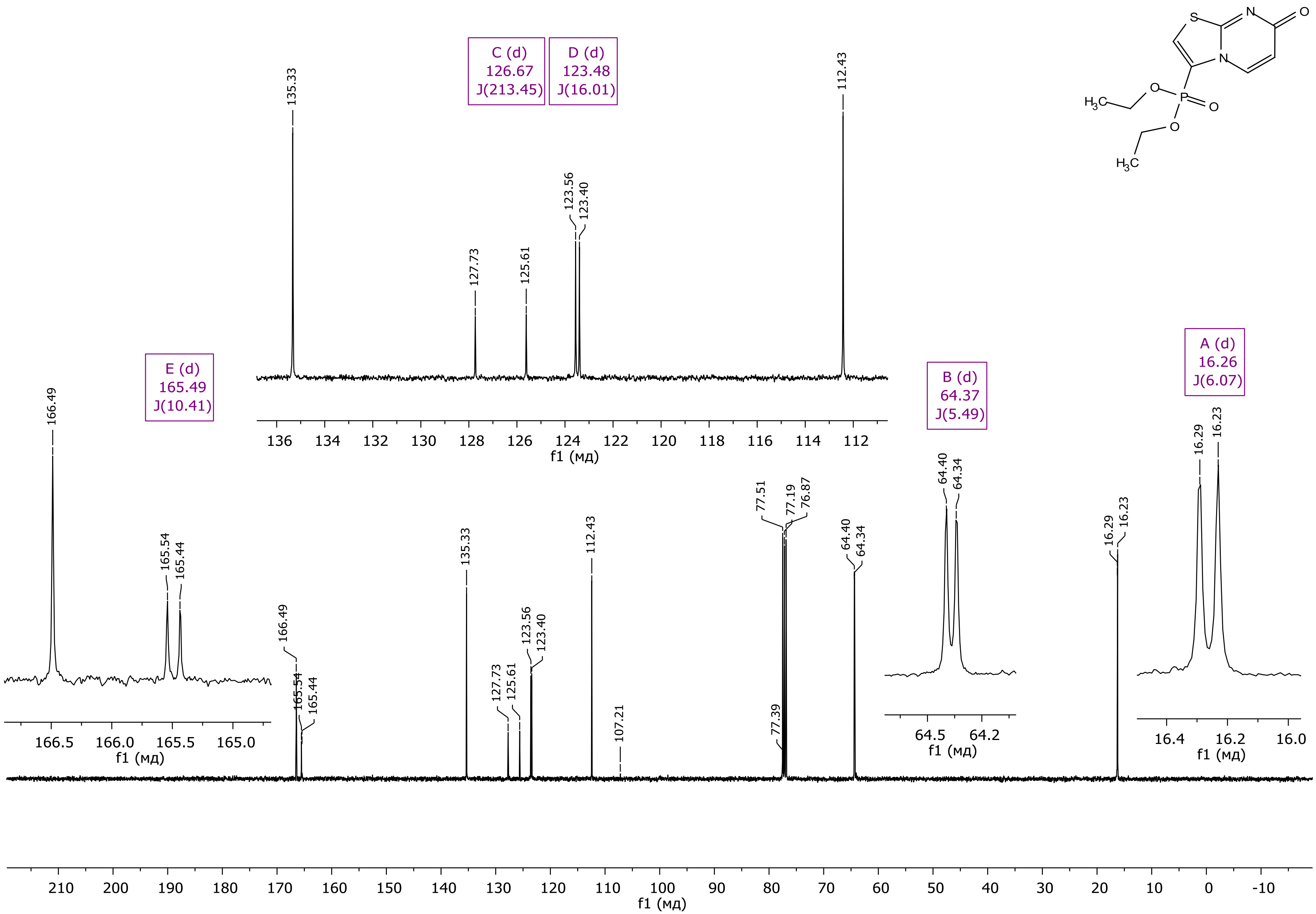

^{13}C NMR spectrum of compound **6b**

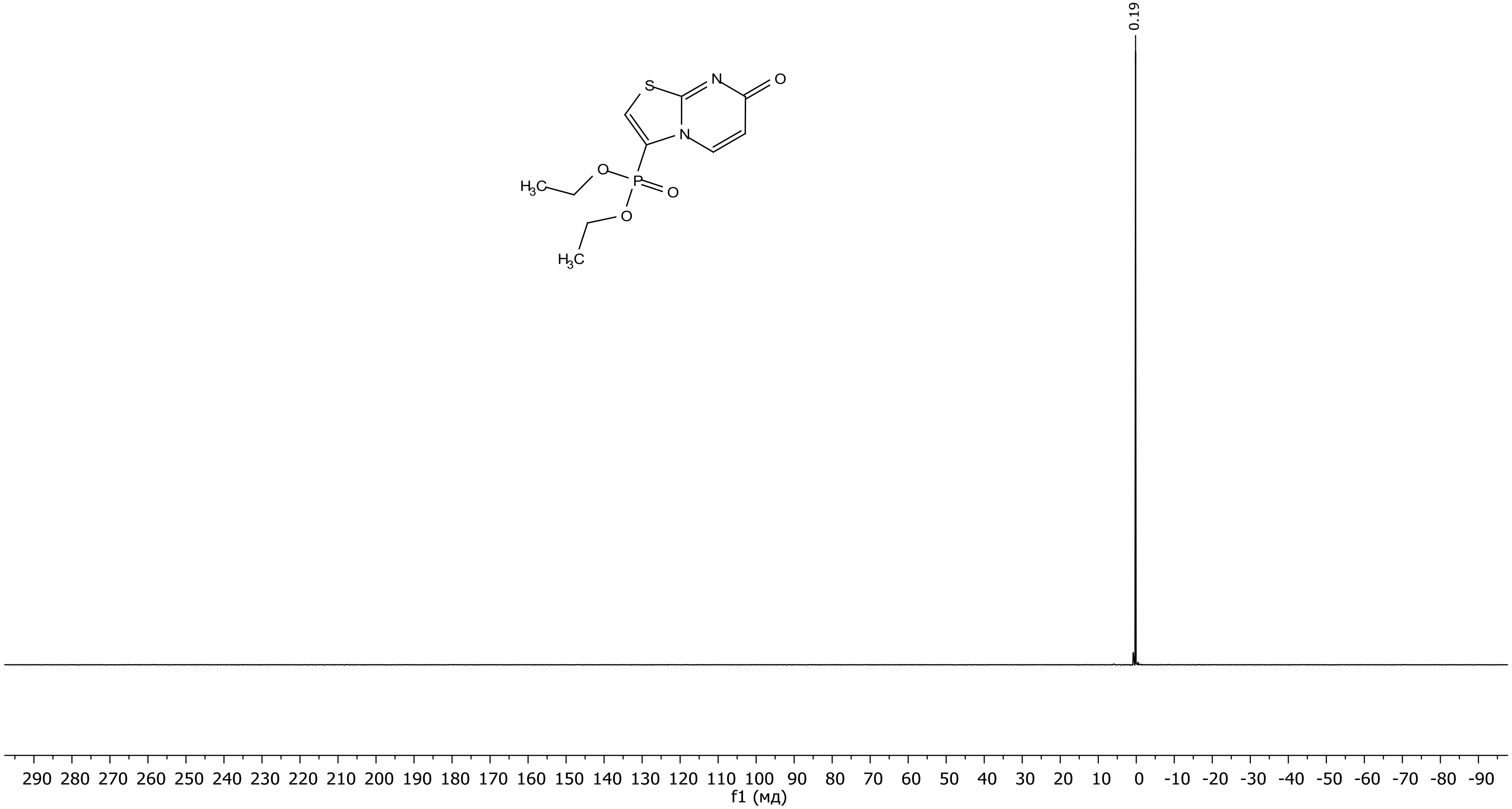

^{31}P NMR spectrum of compound **6b**

Diisopropyl (7-oxo-7*H*-[1,3]thiazolo[3,2-*a*]pyrimidin-3-yl)phosphonate (6c)

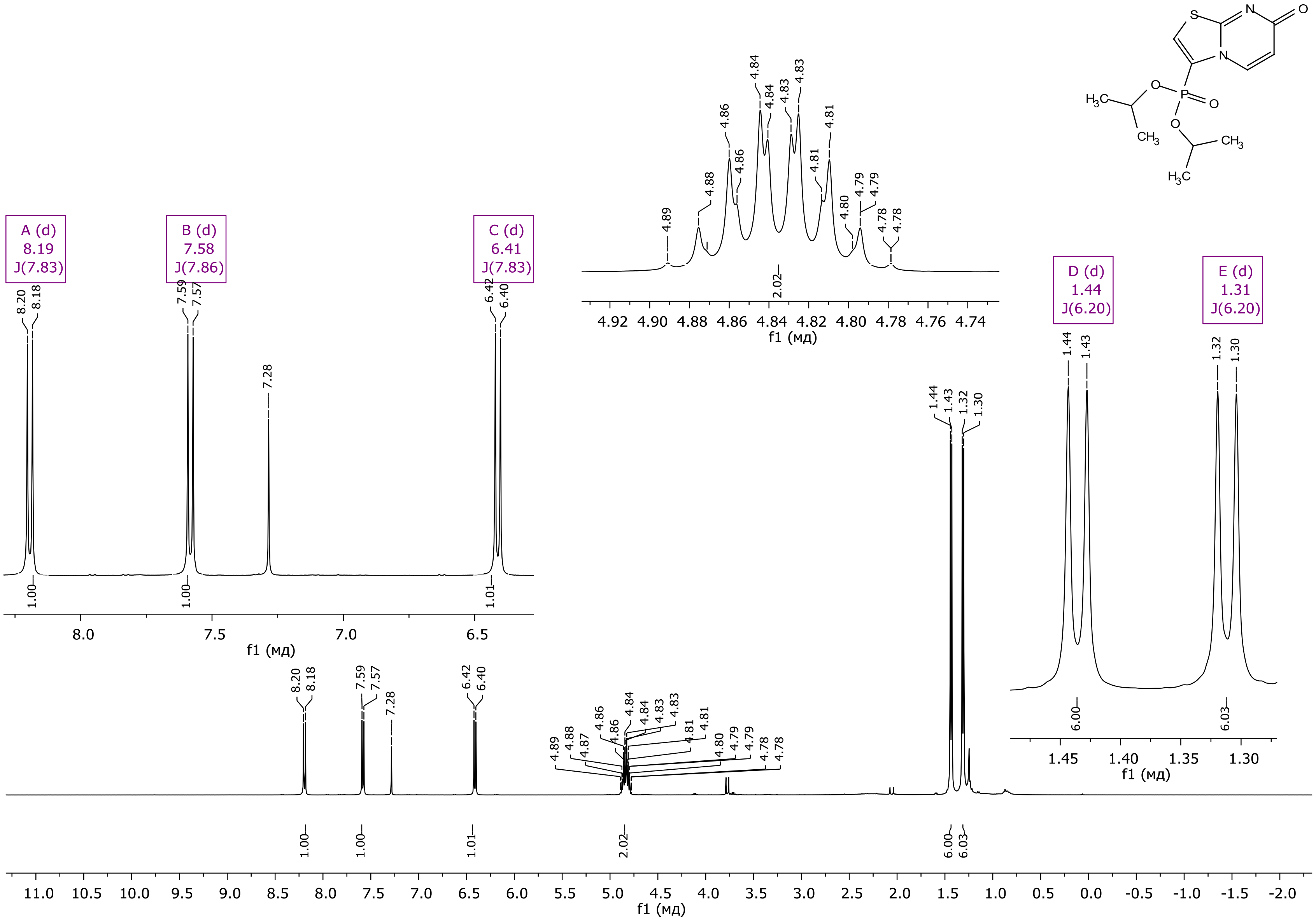

^{1}H NMR spectrum of compound **6c**

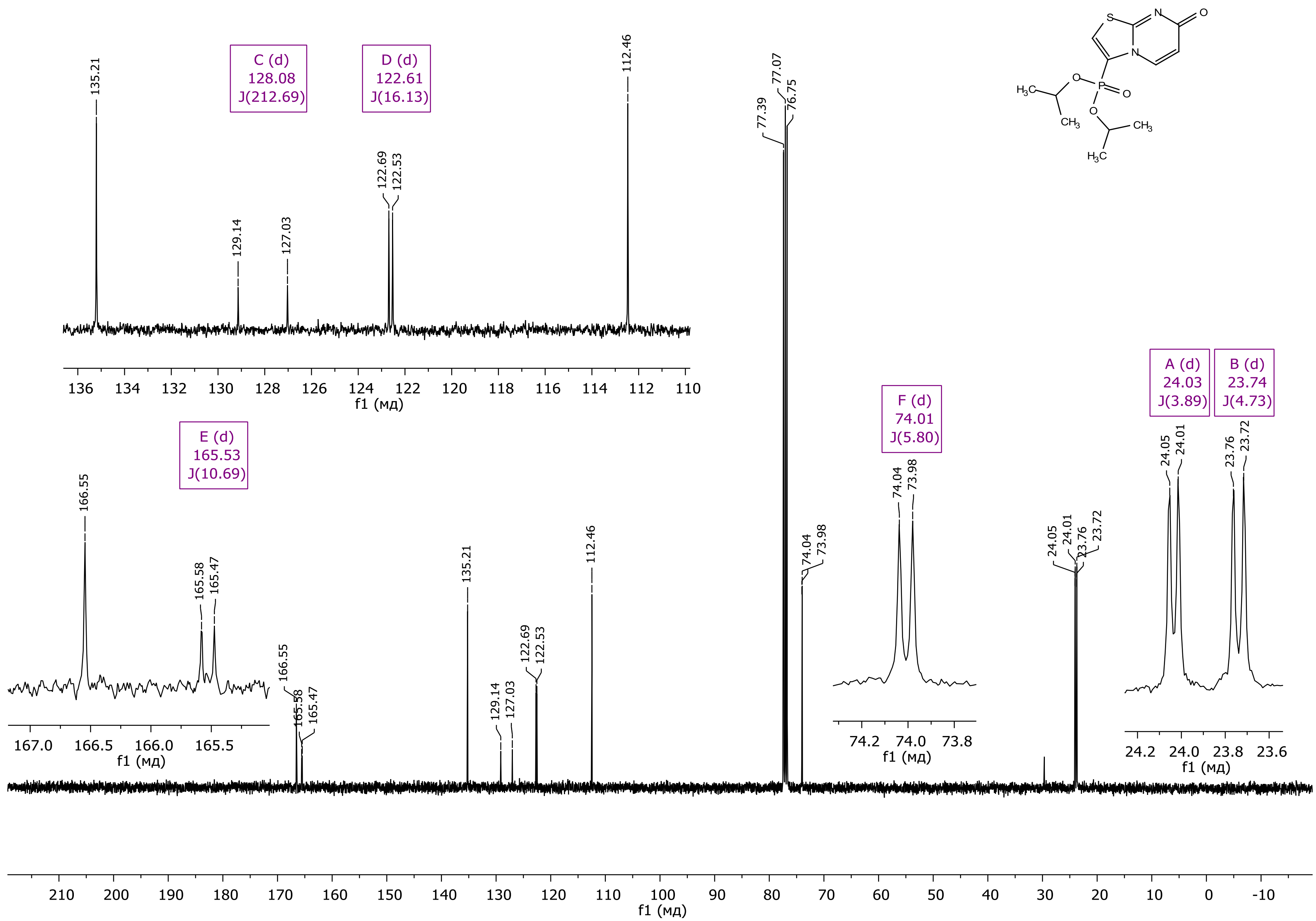

^{13}C NMR spectrum of compound **6c**

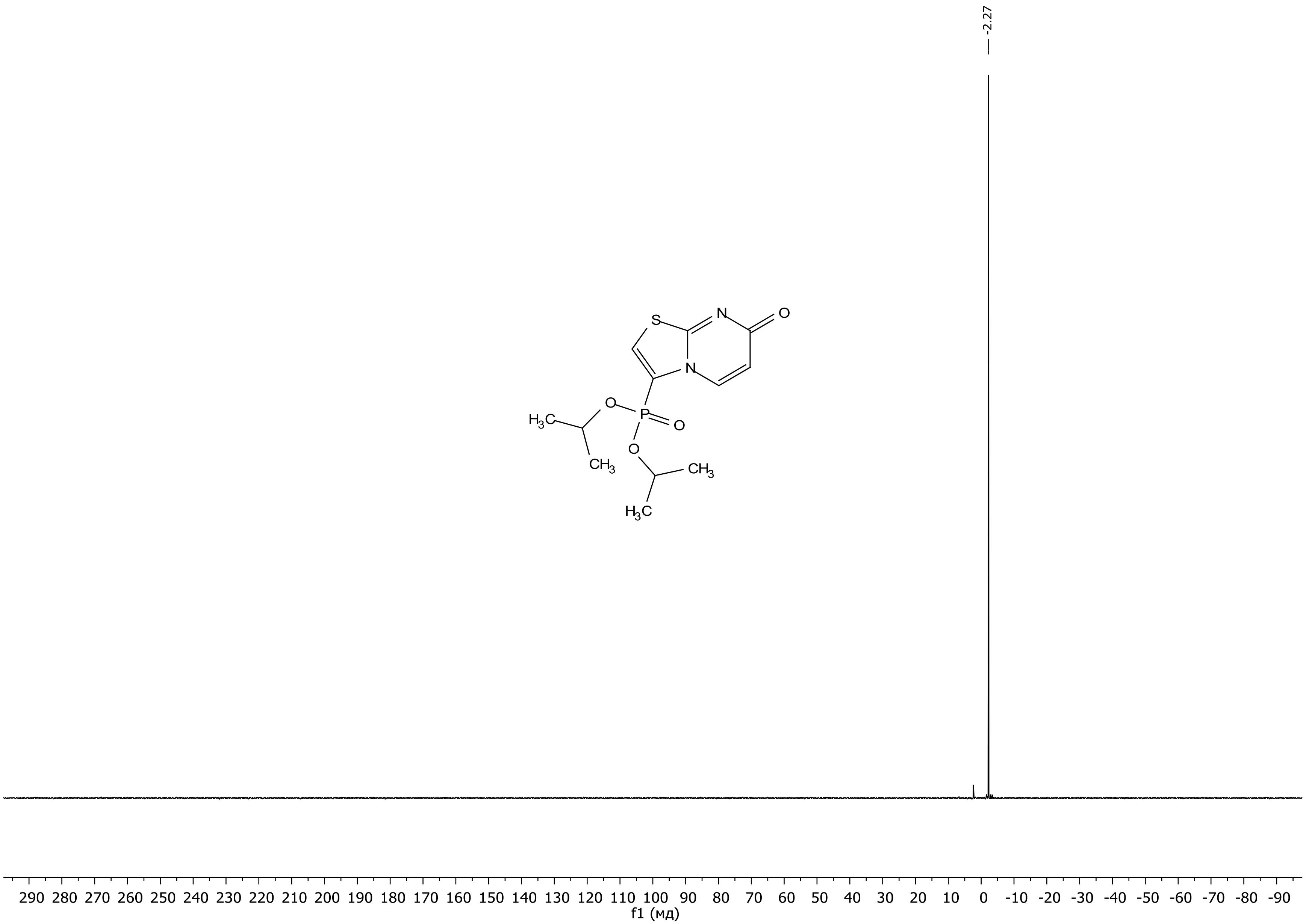

^{31}P NMR spectrum of compound **6c**

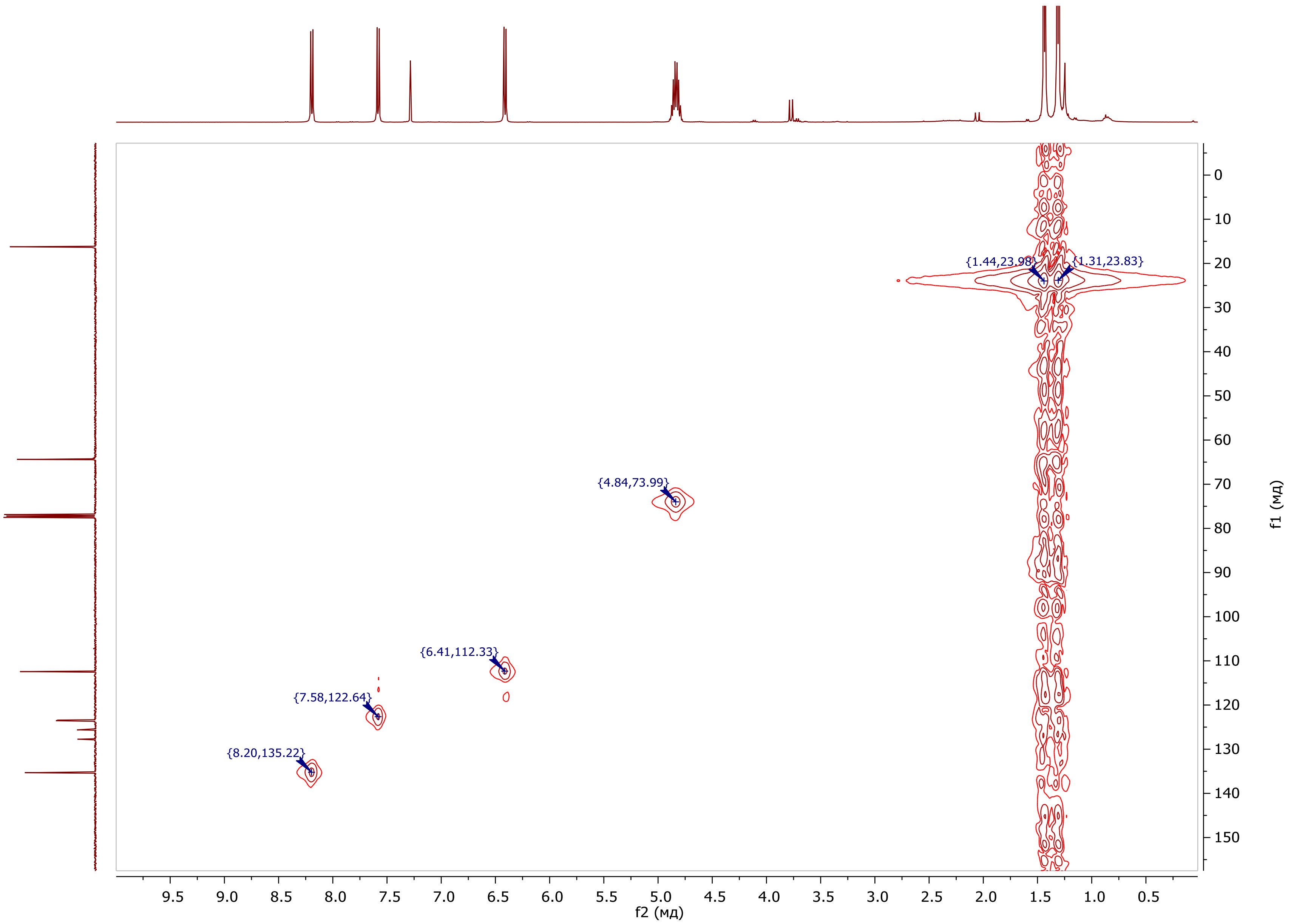

HSQC ^{1}H-^{13}C NMR spectrum of compound **6c**

Dimethyl (7-oxo-7*H*-thiazolo[2,3-b]pyrimidin-3-yl)phosphonate (6a)

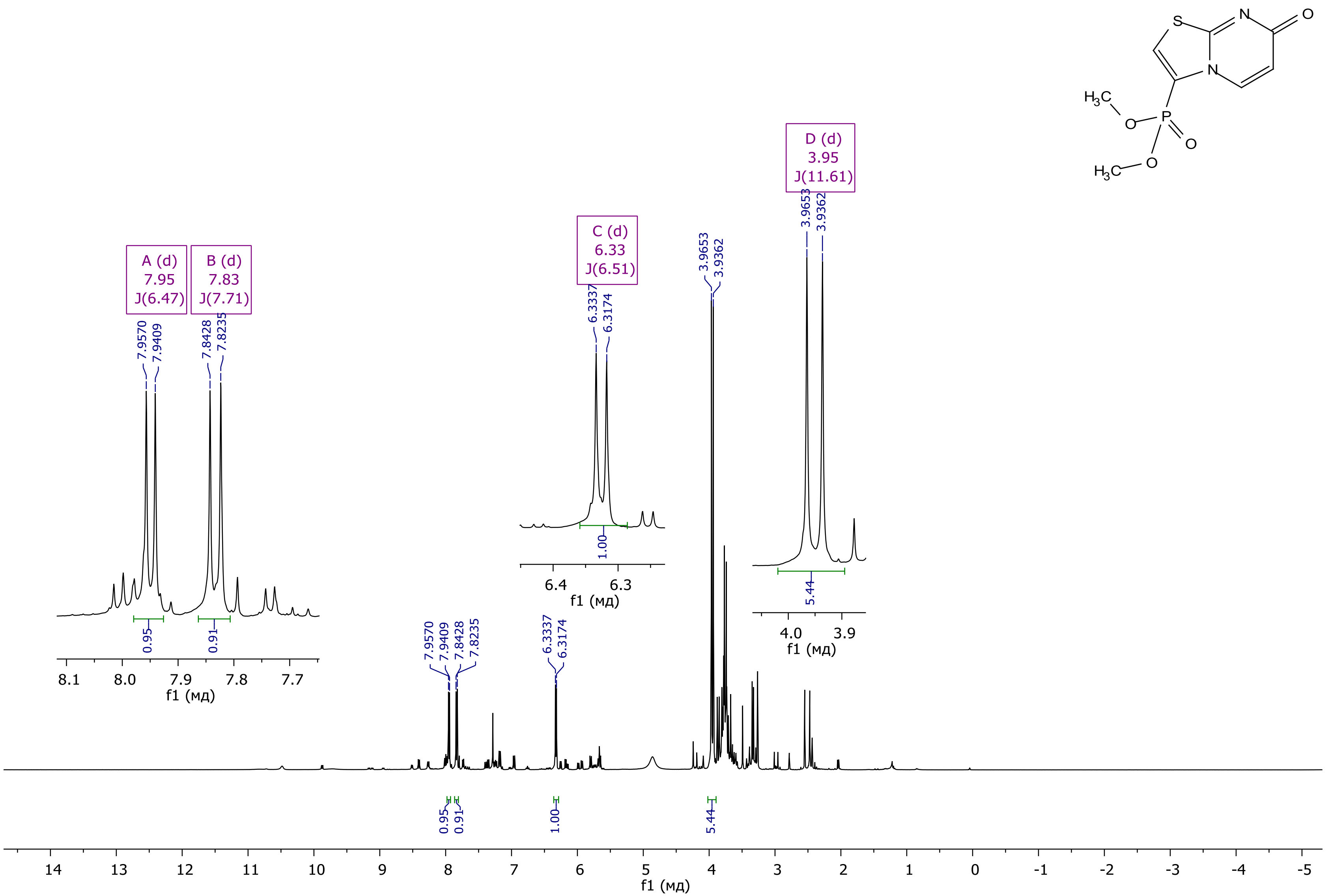

^{1}H NMR spectrum of compound **6a**

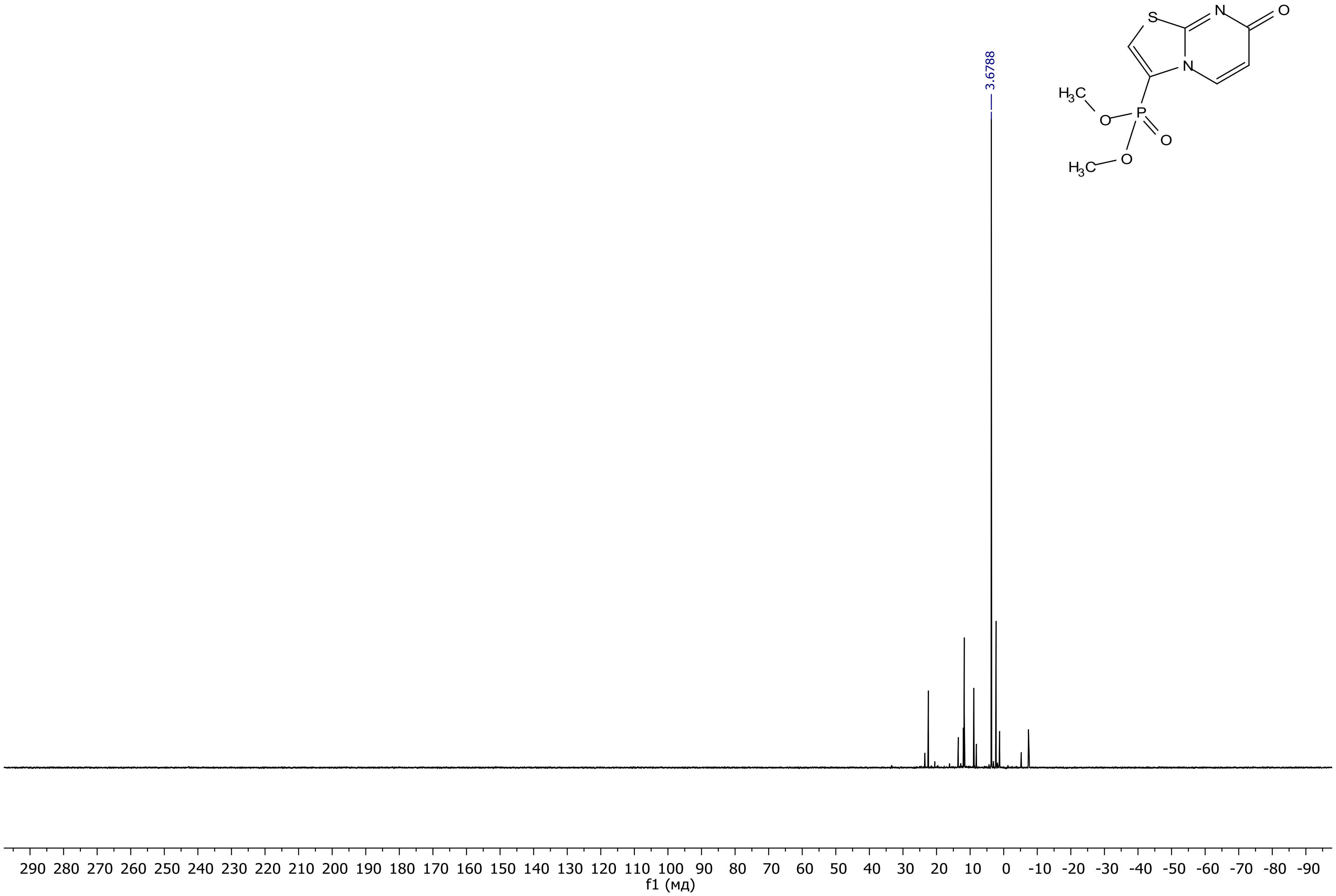

^{31}P NMR spectrum of compound **6a**

Dimethyl [5-oxo-7-(trifluoromethyl)-5*H*-[1,3]thiazolo[3,2-*a*]pyrimidin-3-yl]phosphonate (8a)

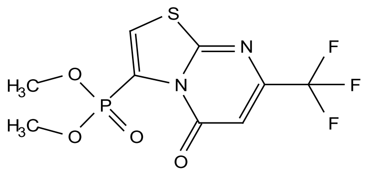

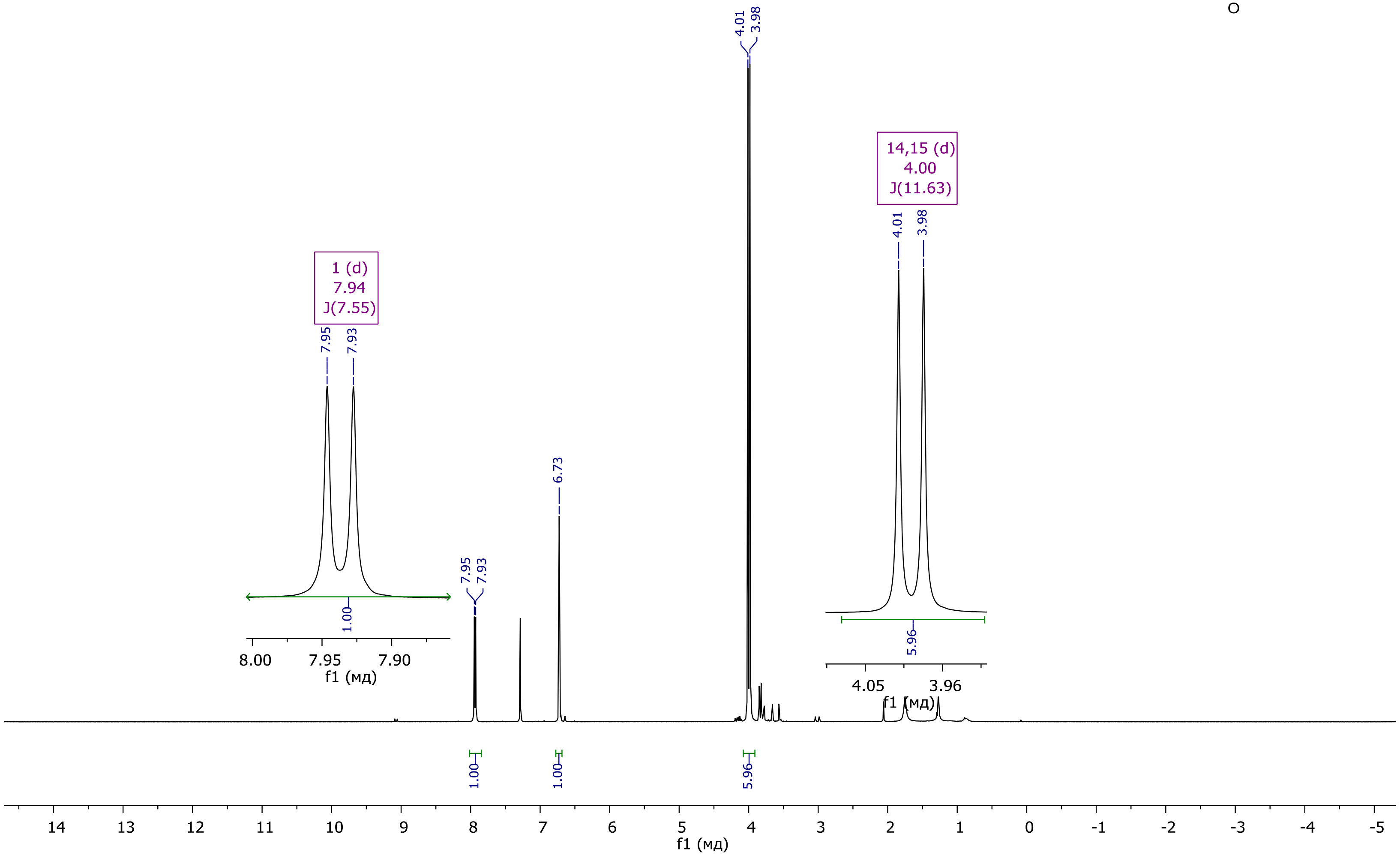

^{1}H NMR spectrum of compound **8a**

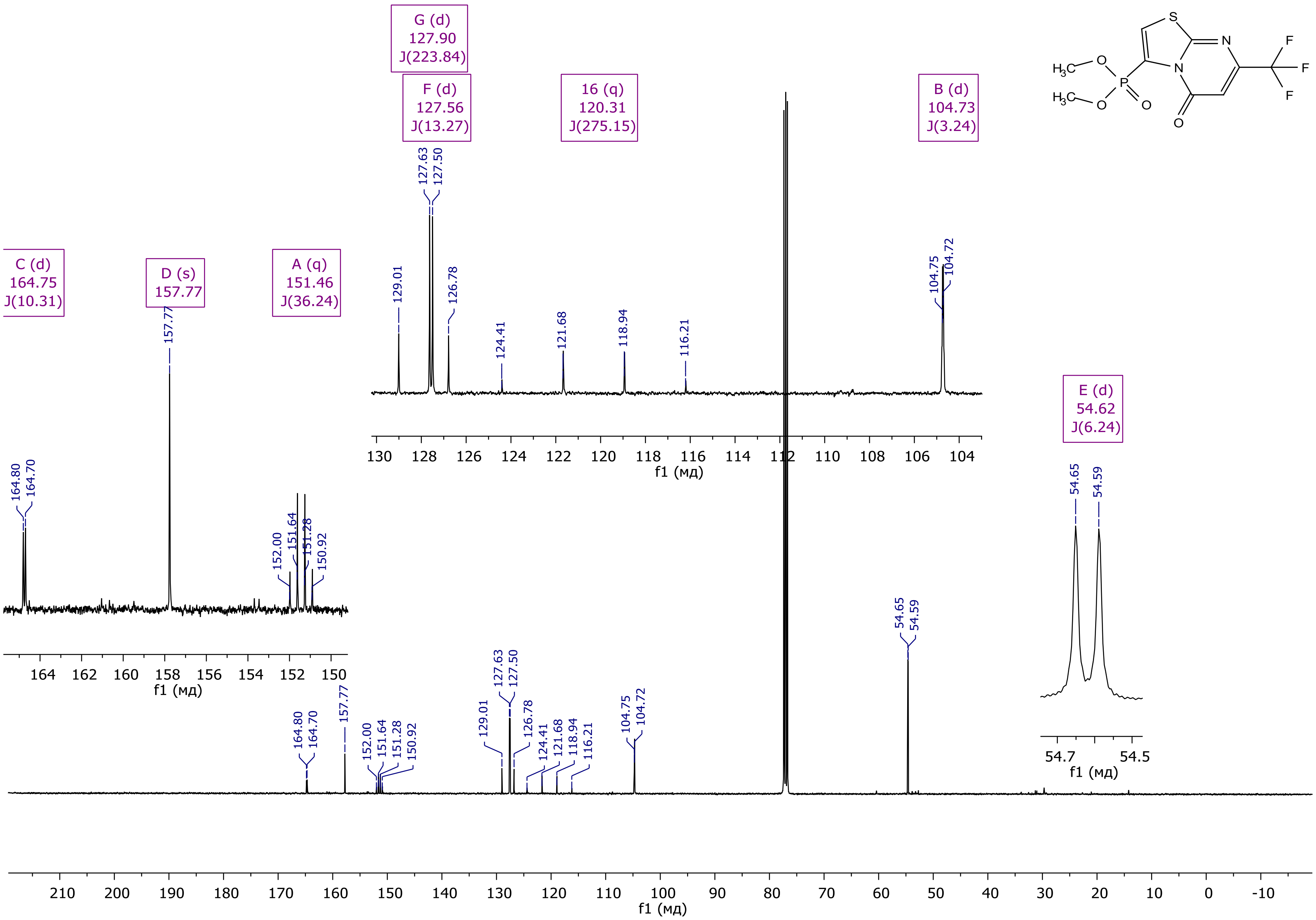

^{13}C NMR spectrum of compound **8a**

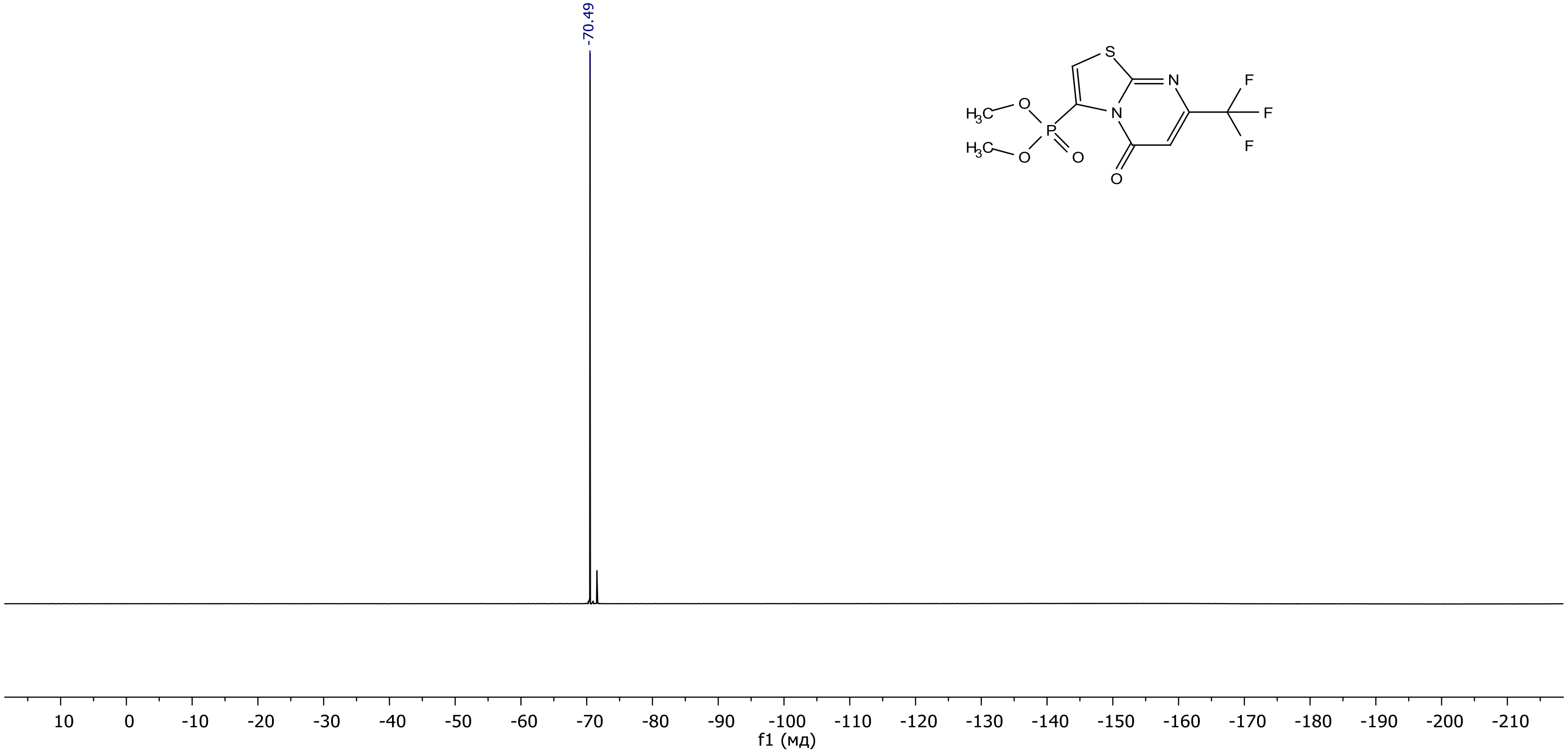

^{19}F NMR spectrum of compound **8a**

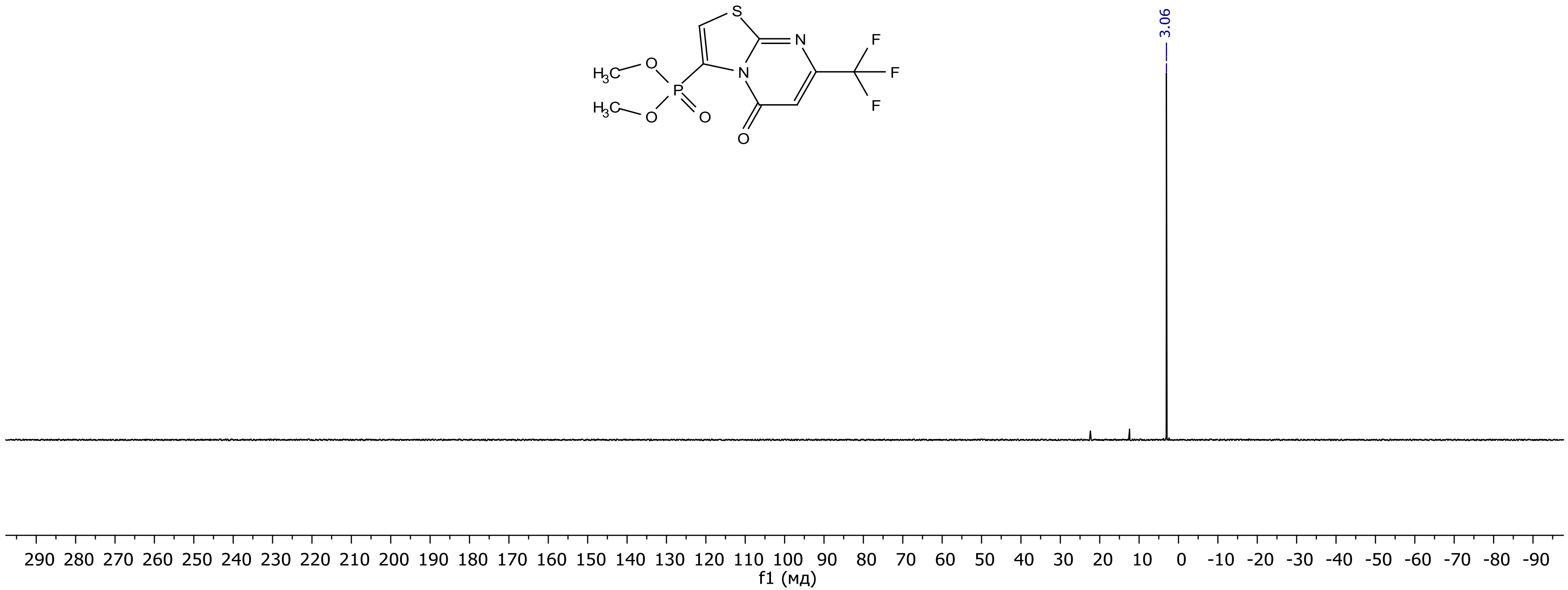

^{31}P NMR spectrum of compound **8a**

Diethyl [5-oxo-7-(trifluoromethyl)-5*H*-[1,3]thiazolo[3,2-*a*]pyrimidin-3-yl]phosphonate (8b)

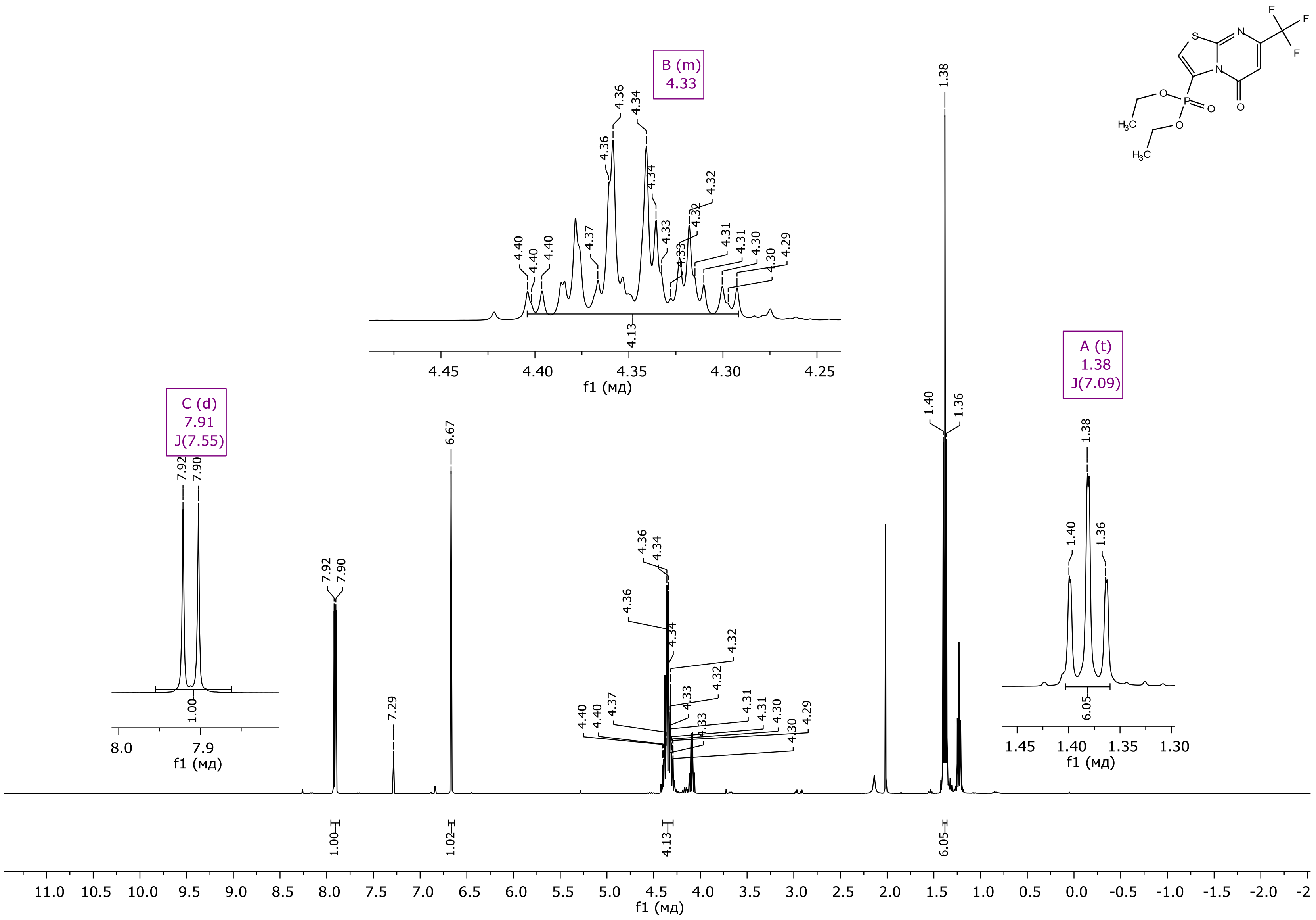

^{1}H NMR spectrum of compound **8b**

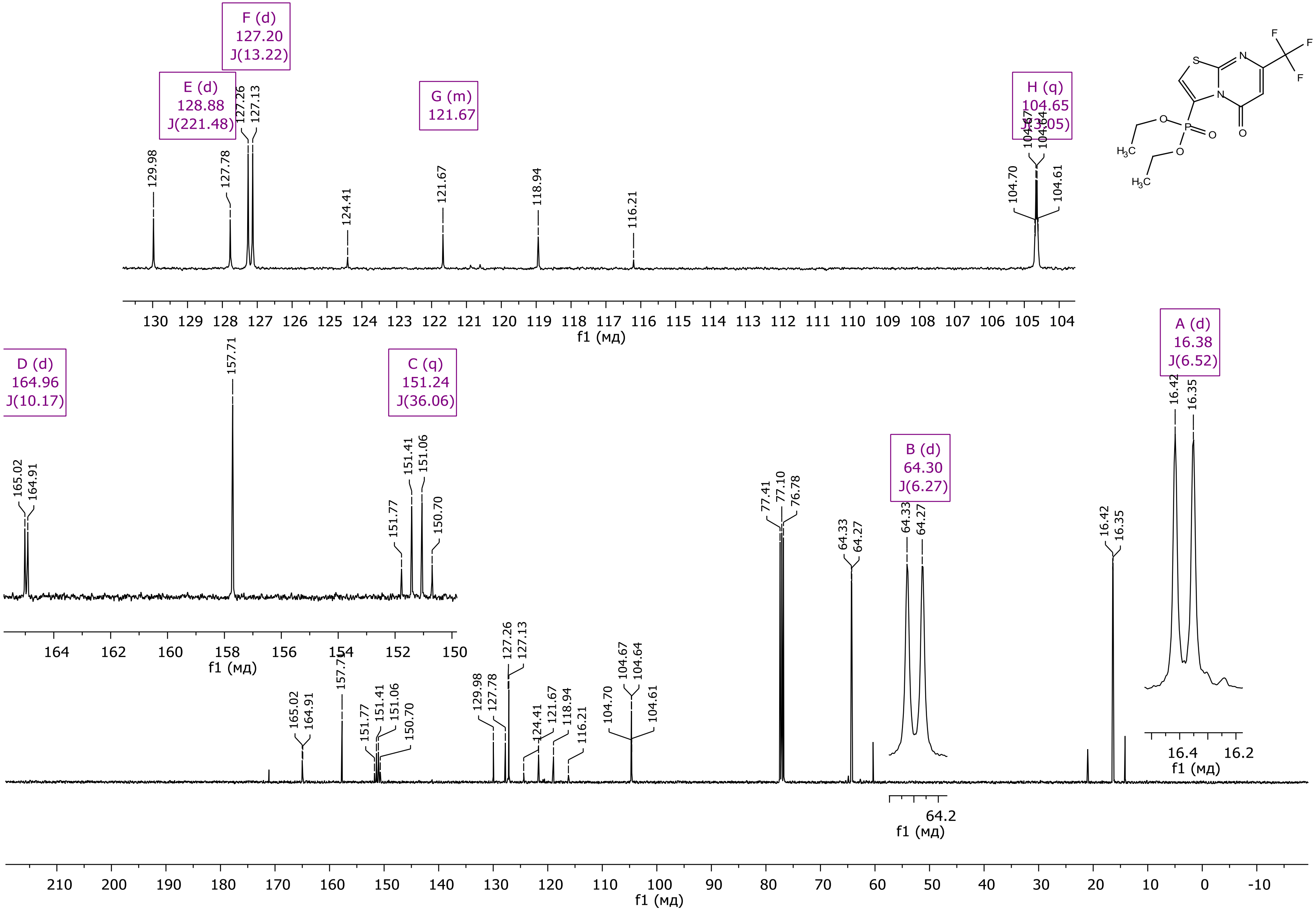

^{13}C NMR spectrum of compound **8b**

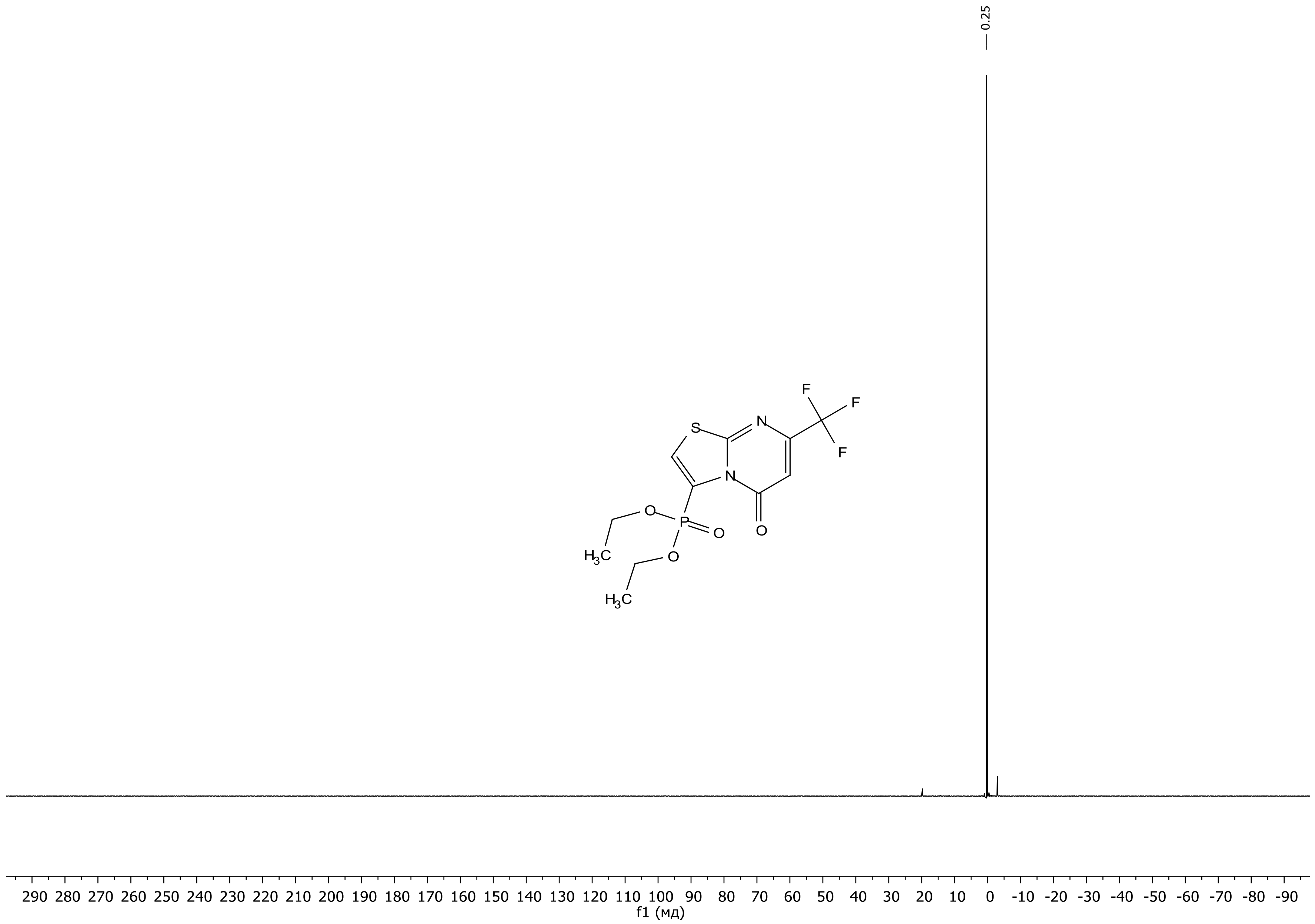

^{31}P NMR spectrum of compound **8b**

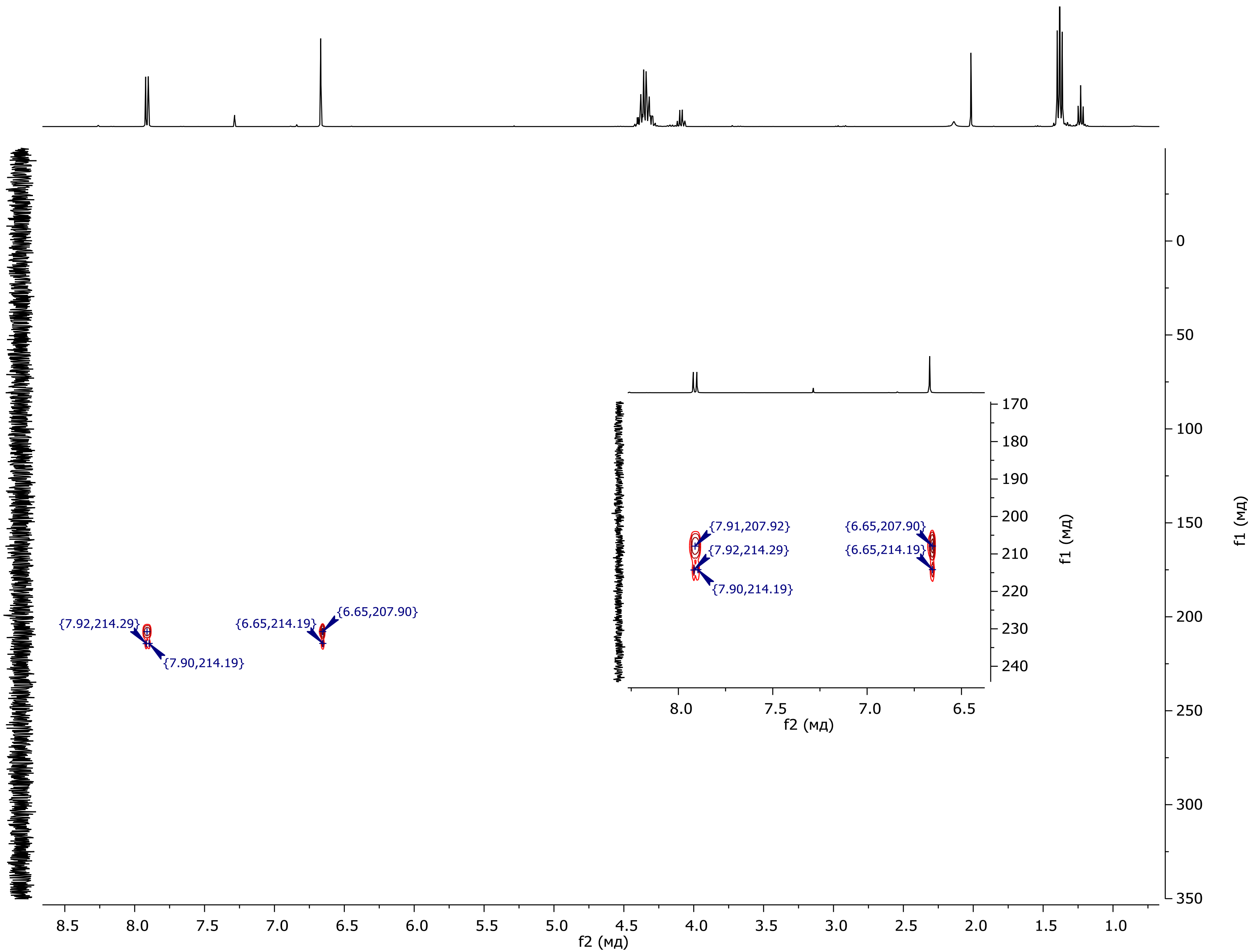

HMBC ^{1}H-^{15}N NMR spectrum of compound **8b**

Diisopropyl [5-oxo-7-(trifluoromethyl)-5*H*-[1,3]thiazolo[3,2-*a*]pyrimidin-3-yl]phosphonate (8c)

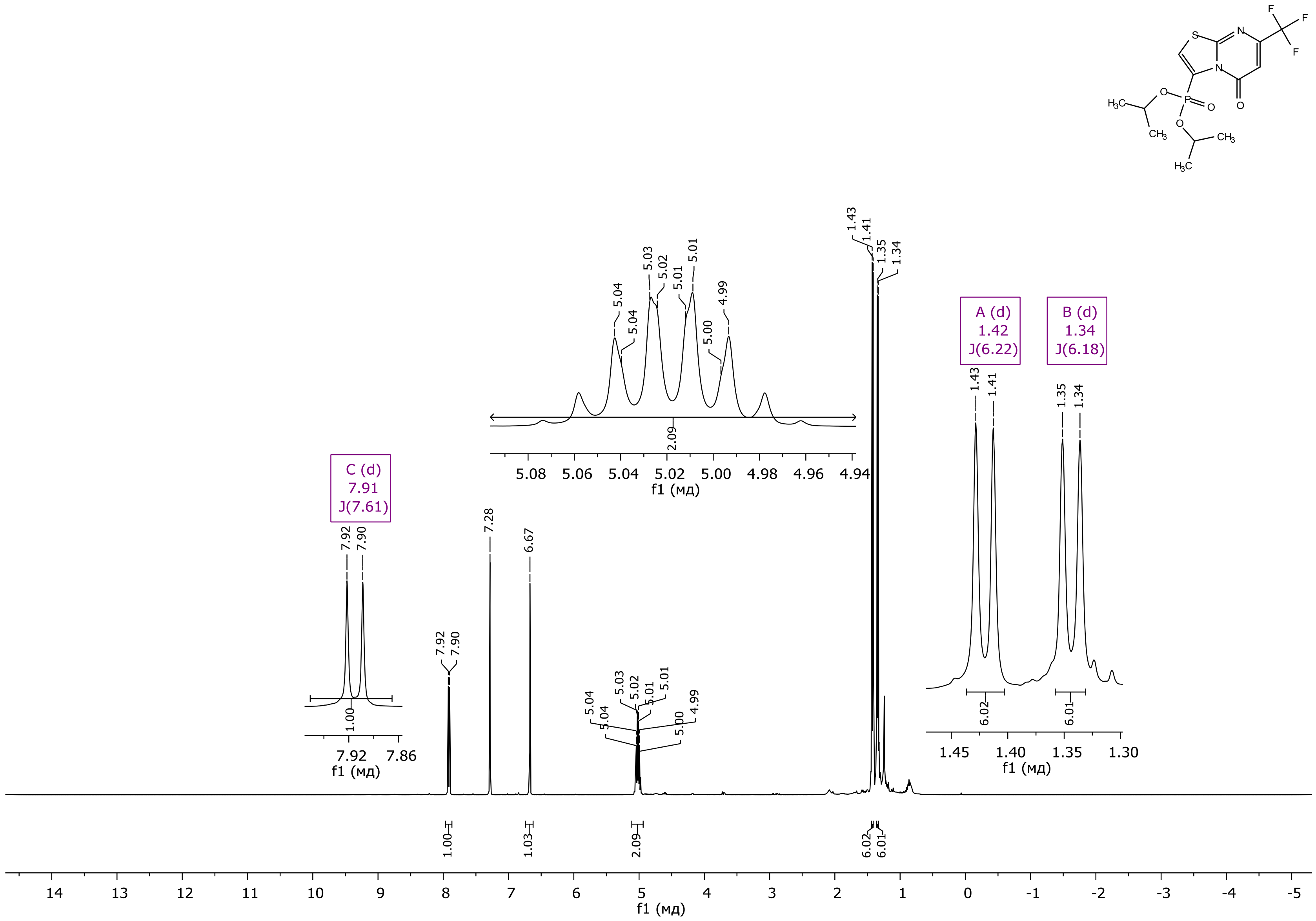

^{1}H NMR spectrum of compound **8c**

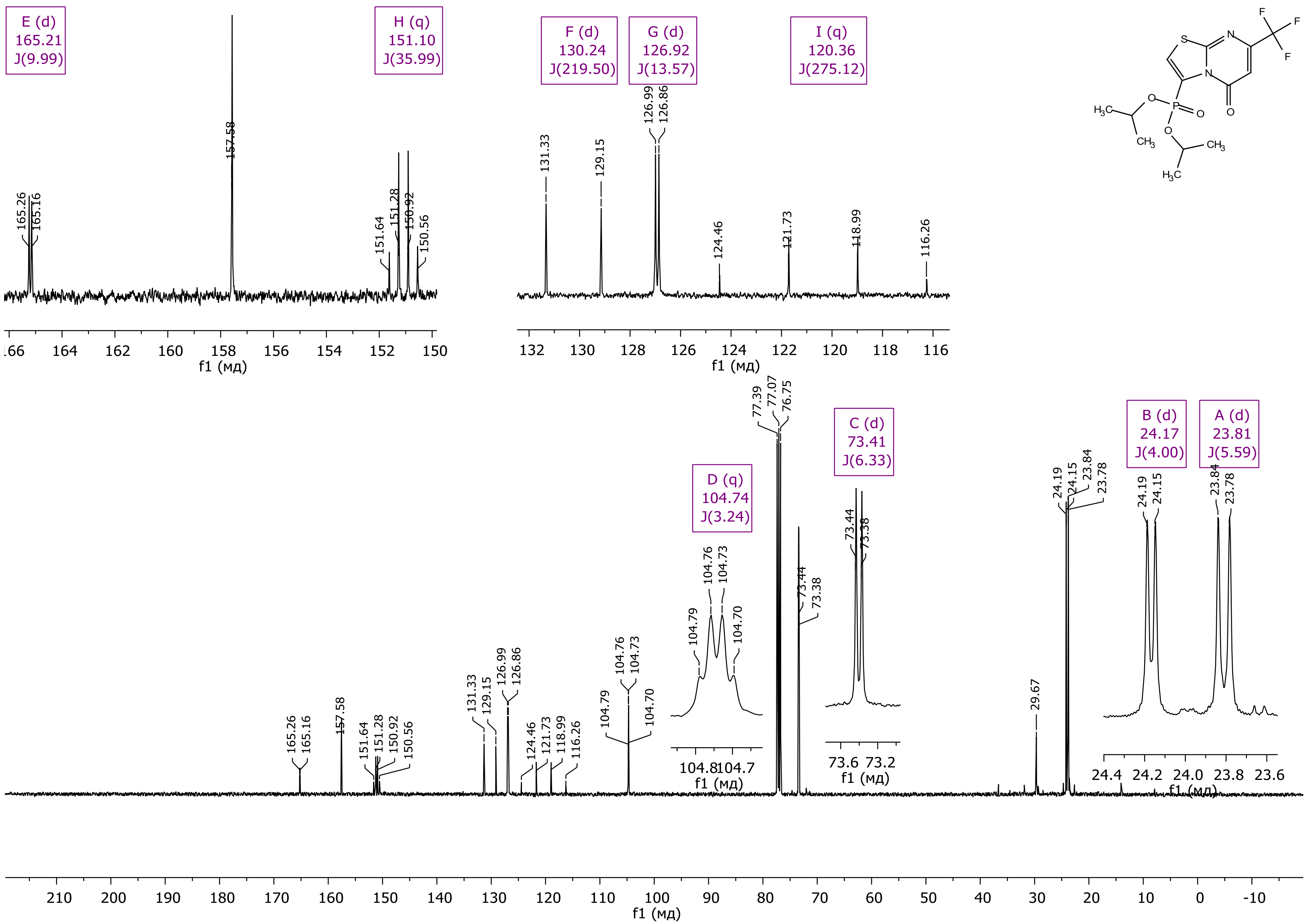

^{13}C NMR spectrum of compound **8c**

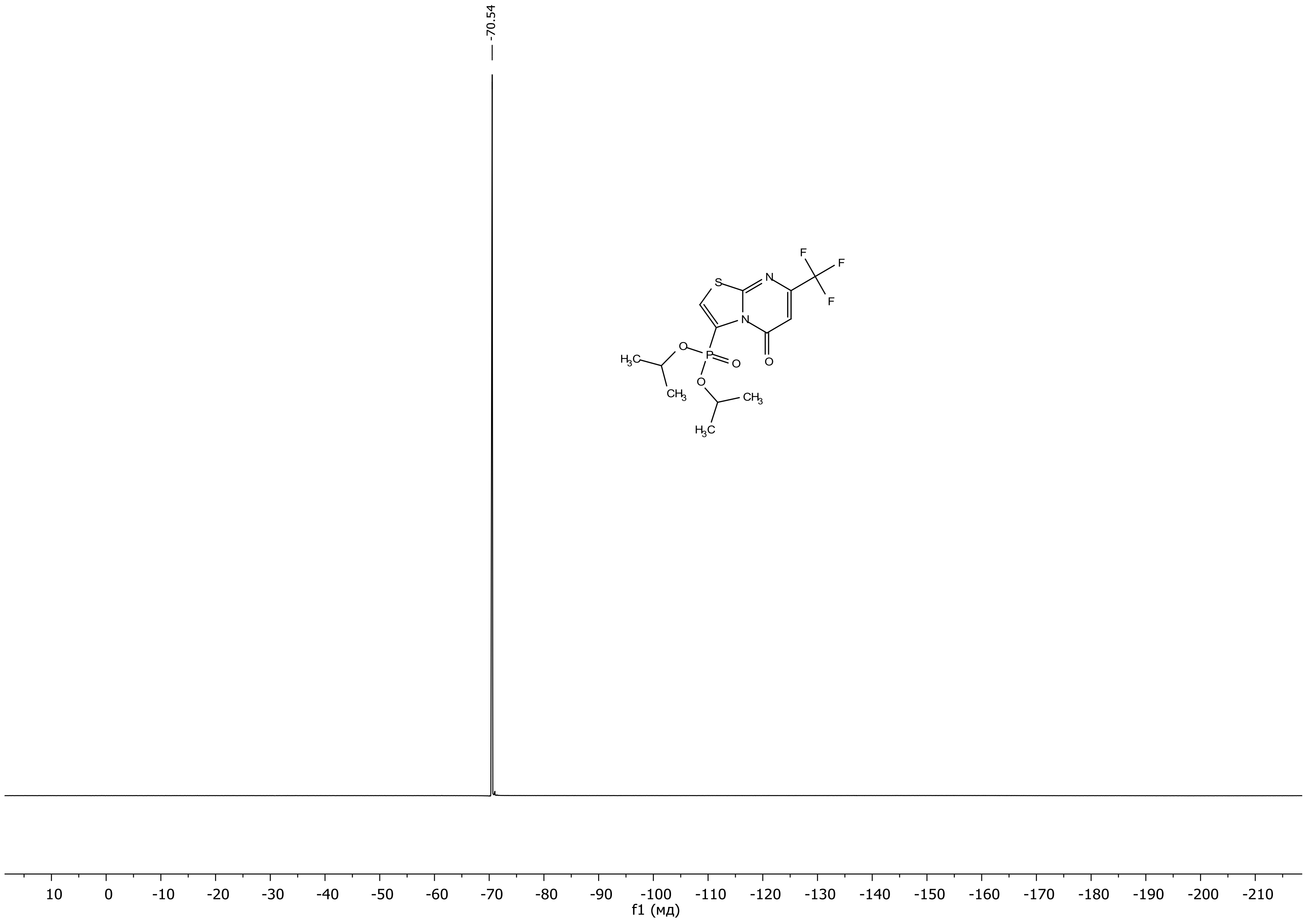

^{19}F NMR spectrum of compound **8c**

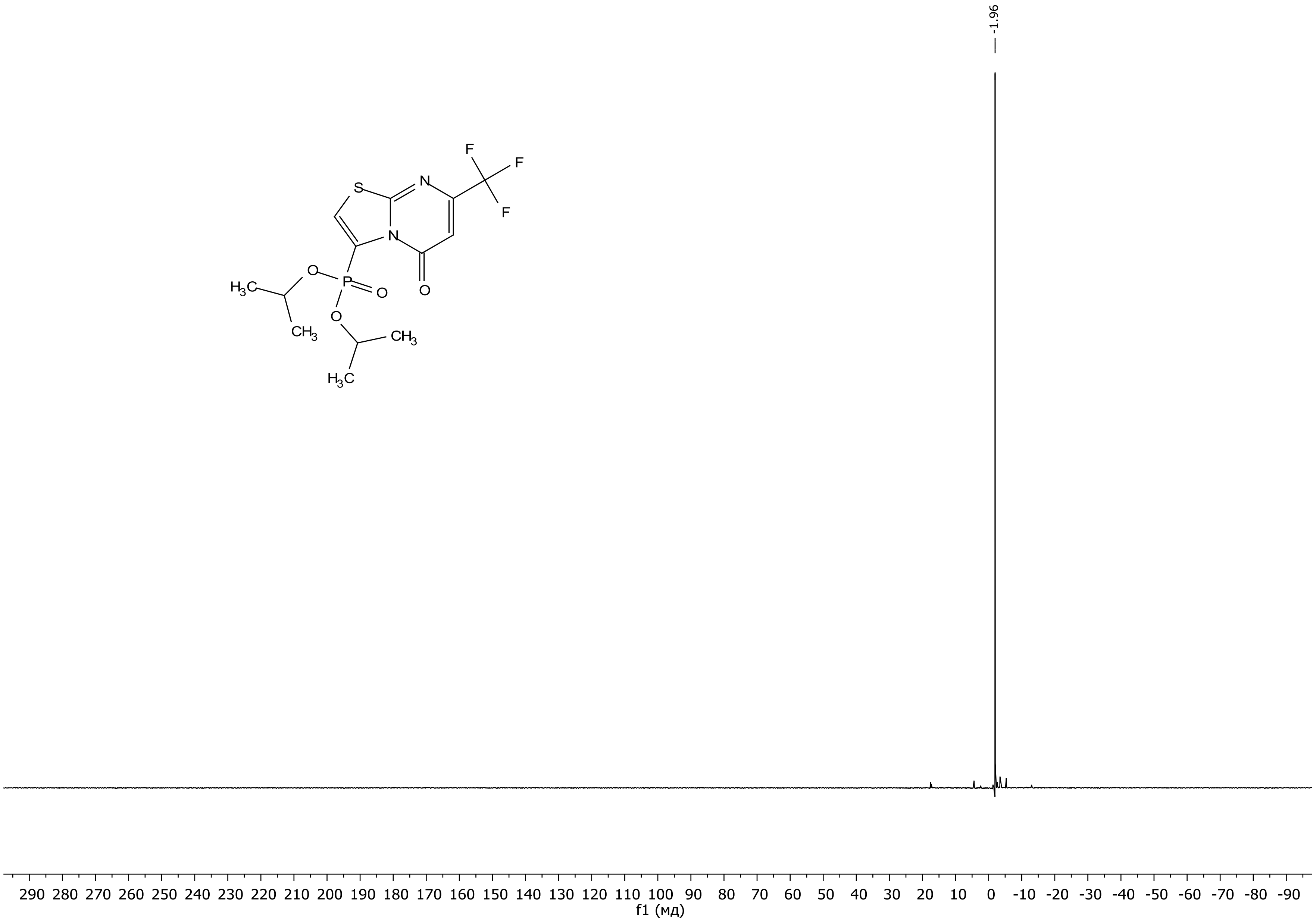

^{31}P NMR spectrum of compound **8c**

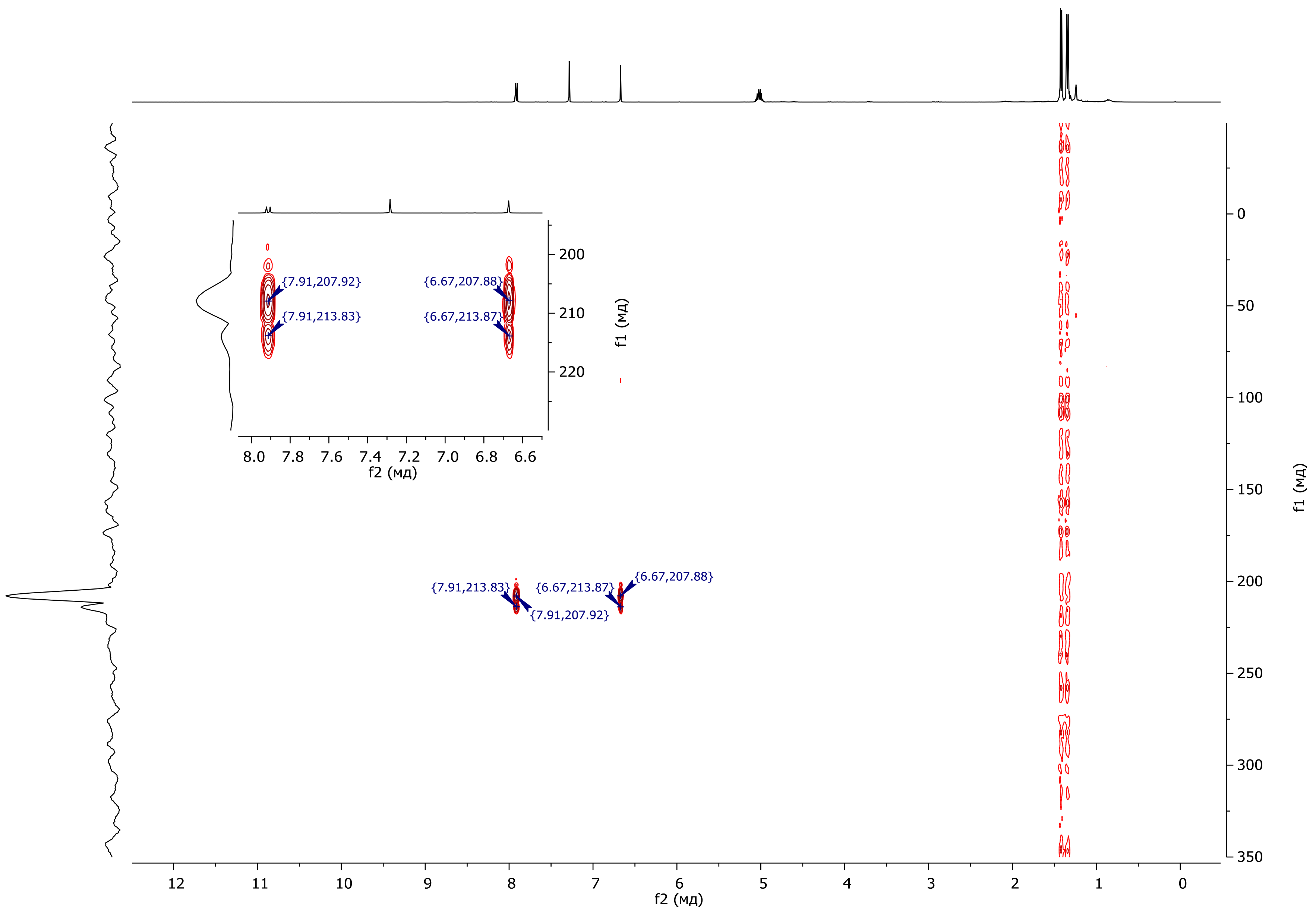

HMBC ^{1}H-^{15}N NMR spectrum of compound **8c**

Diisopropyl [5-oxo-7-(trifluoromethyl)-5*H*-[1,3]thiazolo[3,2-*a*]pyrimidin-2-yl]phosphonate (9c)

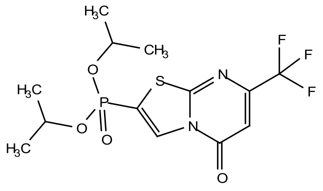

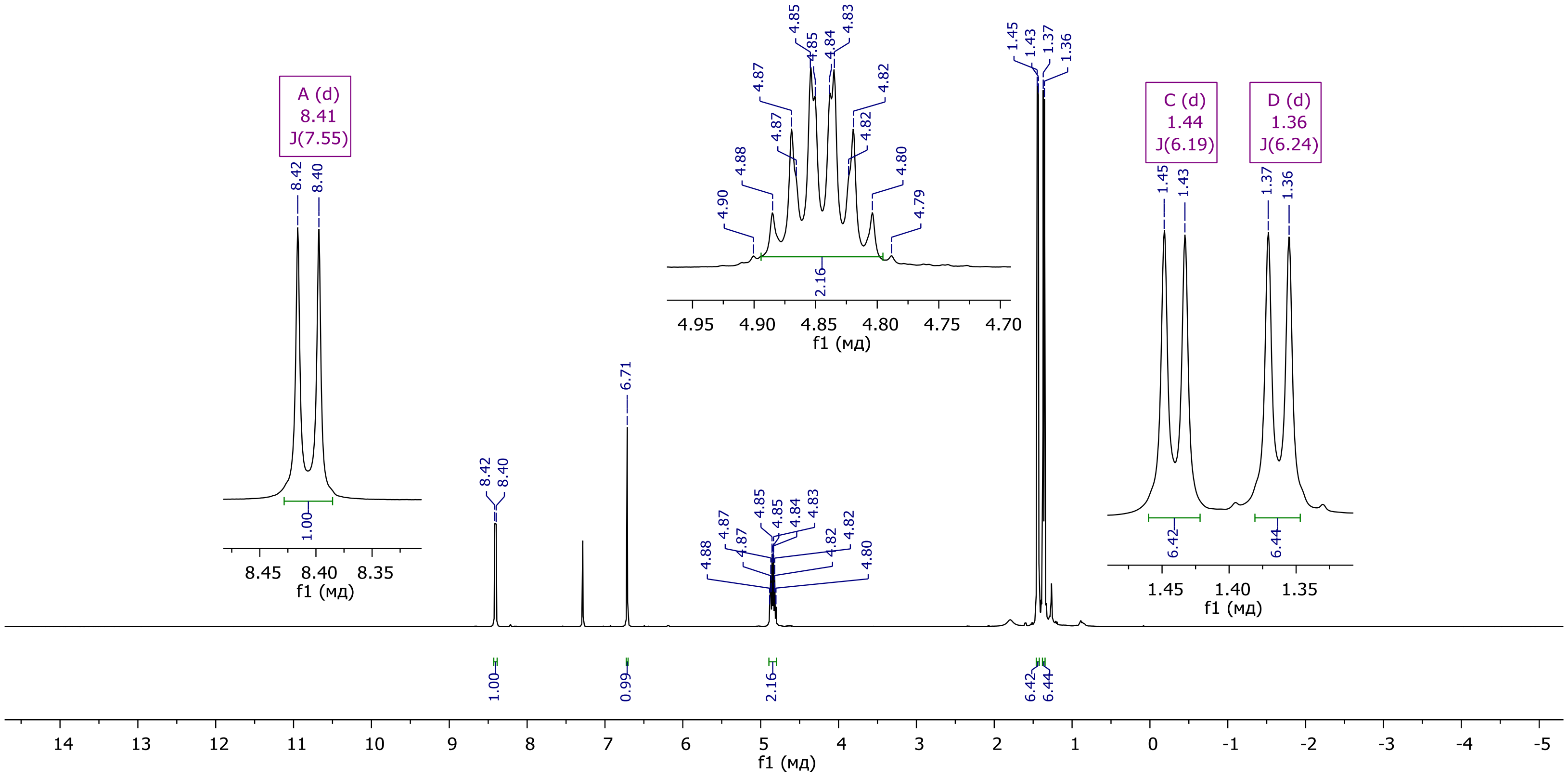

^{1}H NMR spectrum of compound **9c**

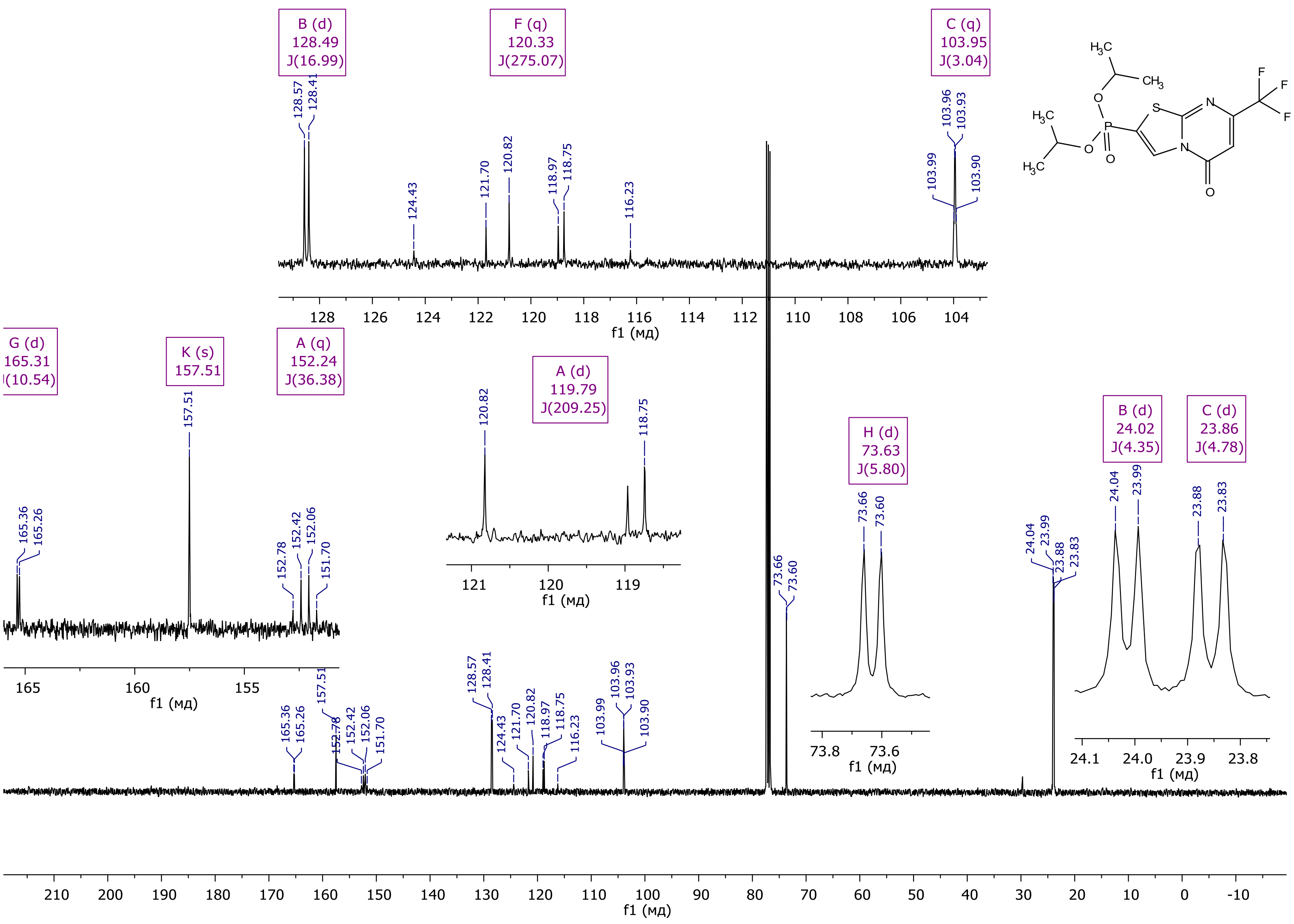

^{13}C NMR spectrum of compound **9c**

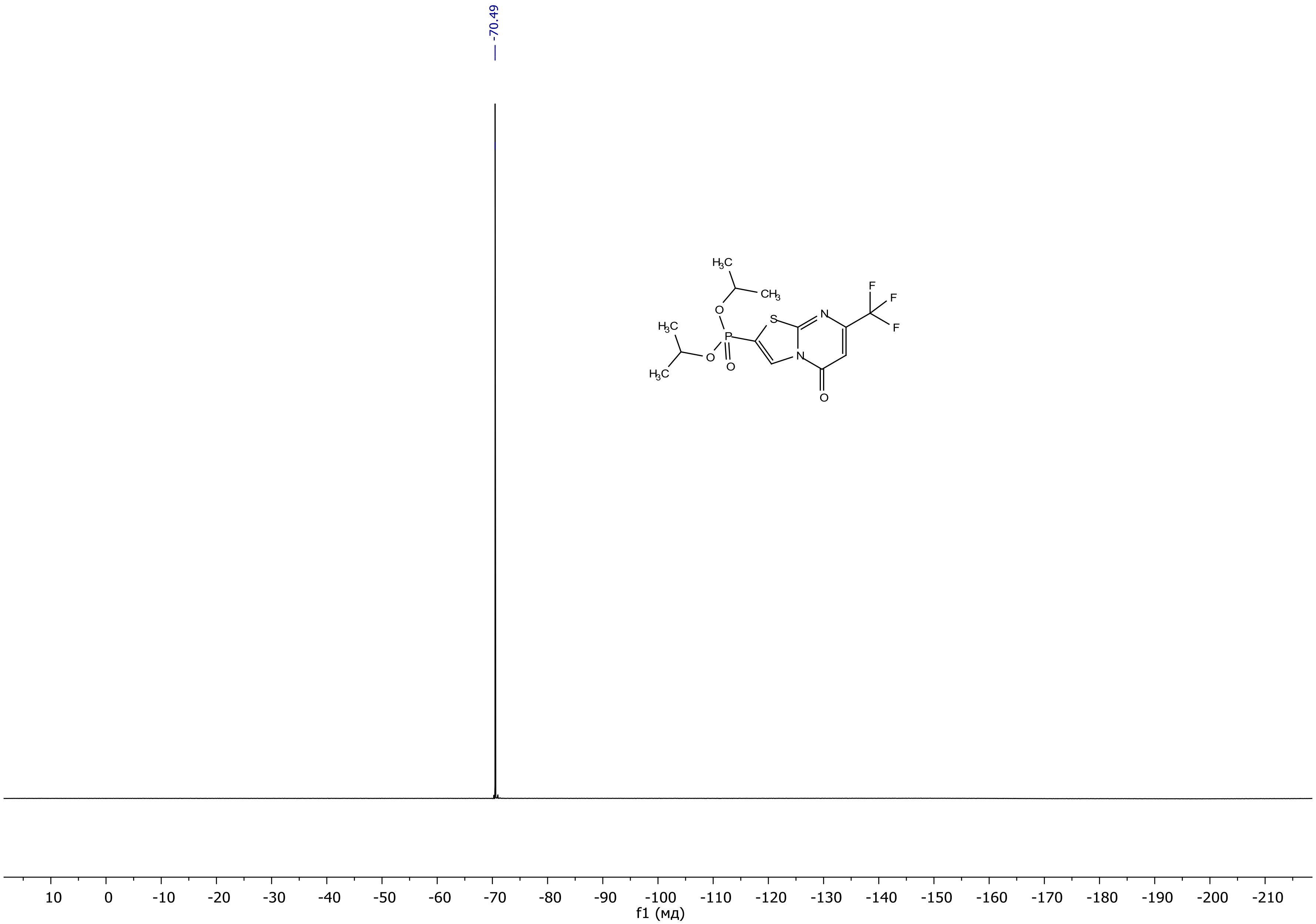

^{19}F NMR spectrum of compound **9c**

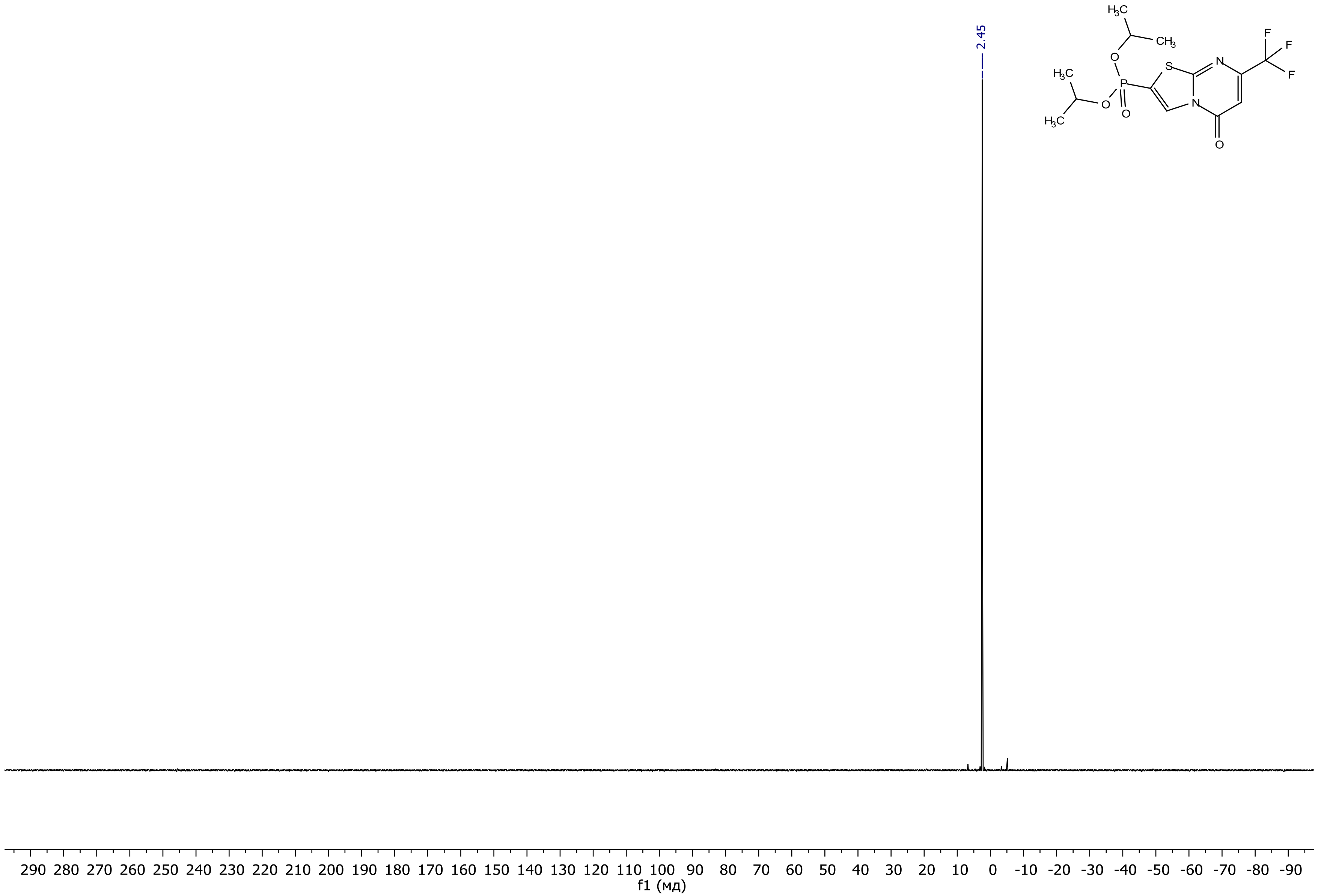

^{31}P NMR spectrum of compound **9c**
